# 1*/f* laws found in non-human music

**DOI:** 10.1038/s41598-023-28444-z

**Published:** 2023-01-24

**Authors:** Adam S. Jermyn, David J. Stevenson, Daniel J. Levitin

**Affiliations:** 1 Kavli Institute for Theoretical Physics, University of California at Santa Barbara, Santa Barbara, CA 93106 USA; 2 Division of Geology and Planetary Science, California Institute of Technology, Pasadena, CA 91125 USA; 3 Department of Psychology, School of Computer Science, and Schulich School of Music, McGill University, Montreal, QC H3A 1B1 Canada

**Keywords:** Applied mathematics, Power law

## Abstract

A compelling question at the intersection of physics, neuroscience, and evolutionary biology concerns the extent to which the brains of various species evolved to encode regularities of the physical world. It would be parsimonious and adaptive, for example, for brains to evolve an innate understanding of gravity and the laws of motion, and to be able to detect, auditorily, those patterns of noises that ambulatory creatures make when moving about the world. One such physical regularity of the world is fractal structure, generally characterized by power-law correlations or 1*/f *^*β*^ spectral distributions. Such laws are found broadly in nature and human artifacts, from noise in physical systems, to coastline topography (e.g., the Richardson effect), to neuronal spike patterns. These distributions have also been found to hold for the rhythm and power spectral density of a wide array of human music, suggesting that human music incorporates regularities of the physical world that our species evolved to recognize and produce. Here we show for the first time that 1*/f*^*β*^ laws also govern the spectral density of a wide range of animal vocalizations (music), from songbirds, to whales, to howling wolves. We discovered this 1*/f*^*β*^ power-law distribution in the vocalizations within all of the 17 diverse species examined. Our results demonstrate that such power laws are prevalent in the animal kingdom, evidence that their brains have evolved a sensitivity to them as an aid in processing sensory features of the natural world.

## Introduction

A compelling question at the intersection of physics, neuroscience, and evolutionary biology concerns the extent to which the brains of various species evolved to encode structural regularities of the physical world^1,2^. It would be parsimonious and adaptive, for example, for brains to evolve an innate understanding of gravity (objects fall down rather than up), of cause-and-effect in physical interactions^3^ (under certain circumstances, colliding with an object causes it to move^4^), and to encode acoustic regularities of the physical world as a precursor to vocal communication and auditory warning systems^5,6^.

What regularities in the physical world might have isomorphic representations in the brain? One candidate regularity is the 1*/f *^*β*^ distribution (or fractal structure), a power law that comprises the Richardson effect^7^. The slope *β* reflects the extent to which a signal autocorrelates over time. White noise, with no temporal correlations, has *β* = 0; by contrast perfectly predictable signals, such as steady tones, have *β* = *∞*. More generally, the greater the value of *β*, the more long-timescale correlations the signal possesses^8^, and the more predictable later portions of the signal are from earlier ones^9^.

1*/f *^*β*^ distributions have been found in musical pitch, melody, radio broadcasts, body movements, brain activity, natural images and geographical features such as coastlines and flood levels of the Nile River^10–13^. Neurons in mammalian brains show preferential coding for 1/*f* signals^14,15^ and the fluctuating voltages across the resting membrane of myelinated nerve fibers show a 1/*f* spectrum^16^. In all these examples, 0.5 ≤ *β* < 1.5, implying an optimal balance between predictability and surprise (so-called surprisals). An innate preference for such distributions would be energy efficient^17^ because it offloads much of the perception of structure from brains to the environment.

Rhythmic and harmonic structure of human-made classical music can also be characterized by 1*/f *^*β*^^9,18^. An emerging consensus is that humans make music the way we do because it reflects structural regularities of the physical world that we evolved to be sensitive to^19,20^—in particular, 1*/f *^*β*^ structure.

If humans evolved a sensitivity to 1*/f *^*β*^ sound structures, perhaps animals did too. Here we sought to address this question using a wide variety of animal calls and songs, including songbirds, whales, howling wolves, frogs, and others.

All sound begins as oscillations in pressure and particle displacements that propagate through an acoustic medium such as air or water^21^. Animals produce a wide variety of sounds for signaling, communication, and echolocation, using highly variable acoustic structures that range from almost perfectly periodic vocal-fold vibration to stochastic noise^22^. Vocalizations, restricted to vertebrates, originate in the respiratory system, while other sounds are produced mechanically by the interaction of body parts with themselves or the surrounding environment^23^. Sound reception of those signals in animals primarily entails mechanosensory organs responding to airborne signals, and vibratory (biotremological) sensing^24^. Our starting point is the hypothesis that across the diverse range of ways animals produce and perceive sounds^25–27^, there may be common organizing principles underlying all of these, and 1/*f*^*β*^ is such a candidate. Finding a common analysis method across all these sounds has presented a challenge to comparative bioacoustics^28,29^ that we address here.

We begin with the mathematical basis for this work. A conceptual, non-mathematical way to think about 1/*f *^*β*^ functions is that they model autocorrelation/self-similarity. Human music and communication are well-modeled by these functions, which are scale-free and quantify the degree of randomness versus structure in the signal^8,9,30^. Communication in both music and speech has correlations that extend over all time scales and as such, 1/*f *^*β*^ functions describe both perceptible and imperceptible aspects of the signal. An obvious case of an autocorrelation in music is the repetition of notes, and in speech in the repetition of words or sound patterns (particularly noticeable in poetry and some oratory). Many 1/*f *^*β*^ patterns so far discovered are latent.

We then describe our methods our findings. All species and nearly all recordings examined showed a strong preference for 1/*f *^*β*^ laws over ones with short-ranged correlations, and the spectral index *β* was found to vary among species. Indeed, while the overall magnitude of this index was generally comparable across species, we show that it is possible to distinguish between species in our sample purely using this index, much as prior work showed that it could distinguish among human composers^9^. Furthermore, we find that this spectral index is correlated with whether the recordist referred to the recording with words such as “song”, such that the more similar the index was to typical values in human music the more likely the use of these musical descriptors.

Finally we discuss the potential evolutionary implications of our findings. The fact that nearly every recording we examined was best fit by power-law correlations reinforces the universality of this phenomenon, though of course it is not possible to prove causation in this domain.

## Mathematical basis

A key indicator of fractal structure is a power-law spectrum. For a time-varying signal of amplitude *s*(*t*) the spectrum is given by

1 $$\tilde{s}\left( f \right) \equiv \smallint s\left( t \right)e^{ - 2\pi ift} dt.$$

The instantaneous power of such a signal is

2 $$p\left( t \right) \equiv s^{2} \left( t \right),$$

which has spectrum

3 $$\tilde{p}\left( f \right) \equiv \smallint s\left( t \right)^{2} e^{ - 2\pi ift} dt$$

In this language, a signal generated by fractal processes has power spectrum

4 $$\tilde{p}\left( f \right) \propto \frac{1}{{f^{\beta } }}$$

for some spectral index *β* > 0. This power spectrum is related to the time autocorrelation of the signal, such that $\tilde{p}\left( f \right)$ describes the strength of correlations on a time-scale *f *^*−*1^^31^. Processes which encode long-range correlations must exhibit such a spectrum (in this context, long-range means that the amplitude of the autocorrelation at time τ must fall off at most as a power-law in τ. It is not a statement about the characteristic magnitude of these correlations). By contrast processes exhibiting short-ranged correlations cannot exhibit such a spectrum and must fall off more steeply with frequency. Hence, $\tilde{p}\left( f \right)$ may be used to discriminate between long-ranged processes and those with finite-ranged correlations such as auto-regressive models^32^.

Our hypothesis is that animal vocalizations exhibit power spectra consistent with long-range correlations. In this work, we test this against the null hypothesis that animal vocalizations are generated by finite-memory autoregressive processes. Such processes are a superset of both white noise and a broad range of oscillatory and exponential phenomena with bounded memory, and so provide a good model for phenomena with only short-ranged correlations. We also test the secondary hypothesis that the animal vocalizations which human listeners identify as musical have power spectra more similar to human music than those which they do not identify as musical.

The result is a comparison between fractal power-laws and short-range autoregressive generating processes. The former require long-range structure and can potentially encode complex information, while the latter are characteristic of processes with limited memory, and hence limited-range correlations.

## Results

### Posterior distributions

**Figure 1:**
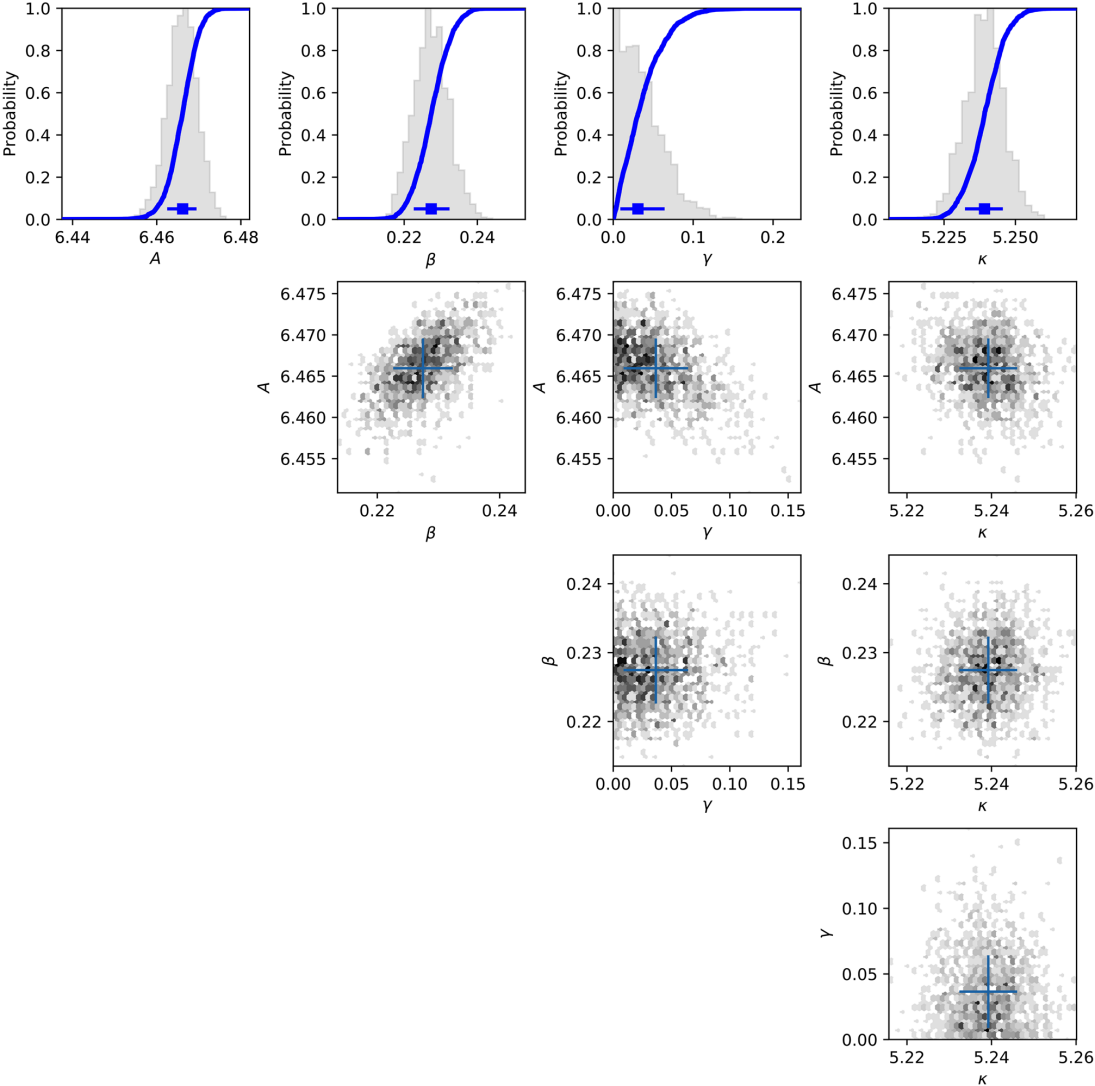
The posterior distribution of the fractal model parameters is shown in a corner plot of two-dimensional cuts and one- dimensional histograms for Recording 123,136 of the Killer Whale. The distribution has been marginalized over the parameters which are not shown. Blue crosses show the mode of the distribution as identified by the Multinest algorithm.

**Figure 2:**
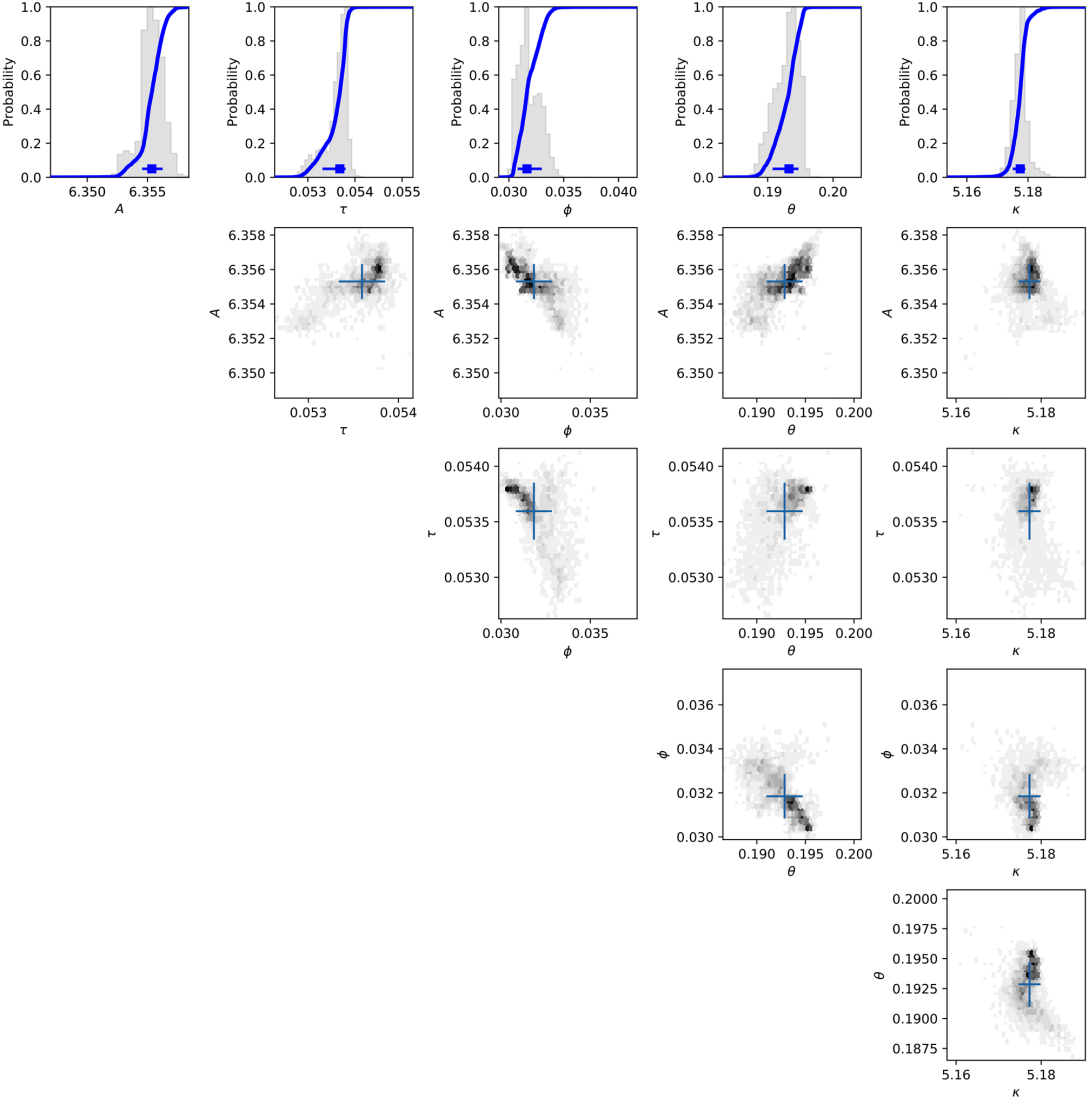
The posterior distribution of the ARMA model parameters is shown in a corner plot of two-dimensional cuts and one- dimensional histograms for Recording 123,136 of the Killer Whale. The distribution has been marginalized over the parameters which are not shown. Blue crosses show the mode of the distribution as identified by the Multinest algorithm.

The principal output of our analysis is the posterior distribution over the model parameters. Figure 1 shows the posterior distribution of the fractal model parameters for one recording. Figure 2 shows the same for the ARMA model.

Whenever the posterior peaks towards a boundary of the prior domain, it does so towards a boundary which is known a priori. For instance, the presence of background white noise cannot produce an offset which is negative, so the fact that *γ* shows a peaked distribution towards zero in Fig. 1 simply indicates that that recording is consistent with having no such noise. If a parameter converged to one of the boundaries which was not logically fixed that would constitute evidence that the optimal fit lies outside of the prior domain. This has not happened, which suggests that our choice of prior space has not resulted in any significant omissions or distortions.

In both models, and for each mode there is significant cross-correlation between several parameters. In the fractal model the spectral index *β*, the amplitude *A* and the offset *γ* are very strongly correlated. This is not surprising because a change in the fitted slope may be partially offset by a change in amplitude or shape. However, it does mean that in analyzing differences between recordings the cross-correlation between parameters cannot be neglected, and it is often a good approximation to say that there is just one independent parameter.

#### Bayesian evidence

In addition to the posterior distribution, our procedure produces the Bayesian evidence *Z* for each model. While the overall scale of *Z* is not meaningful, being tied to the choice of prior normalization, ratios of *Z* across different models represent ratios of the posterior probabilities of those models. That is, within the space of just ARMA and Fractal models,

5 $$\frac{{P\left( {{\text{ARMA}}} \right)}}{{P\left( {{\text{Fractal}}} \right)}} = \frac{{Z_{{{\text{ARMA}}}} }}{{Z_{{{\text{Fractal}}}} }},$$

6 $$\log \frac{{P\left( {{\text{ARMA}}} \right)}}{{P\left( {{\text{Fractal}}} \right)}} = \log Z_{{{\text{ARMA}}}} - \log Z_{{{\text{Fractal}}}}$$

where in each case *P* (model) refers to the posterior probability of a model. The second column of Supplementary Information Table 2 shows the sum of this logarithmic likelihood ratio over all recordings for each species. This is an estimate of the extent to which the data favor the ARMA model over the fractal one. Hence the large negative values, ranging from − 6 × 10^3^ to − 10^5^, indicate a very strong preference for the fractal model for all species. Furthermore, as shown in Supplementary Information Table S3, all but 24 recordings show a preference in this direction. There is therefore good reason to believe that these animal vocalizations do indeed follow a fractal law with long-range correlations.

While this evidence is extremely strong, suggesting likelihood preferences of order *e*^5*,*000^ in favor of the fractal model, there are several caveats to consider. First, the model space we have examined is quite simple. It is possible for the ARMA model to be strongly disfavored while another as-yet undiscovered model does better than the fractal one. Nevertheless, because these models are representative of two qualitatively different kinds of correlations, namely short- and long-ranged respectively, it is highly suggestive that the latter is so strongly preferred.

The second caveat is that the evidence ratio may depend strongly on our model of the noise. For instance, if we have underestimated the noise by a factor of 2, then the logarithmic evidence has been overestimated by an amount of order *N* ln 2, where *N* is the number of samples in the spectrum. However, the fitted parameter *κ* in each case accounts for the possibility of under- or over-estimated noise. There is good agreement in *κ* between the two models and little correlation between *κ* and the other parameters, which suggests that the magnitude of the noise is well-captured in our calculations. Hence this likely does not explain the difference in Bayesian evidence.

**Figure 3:**
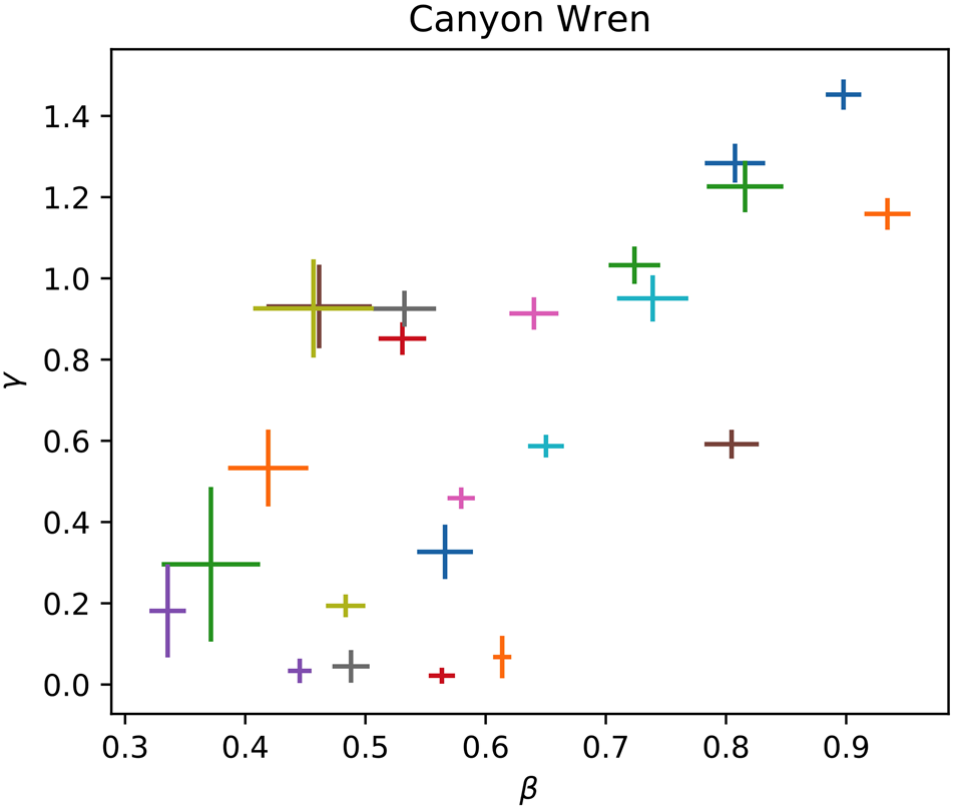
The inferred fractal parameters *γ* and *β* are shown for all recordings of the Canyon Wren that were analyzed. These are broadly consistent up to their uncertainties.

**Figure 4:**
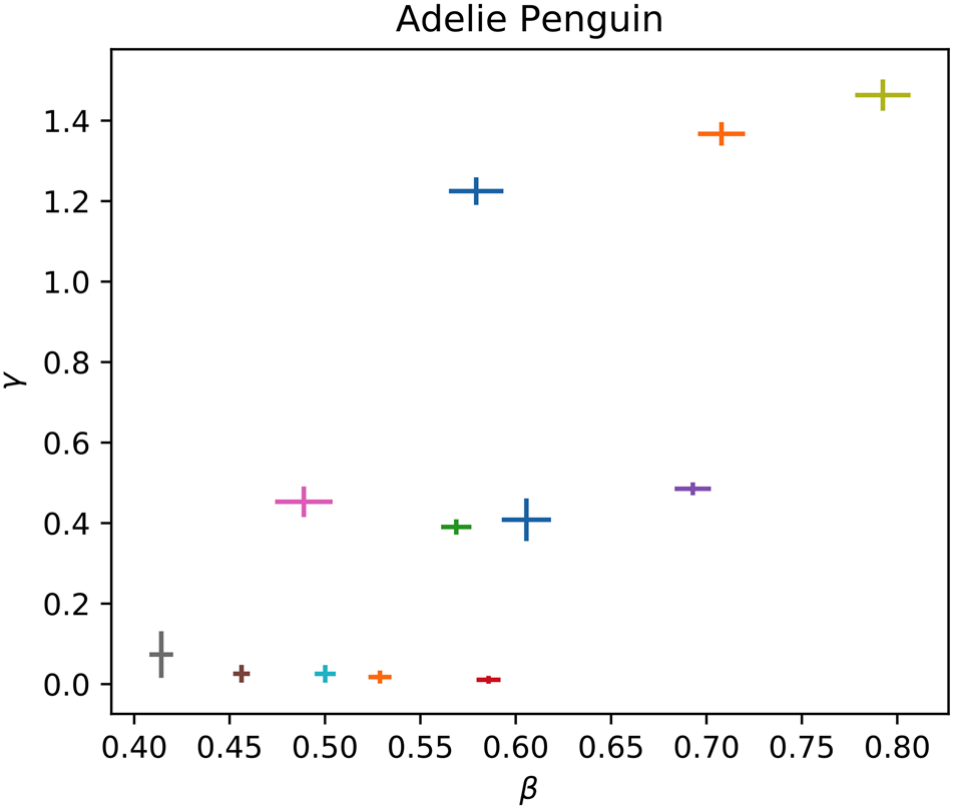
The inferred fractal parameters *γ* and *β* are shown for all recordings of the Adelie Penguin which were analyzed. Note that there seem to be three populations with distinct parameters, namely one in the bottom-left, one in the middle, and one in the right-right.

Finally, even though nearly all recordings favor the fractal model, it is not correct to simply sum the logarithmic evidence across recordings if one believes that the model parameters are species-specific rather than recording-specific. Any universality claim must contend the former rather than the later, and so predicts that the parameters inferred for different recordings of the same species are consistent. If we find that this is not the case, then there must be some scatter between recordings owing either to underestimated noise or to non-universal features in the vocalizations. This is consistent with what we see. Figures 3 and 4 show the scatter in the fractal parameters *γ* and *β* for different recordings of the Canyon Wren and Adelie Penguin respectively. Both are representative species for these purposes. In the former the scatter is roughly consistent with the individual measurement uncertainties. In the latter the scatter is significantly in excess of these, and suggestive of multiple distinct classes of vocalizations.

#### Intrinsic variability

To quantitatively understand the intrinsic variability of the population we first compute best weighted estimates of the mean, its standard deviation, and the population standard deviation, given respectively as

7 $$\mu \equiv \frac{{\mathop \sum \nolimits_{i} x_{i} \sigma_{i}^{ - 2} }}{{\mathop \sum \nolimits_{i} \sigma_{i}^{ - 2} }},$$

8 $$\sigma_{\mu } \equiv \left( {\mathop \sum \limits_{i} \sigma_{i}^{ - 2} } \right)^{ - 1/2}$$

and

9 $$\sigma_{{{\text{pop}}}} \equiv \frac{{\mathop \sum \nolimits_{i} \left( {x_{i} - \mu } \right)^{2} \sigma_{i}^{ - 2} }}{{\mathop \sum \nolimits_{i} \sigma_{i}^{ - 2} }}.$$

These are reported in the final three columns of SI Table S2. Parameters for individual fits are given in SI Tables S4 and S5.

If the scatter in a population is consistent with the uncertainties in the posterior distribution and all variables are normally distributed, then

10 $$\frac{1}{M - 1}\mathop \sum \limits_{i} \frac{{\left( {\beta_{i} - \mu_{\beta } } \right)^{2} }}{{\sigma_{{\beta_{i} }}^{2} + \sigma_{\beta ,\mu }^{2} }} \approx 1,$$

where *M* is the number of recordings. We focus on the distribution of *β* here because the amplitude depends on how the recording was produced and *γ* just amounts to a frequency cutoff which could be attributed to other signals present in the recording environment. SI Table S6 shows the left-hand side of Eq. (10) evaluated for each species. Every species has the left-hand side of this equation considerably greater than the right-hand side, in ratios ranging from 75 to 10^4^, which indicates that there is significant intrinsic variation between recordings of a given species.

A similar result is obtained by a Kolmogorov–Smirnov test against the normal distribution with mean *µ*_*β*_ uncertainty *σ*_*β,*pop_. The results of this are shown in SI Table S7. The Bach recordings cannot reject the normal hypothesis because there are only two, and so they are guaranteed to be consistent with the population mean and variance. For every other species it is possible to reject this distribution at *p* < 0*.*05, and for many the confidence is much higher still. This further points to variation amongst recordings which is not included in our model.

To determine if this extra variation is indicative of sub-types of recordings consider the model distribution

11 $$P\left( \beta \right) = \frac{{\mathop \sum \nolimits_{i = 1}^{N} A_{i} {\mathcal{N}}\left( {\mu_{i} ,\sigma_{i} } \right)}}{{\mathop \sum \nolimits_{i = 1}^{N} A_{i} }},$$

where *A*_*i*_ are positive model-parameters and *N* (*µ, σ*) denotes the standard normal distribution with mean *µ* and standard deviation *σ*. performed Bayesian inference to fit this model to the set of *β* derived f or each species. We took a uniform prior over [0*,* 4] f or each *µ*_*i*_ and over [0*,* 1] f or each *σ*_*i*_. We restricted the prior space so that *µ*_*i*_ < *µ*_*j*_ for *i* < *j* as to avoid allowing duplicated modes. We took a log-uniform prior over [10^*−*5^*,* 10^5^] f or each *A*_*i*_. We did this f or *N ∈ {*1*, …,* 5*}* and report the evidence in SI Table S8.

In all cases a single mode is the best fit with moderate evidence in its favor. The weakest case is that of the Gray Catbird, which has only ∆ l n *Z≈ − *0*.*3 in favor of a single mode over two modes. Hence with this possible exception the recordings appear to come from a single distribution, just one with more variation than our noise model explains. To verify these results, we applied the same methods to simulated datasets with multiple modes and were able to consistently determine the number of modes. So there likely is just one mode in most cases. This could mean that the noise has different statistics in the tail of the distribution than what we have assumed, such that a normal distribution is not a good fit.

Regardless of any intrinsic variation between recordings, the mean of *β* and its standard deviation strongly suggest that different species favor different regions of parameter space. For instance, our sample of recordings from the Adelie Penguin suffices to distinguish it from the Canyon Wren or Bach on spectral index alone, similar to the way that one of us, in a prior paper, could distinguish Mozart from Beethoven^9^. Furthermore, species in the same family are more similar in spectral index to one another than to those of other families, so that this index suffices to distinguish, for instance, members of the Fringillidae family from the Old World Flycatchers. Hence, we conclude that there are long-term correlations in the vocalizations of these species which are thus far best-modelled by a fractal power-law with species- and family-specific parameters.

#### Individual fits

**Figure 5:**
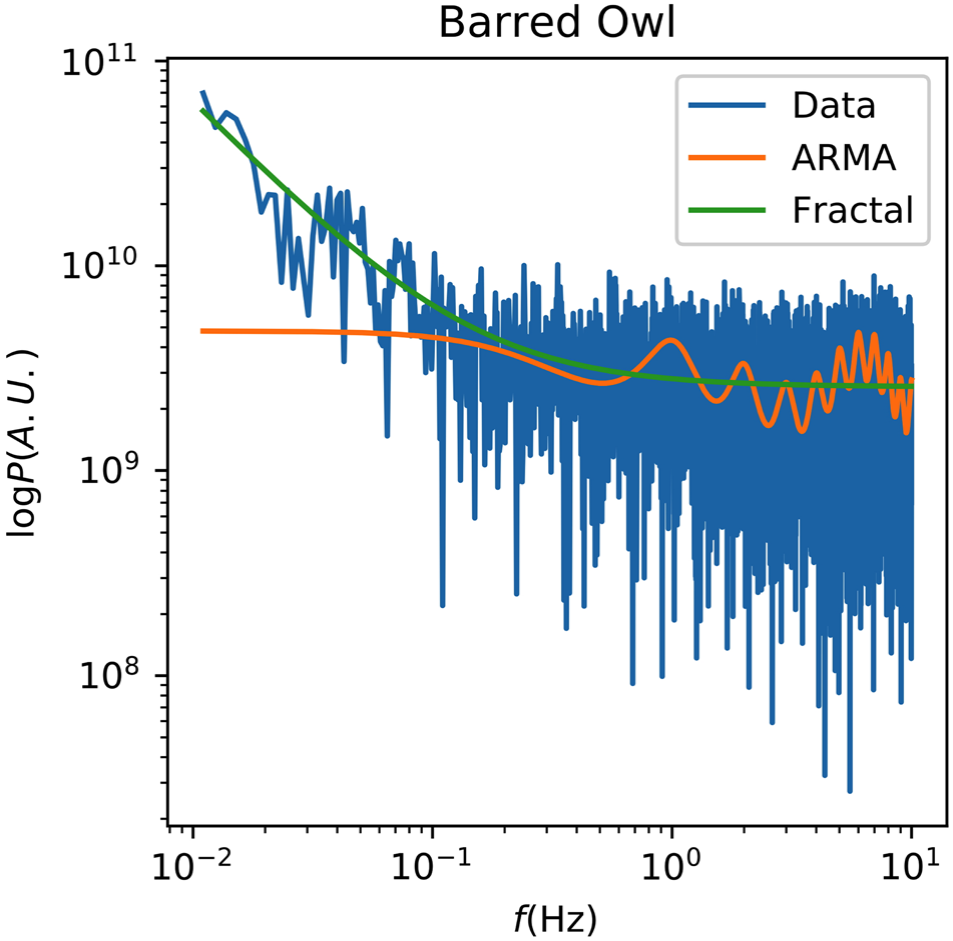
The spectrum of Recording 128,925 of the Barred Owl is shown along with the best-fit ARMA and fractal spectral models.

It is next worth examining the best-fit solutions for individual recordings. Figure 5 shows these for a recording of the Barred Owl along with the spectrum of the recording. Neither model captures the detailed features of the data, but the fractal model does a better job of fitting the trend both at high and low frequencies. The same is visible in Supplementary Information Fig. S2, where the fractal model has a clear advantage, particularly at the low-frequency end.

**Figure 6:**
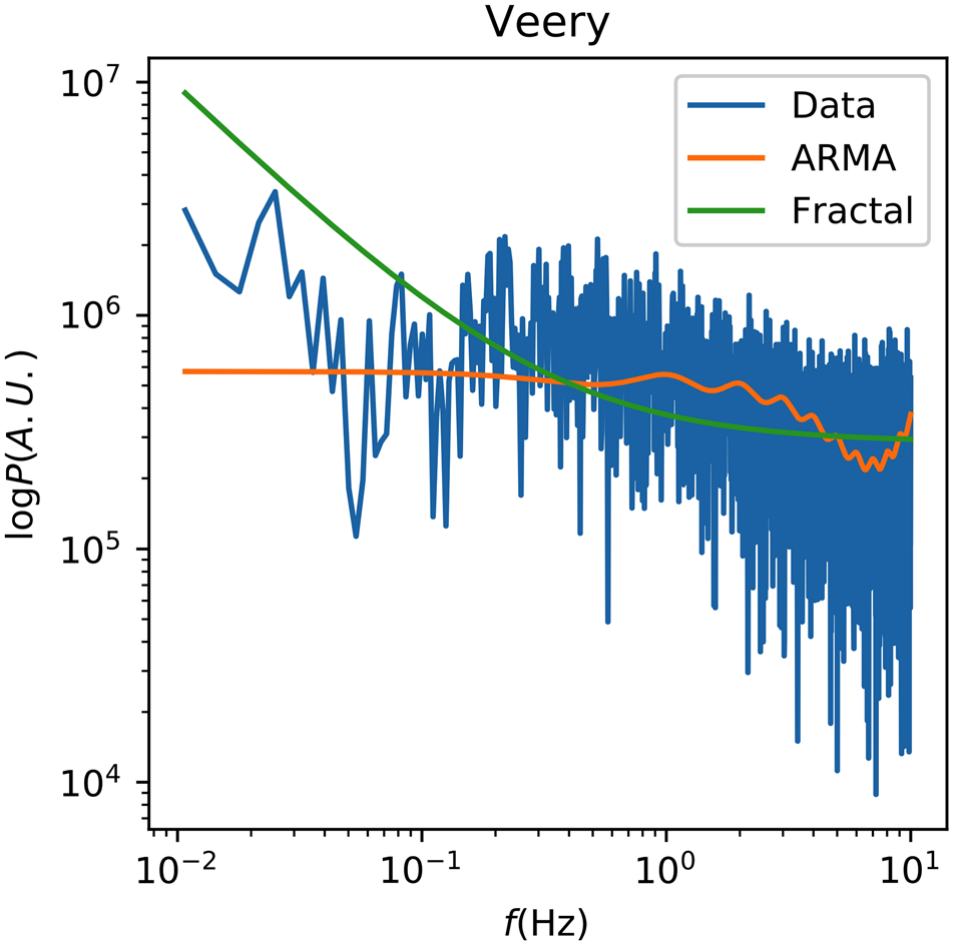
The spectrum of Recording 135,727 of the Veery Thrush is shown along with the best-fit ARMA and fractal spectral models.

**Figure 7:**
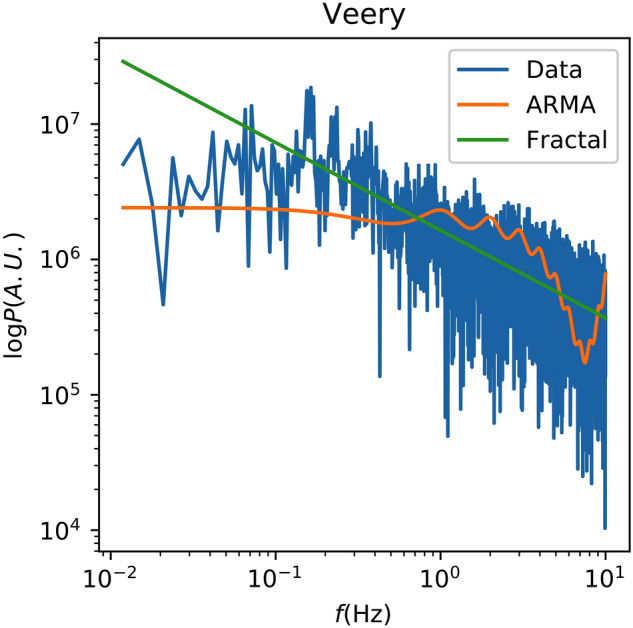
The spectrum of Recording 27,193 of the Veery Thrush is shown along with the best-fit ARMA and fractal spectral models.

By contrast, Fig. 6 shows a more promising fit with the ARMA model for the Veery Thrush. For this recording the ARMA model produces a substantially better fit to the data than the fractal one, successfully reproducing both the high-frequency structure and the low-frequency plateau, albeit at the wrong amplitude. This suggests that most of the structure in this recording is confined to short timescales. The best-fit has *τ ≈* 0*.*072 s, which is evidently the scale of that structure. Such cases are rare (see Supplementary Fig. S3 for another example), however, and do not generally reoccur across different recordings for the same species. For instance, Fig. 7 shows a different recording for the same species which does not exhibit the same high-frequency structure, and which is better-fit by the fractal model.

#### Song tags

Many individual recordings were tagged by the recordist as being songs or calls. Because the scatter across individual recordings is large, we have separated the species into those with such tags and those without them. The species without these musical tags are the Adelie Penguin, Wolves, the Field Cricket, the Lithobates Frog, the Green-Rumped Parrotlet, and the Ryukyu Scops-Owl.

**Figure 8:**
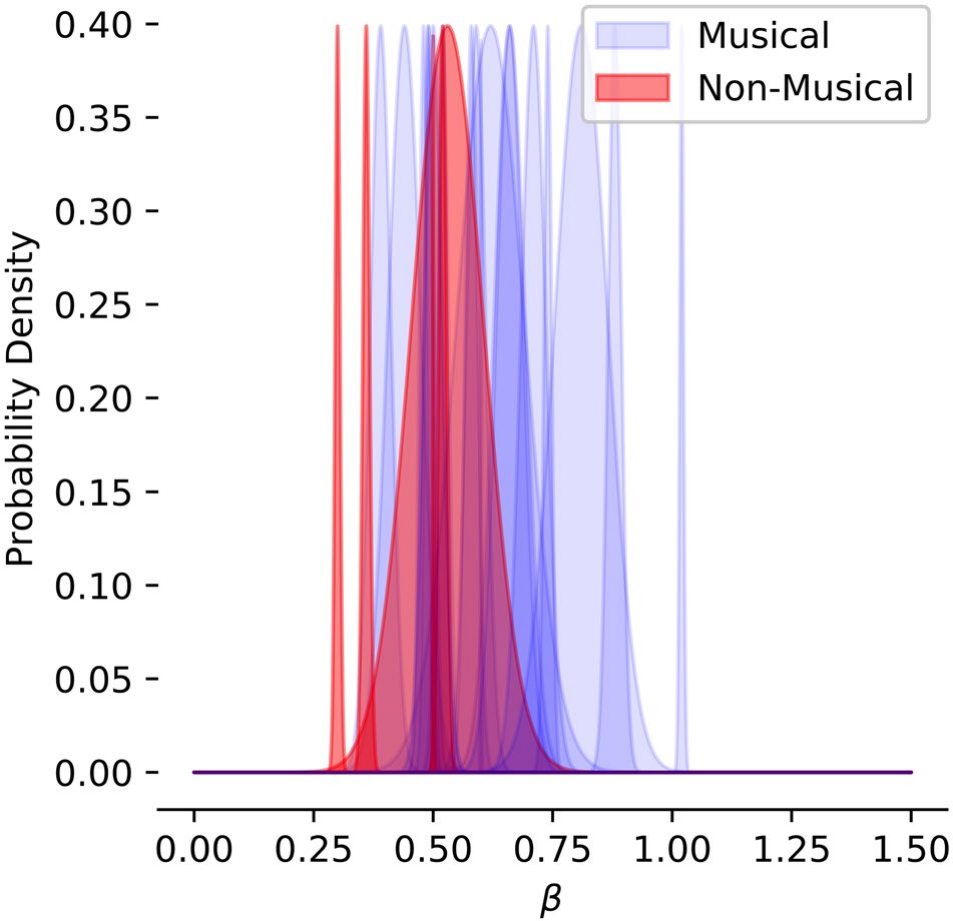
The distribution of spectral indices for each species are shown with blue representing musical species and red representing non-musical ones, according to the criteria laid out in Section III. These distributions are normal with variance and mean set to reflect the population variance and mean as given in SI Table S2.

Our hypothesis in tracking this division was that those species with musical tags would have spectral indices closer to those found in human music, for which *β* ranges from 0*.*4 to 1*.*1 with a preference for higher values^9^. The results are shown in Fig. 8. There is a definite preference in this direction, with the non-musical population generally falling below the bulk of the musical one, but it is not a large preference, and the populations are not cleanly separated by this criterion. Several species were tagged as being musical despite having lower spectral indices than the non-musical ones. This suggests that the spectral index may play a role in whether or not humans identify a recording as musical, with the caveat that there are likely other factors at work.

## Discussion

The effort to discover a formalized method to quantify and relate the complex acoustic sounds produced across the animal kingdom was advanced by Kello et al.^28^ in their study of Allan Factor (AF) variance in which they suggest that 1/*f* correlations may well apply. We are pleased to take up their challenge and demonstrate here the ability of 1/*f* to bind together all these different animal sounds.

The slope *β* reflects the extent to which a signal autocorrelates over time with previously observed values across a wide range of domains conforming to 0.5 ≤ *β* < 1.5. Our findings of intermediate *β* in the vocalizations of non-human species therefore indicate that they encode information in patterns and structures at every timescale.

The patterns indicated may be used as metadata to guide decoding. This is in keeping with the hierarchy of compositional structures humans use, such as notes, chords, verses, and songs, in increasing order of timescale. It has been proposed that such a structure necessarily emerges to ensure that music remains understandable even when notes are shifted in pitch^33^, which potentially reflects the imperfect nature of vocalization.

The particular range of *β* values we obtained also characterizes coastlines^12^, various thermodynamic phase transitions^34^, and various physical noise sources^10^. Given the infinite range of *β* values available, the close coincidence of these spectral exponents with the ranges of human and animal music suggests that this represents the optimal amount of structure for communication and structured encoding as it has evolved in the organisms studied. This is particularly remarkable given that music in humans has become a deliberate, composed act, rather than the hard-wired one it is found to be in some non-human species including songbirds^35^.

Beyond the qualitative structure they indicate, the ranges of *β* found for animals and humans suggest that long time- scale correlations are more frequent in human music than in non-human music, and that these in turn are slightly more frequent than in other vocalizations by non-human species. The similarity and overlap of these distributions are quite striking given the tremendous variety of vocal apparatus represented, indicating that gross physiological structures are unlikely to be responsible for the observed frequency distribution. Of course, while striking, it is not tremendously surprising that the various audio apparatus employed should not have substantial influence on the observed spectra in the ranges studied, as we have analyzed a regime very far from that of tone and perceived sound.

It has been suggested that 1/*f* laws in human music are a result of balancing surprise against predictability^36–40^. While this is no doubt part of the story, its basis is in the finding that human perception and neural encodings are sensitive to 1*/f *^*β*^ distributions^15,16,41–45^, and human neural firing patterns also conform to 1/*f* structure. This is corroborated by studies finding that humans perform many tasks in accordance with 1/*f* laws, including lexical decision, mental rotation, and visual search^46–49^. All told, this points to a more fundamental proclivity towards 1/*f* patterns in our sensory and neural systems^9^. Our finding of 1/*f* structure in non-human music points to an evolutionary origin of this capacity, alongside the evolved sensitivity to the harmonic series, another regularity of the physical world^50,51^.

Adaptive fitness entails that sensory and neural systems should encode regularities in the physical world for an organism to make accurate predictions about its environment. Because there exist temporal and spatial autocorrelations following 1/*f* laws in nature^1,2,12^, it follows that organisms would have evolved mechanisms to detect and ultimately produce them in species-specific calls and vocalizations meant for intra- and inter-species communication.

A further implication of these findings arises in the field of procedural music generation. Music which humans enjoy has been procedurally generated in a stochastic fashion with 1/*f* spectra^8^. With appropriate modifications to use a lower spectral exponent, and to have a familiar pitch structure, it is possible that this technique may be adapted to produce music recognizable to other species.

Here, we analyzed the autocorrelative nature of fluctuations in loudness of the signal and modulation among tones. Recently, multifractal analysis has been applied to model rhythmic expressivity in birdsong of a single thrush nightingale (*Luscinia luscinia*), and the application of this to other animal and human vocalizations is an area for future research^52^.

Contrary to these conclusions it could be argued that the matter which makes up the brain is constrained by the same laws of physics as the matter which makes up a coastline, and hence fractal laws are just a product of those constraints. A similar line of reasoning would suggest that the similarities of vocalizations between human and non- human animals are just a result of similar physical constraints acting upon their means of processing information and producing vocalizations. Such a view is, however, deeply problematic.

To take an extreme case, there are many differences in context between coastlines and brains. For instance, the brain is connected in three-dimensions, whereas a coastline is a boundary between two-dimensional domains. Where fractal-type correlations are concerned dimensionality is crucially important. Similarly, coastlines are constrained by hydrodynamics, the material properties of rocks, the weather, and other such factors, whereas the brain is further subject to electrical, chemical, anatomical, physiological, and evolutionary forces, not to mention the hydrodynamics of blood flow. Hence it is unlikely that the same physical constraints are responsible for these patterns given the vastly different physics at work.

Indeed, a great many phenomena in the natural world do not yield power laws. Correlations of both magnetic spins and solid-matter vibrational modes decay exponentially in all but the most finely tuned situations. Likewise, fundamental modes of resonant cavities exhibit peaked spectra. It therefore cannot be that there is something universal in nature which causes all processes to generate power-law spectra.

Rather, the appearance of non-power law features in nature relates to our point about why the brain ought to have evolved to pick up on power laws. The features of nature which creatures interact with on a regular basis tend to be power-law, while the non-power law features tend to be on very different scales. Coastlines follow power laws, but atomic correlations don't, and the former are more relevant for evolution than the latter. Similarly fundamental modes of resonant cavities are rarely salient while correlations in fish school locations are.

Hence, we arrive at the conclusion that there is something special about the fact that humans and non-human species alike have evolved to produce vocalizations, including music, which reflect power-law fractal structures above all else. A common evolutionary pressure to communicate and encode salient features in the natural world seems the most likely explanation, though there is still much to be done to answer these questions in full.

## Methods

To investigate these phenomena in non-human species we obtained recordings of animal vocalizations (“music” or “animal songs”^53,54^) and other auditorily transmitted communications (e.g. cricket chirps) for 17 species. Audio signals were downloaded from the Macaulay library at the Cornell Laboratory of Ornithology in the wave file format. The individual recordings are listed in Supplementary Information Table S1. A key criterion for selecting species to investigate was the number of recordings of sufficient length for our analysis. We adopted a minimum length criterion of 100 s to have at least two decades of frequency information below the pitch domain of all included species. In addition, for comparison with prior work, we included two recordings of Bach's Brandenburg Concerto #1^55,56^, which hereinafter we attribute to the species “Bach”. We acknowledge the controversy over whether animal sounds such as those analyzed here would be properly classified as speech or as music^57,58^. Our use of Bach in this analysis is for illustrative purposes only, and not intended to represent 1/*f* structure in all of music. We also acknowledge that for those who consider animal sounds "speech," a fairer comparison might have used human songs (music with lyrics) rather than human instrumental music; this comparison has been made elsewhere, with both showing 1*/f* in the same ranges we find here^59^. In total, 1000–30,000 s of recordings were used for each of 17 species, with individual recordings ranging in length from 100 to  2000 s.

The Macaulay library classifies recordings by a variety of standardized tags, generally placed by the recordist. Among these tags are “song” and “call”. To select recordings reflecting animal music, preference was given to species having a large number of recordings with these tags. In selecting individual recordings, preference was given to those having these tags.

We additionally obtained recordings for species which lacked these tags. This allows us to separately analyze those vocalizations which humans subjectively view as musical or not. Notably the distinction was made by the recordist, not by the authors, and so cannot be biased by subsequent analysis.

The Macaulay library includes a spoken description at the start of each recording. The first five seconds of each recording were removed from our sample to avoid including these in our analysis. The volumes in the audio files were then squared. They were subsequently downsampled with three successive eighth-order Chebyshev filters, two with downsample factors of 10 followed by one with a downsample factor of 2. The original signals were recorded at 44,000 Hz so the resulting signals are sampled at 220 Hz. By the Nyquist-Shannon theorem the final signal contains useful information up to 110 Hz^60^. This is a well-established simplifying strategy^8^, given that the spectrum in the pitch domain just reflects the mechanical apparatus used to generate sound, whereas it is the low-frequency modulation of that spectrum that is of interest here. This reflects our assumption that information is primarily encoded in the amplitude modulation, not in the amplitude itself, in the same way that the information in human music is generally in the form of sequences of notes rather than high-frequency details of the notes.

The Fourier Transform of this was then computed. Data above 10 Hz were dropped to further avoid including tonal effects. The remaining data include information about both the loudness of the signal and the modulation between different tones, even though the notes themselves are lost.

Data below 0*.*01 Hz were dropped because not all recordings contained frequency information below this cutoff. In particular, the longest available recordings through Macaulay were roughly two hours long, corresponding to a lower frequency of 0*.*001 Hz, and most species did not have such long recordings available. Many recordings with lengths from ten minutes and upwards were available, however, and so we chose a lower frequency cutoff corresponding to this length.

An example of a spectrum for which no down-sampling was performed is shown in Supplementary Information Fig. S1. Above 30 Hz jagged peaks are evident, indicating resonances in the generating apparatus. Only at lower frequencies does the profile exhibit clear trends.

Bayesian analysis was then performed on the resulting spectrum using two models, an autoregressive moving average model (ARMA), widely used for forecasting^61^, and exhibiting short-term correlations, and a fractal model with long-term correlations^32^. The ARMA model is parameterized as a spectrum of the form

12 $$\left| {\tilde{s}\left( f \right)} \right|^{2} = A\frac{{1 + 2\theta \cos \left( {2\pi f\tau } \right) + \theta^{2} }}{{1 - 2\theta \cos \left( {2\pi f\tau } \right) + \phi^{2} }}$$

where *A* is the amplitude, *f* is the frequency, *τ* is the model timescale, *φ ∈ *[0*,* 1] controls the memory of the model and *θ* is a real-valued parameter controlling the relative strength of the oscillatory component. We take a log-uniform Bayesian prior over *A* which contains the maximum likelihood point for all recordings analyzed, a uniform prior for *φ* over its range, and a uniform prior for *θ* over [*− *2*,* 2]. This last choice is based on precedent^32^. The precise range for this parameter does not matter for any of the recordings we have analyzed. We restrict *τ* to the range [0 s*,* 100 s], corresponding to frequencies above 0*.*01 Hz, so that whatever process is responsible for generating the signal acts at least that fast. This window covers the full range of frequencies present in our recordings and so offers the model maximal freedom.

By comparison the fractal model is of the form

13 $$\left| {\tilde{s}\left( f \right)} \right|^{2} = Af^{ - \beta } + \gamma^{2}$$

where *A* is the amplitude, *γ* produces a cutoff at the high-frequency end and *β* is the spectral index. We take a log-uniform prior over *A*, a uniform prior over [0*,* 10] for *γ* and a uniform prior over [0*,* 4] for *β*. As in the case of *θ*, the Bayesian prior ranges for *γ* and *β* are somewhat arbitrary, but the edges of these ranges are not favored by the posterior distribution and so the interior likely contains the most relevant parts of parameter space.

The inference was performed using the Multinest algorithm in the PyMultinest package^21^. The result is the posterior distribution over model parameters as well as the likelihood of the model. The reported values for each parameter reflect the median and 68% (e.g., one-sigma) confidence intervals after marginalizing over all other parameters.

The likelihood function was chosen to be a normal distribution centered about the measured spectrum with variance equal to *κA* for some constant *κ* which was fit using the same prior as *A*. This form was chosen because it uses a well-known distribution and because when *κ* = 1 it is the large-sample limit of a Poisson distribution, which is a plausible model for the noise in this case.

All code and processed spectra are available at github.com/adamjermyn/Shamu.

## Supplementary Information

Supplementary Information.

## Data Availability

The data that support the findings of this study were downloaded from the Macaulay Library at the Cornell Laboratory of Ornithology (https://www.macaulaylibrary.org/) in the wave file format. The individual recordings are listed in Supplementary Information Table S1.
