# Supplementary material for: 1/f laws found in non-human music
Source: Sci Rep. 2023 Jan 24;13:1324. doi: 10.1038/s41598-023-28444-z (PMC9873655; doi:10.1038/s41598-023-28444-z)
Supplement: Supplementary file 1 — Supplementary Information. [file 41598_2023_28444_MOESM1_ESM.pdf]

1/*f* laws found in non-human music
Supplementary Information

Adam S. Jermyn, David J. Stevenson, Daniel J. Levitin

October 18, 2022

The recordings used in this work are listed in Table S1. The recordings with 24-bit encodings were converted with the open-source Sox software package into a 16-bit encoding. The Python programming language [1] was used along with the NumPy [2] and SciPy [3] packages to parse these 7 files and perform the analysis. Matplotlib [4] was used along with the plotting style ggplot to produce the figures shown.

Table S1: List of recordings used, along with recordists and year and month of recording

ML Catalog #	Recordist	Year	Month
110847	Perkins, Paul J;Winn, Howard E	1973	2
118144	Steiner,WilliamW; Hays, Herbert E	1978	2
117297	Thompson, Thomas J	1978	4
116382	Schassburger, Ronald	1974	6
116383	Schassburger, Ronald	1974	6
116310	Schassburger, Ronald	1972	3
116309	Schassburger, Ronald	1972	3
56761	Gunn, William W. H.	1962	9
116387	Schassburger, Ronald	1974	8
116373	Schassburger, Ronald	1974	6
116385	Schassburger, Ronald	1974	7
16980	Worden, K. W.	1966	6
49615	Little, Randolph S.	1987	6
12481	Allen, Arthur A; Kellogg, Peter Paul	1952	5
195752	McGuire, Bob	2014	5
192119	McGuire, Bob	2014	5
192118	McGuire, Bob	2014	5
110227	Hershberger,Wilbur L	2001	5
105642	Keller, Geoffrey A	1995	5
105640	Keller, Geoffrey A	1995	5
105630	Keller, Geoffrey A	1995	5
105627	Keller, Geoffrey A	1995	5
105614	Keller, Geoffrey A	1995	5
191165	Hershberger,Wilbur L	2012	6
176244	Keller, Geoffrey A	2009	7
134151	Vyn, Gerrit	2007	3
107306	Hershberger,Wilbur L	2000	4
107279	Hershberger,Wilbur L	2000	3
107278	Hershberger,Wilbur L	2000	3
100794	Hershberger,Wilbur L	1999	5
100765	Hershberger,Wilbur L	1999	4
100764	Hershberger,Wilbur L	1999	4

Table S1: List of recordings used, along with recordists and year and month of recording

ML Catalog #	Recordist	Year	Month
100747	Hershberger,Wilbur L	1999	4
100744	Hershberger,Wilbur L	1999	4
98872	Keller, Geoffrey A	1992	4
94288	Hershberger,Wilbur L	1998	4
94283	Hershberger,Wilbur L	1998	4
84706	Hershberger,Wilbur L	1997	2
84683	Hershberger,Wilbur L	1997	2
57975	Gunn, William W. H.	1979	4
49063	Budney, Gregory F	1991	3
197002	McGuire, Bob	2011	3
166628	Bethel, Robert A	2011	6
118628	Keller, Geoffrey A	2001	4
118613	Keller, Geoffrey A	2001	4
100752	Hershberger,Wilbur L	1999	4
94375	Hershberger,Wilbur L	1998	5
94374	Hershberger,Wilbur L	1998	5
94373	Hershberger,Wilbur L	1998	5
85198	Hershberger,Wilbur L	1997	7
50223	Keller, Geoffrey A	1990	6
23403	Zimmerman, Dale A	1980	6
94327	Hershberger,Wilbur L	1998	5
192210	McGowan, Jay W	2014	6
191267	Little, Randolph S	2003	5
191158	Hershberger,Wilbur L	2012	5
176167	Keller, Geoffrey A	2009	5
125225	Andersen,Michael J	2005	5
84866	Hershberger,Wilbur L	1997	5
84865	Hershberger,Wilbur L	1997	5
84864	Hershberger,Wilbur L	1997	5
59213	Gunn,WilliamW. H.	1976	5
59210	Gunn,WilliamW. H.	1958	7
59206	Gunn,WilliamW. H.	1956	6
50268	Keller, Geoffrey A	1990	6
113501	Marantz, Curtis A	1998	5
113500	Marantz, Curtis A	1998	5
112699	Marantz, Curtis A	1998	5
112697	Marantz, Curtis A	1998	5
94326	Hershberger,Wilbur L	1998	5
12483	Allen, Arthur A; Kellogg, Peter Paul	1952	6
12482	Allen, Arthur A; Kellogg, Peter Paul	1952	6
164538	Fischer,Martha J	2011	6
163341	Medler,Matthew D	2011	5
112698	Marantz, Curtis A	1998	5
176241	Keller, Geoffrey A	2009	7
130905	Vyn, Gerrit	2006	3

Table S1: List of recordings used, along with recordists and year and month of recording

ML Catalog #	Recordist	Year	Month
94372	Hershberger,Wilbur L	1998	5
85197	Hershberger,Wilbur L	1997	7
85196	Hershberger,Wilbur L	1997	7
85193	Hershberger,Wilbur L	1997	7
85192	Hershberger,Wilbur L	1997	7
56847	Keller, Geoffrey A	1991	5
56847	Keller, Geoffrey A	1991	5
23402	Zimmerman, Dale A	1980	5
22933	Fish,William R	1953	6
22932	Fish,William R	1953	6
113502	Marantz, Curtis A	1998	5
94313	Hershberger,Wilbur L	1998	5
53163	Pantle, Steven R	1991	5
107970	Pantle, Steven R	1992	5
74906	Marantz, Curtis A	1994	5
94325	Hershberger,Wilbur L	1998	5
176144	Keller, Geoffrey A	2007	7
176115	Keller, Geoffrey A	2007	5
4221	Allen, Arthur A; Kellogg, Peter Paul	1951	6
179528	McCartt, David A	2008	6
171721	Areta, Juan I	2012	11
136190	Medler,Matthew D	2007	6
136156	Medler,Matthew D	2007	6
131468	Vyn, Gerrit	2006	6
130939	Vyn, Gerrit	2006	5
121953	Marantz, Curtis A	2005	6
126471	Sander, Thomas G	1988	5
119446	Keller, Geoffrey A	2001	7
135727	Heckscher, ChristopherM	2006	6
3646	Allen, Arthur A; Kellogg, Peter Paul	1951	5
188826	McGuire, Bob	2013	5
188824	McGuire, Bob	2013	5
136577	O’Brien, Sean	2011	6
67777	Gunn,WilliamW. H.	1957	6
26770	Plymire,Margery R	1979	6
12515	Davis, L. Irby ; Guion,William	1961	6
12519	Thurber,Walter A	1971	8
20382	Thurber,Walter A	1979	5
20792	Thurber,Walter A	1976	2
105591	Keller, Geoffrey A	1995	5
136579	O’Brien, Sean	2011	7
66744	Gunn,WilliamW. H.	1953	6
73979	Keller, Geoffrey A	1992	6
36955	van den Berg, Arnoud B	1986	7
57999	Gunn,WilliamW. H.	1979	6

Table S1: List of recordings used, along with recordists and year and month of recording

ML Catalog #	Recordist	Year	Month
10211	Allen, Arthur A; Kellogg, Peter Paul	1952	5
107339	Hershberger,Wilbur L	2000	5
176248	Keller, Geoffrey A	2009	7
121994	Marantz, Curtis A	2005	7
11434	Parker, III, Theodore A	1977	5
94321	Hershberger,Wilbur L	1998	5
22454	Davis,William E., Jr. E		
176262	Keller, Geoffrey A	2009	7
100754	Hershberger,Wilbur L	1999	4
11180	McChesney,Marian P	1960	4
11175	Allen, Arthur A; Allen, David G	1956	2
60091	Gulledge, James L	1971	3
148978	Sly, Nicholas D	2010	2
94279	Hershberger,Wilbur L	1998	4
84769	Hershberger,Wilbur L	1997	4
135412	Andersen,Michael J	2007	3
128930	Vyn, Gerrit ; Clock, BenjaminM	2005	3
4548	Stein, Robert C; Little, Randolph S	1963	4
110209	Hershberger,Wilbur L	2001	5
52320	Parker, III, Theodore A	1990	5
175898	Budney, Gregory F	1993	5
125364	Sander, Thomas G	1992	5
105433	Keller, Geoffrey A	1994	6
120251	Keller, Geoffrey A	2002	5
197943	McGuire, Bob	2010	3
148337	Clock, BenjaminM	2006	5
63219	Gunn,WilliamW. H.	1978	4
45080	Keller, Geoffrey A	1985	6
9044	Stein, Robert C; Angstadt, Robert B	1964	4
168331	Hershberger,Wilbur L	2011	6
107333	Hershberger,Wilbur L	2000	5
107324	Hershberger,Wilbur L	2000	5
100890	Hershberger,Wilbur L	1999	6
94417	Hershberger,Wilbur L	1998	6
94416	Hershberger,Wilbur L	1998	6
94312	Hershberger,Wilbur L	1998	5
84903	Hershberger,Wilbur L	1997	6
40807	Budney, Gregory F	1988	5
11346	Kellogg, Peter Paul	1951	6
11342	Stein, Robert C;Morton, Eugene S	1964	5
11317	Allen, Arthur A; Kellogg, Peter Paul	1951	5
11316	Allen, Arthur A; Kellogg, Peter Paul	1951	5
11308	Kellogg, Peter Paul	1951	6
191174	Hershberger,Wilbur L	2012	6
176259	Keller, Geoffrey A	2009	7

Table S1: List of recordings used, along with recordists and year and month of recording

ML Catalog #	Recordist	Year	Month
176204	Keller, Geoffrey A	2009	6
176200	Keller, Geoffrey A	2009	6
133353	Keller, Geoffrey A	2007	7
130994	Vyn, Gerrit	2006	6
119447	Keller, Geoffrey A	2001	7
119444	Keller, Geoffrey A	2001	7
100877	Hershberger,Wilbur L	1999	5
93794	Medler,Matthew D	1998	5
93793	Medler,Matthew D	1998	5
4240	Stein, Robert C; Little, Randolph S	1962	6
4232	Stein, Robert C;Michener,Martin C	1959	6
188871	McGuire, Bob	2013	7
118694	Keller, Geoffrey A	2001	5
135720	Heckscher, ChristopherM	2004	6
121915	Marantz, Curtis A	2003	6
3663	Stein, Robert C; Little, Randolph S	1963	7
3648	Allen, Arthur A; Kellogg, Peter Paul	1951	5
67782	Gunn,WilliamW. H.	1960	6
164574	McGowan, JayW	2011	6
135714	Heckscher, ChristopherM	2004	5
27193	Little, Randolph S	1980	6
146568	Robbins,Mark B	2006	6
197042	McGuire, Bob	2011	5
197036	McGuire, Bob	2011	5
927	Miller, R. S	1967	10
193551	Nelson, Richard	2013	6
72720	Evers, David C	1991	5
61477	Gunn,WilliamW. H.	1969	8
61479	Gunn,WilliamW. H.	1971	6
916	Allen, Arthur A; Allen, David G	1947	6
918	Allen, Arthur A; Allen, David G	1947	6
913	Allen, Arthur A; Kellogg, Peter Paul	1947	6
72736	Evers, David C	1990	5
131218	Andersen,Michael J	2006	9
140215	Vyn, Gerrit	2006	9
66742	Gunn,WilliamW. H.	1951	5
169021	Medler,Matthew D	2012	6
66745	Gunn,WilliamW. H.	1954	6
15586	Allen, Arthur A; Allen, Elsa G	1958	2
107292	Hershberger,Wilbur L	2000	3
66766	Gunn,WilliamW. H.	1960	6
188878	McGuire, Bob	2013	7
84685	Hershberger,Wilbur L	1997	2
169036	Medler,Matthew D	2012	6
15594	Stein, Robert C; Little, Randolph S	1962	6

Table S1: List of recordings used, along with recordists and year and month of recording

ML Catalog #	Recordist	Year	Month
133396	Fischer,Martha J	2007	7
15603	Stein, Robert C; Little, Randolph S	1963	6
15601	Stein, Robert C; Little, Randolph S	1963	6
137668	Medler,Matthew D	2008	7
15562	Allen, Arthur A; Kellogg, Peter Paul	1954	7
15605	Stein, Robert C; Little, Randolph S	1963	6
168306	Hershberger,Wilbur L	2011	6
191237	Hershberger,Wilbur L	2012	7
10218	Kellogg, Peter Paul	1959	6
107340	Hershberger,Wilbur L	2000	5
93763	Medler,Matthew D	1998	5
121989	Marantz, Curtis A	2005	7
50269	Keller, Geoffrey A	1990	6
181512	Budney, Gregory F	2013	6
11174	Hartshorne, JamesM	1955	5
67309	Gunn,WilliamW. H.	1953	6
100774	Hershberger,Wilbur L	1999	4
84768	Hershberger,Wilbur L	1997	4
190985	Little, Randolph S	2013	6
100779	Hershberger,Wilbur L	1999	4
84766	Hershberger,Wilbur L	1997	4
164590	McGowan, JayW	2011	6
67312	Gunn,WilliamW. H.	1958	6
105379	Keller, Geoffrey A	1994	5
176256	Keller, Geoffrey A	2009	7
187078	McGuire, Bob	2013	7
128926	Vyn, Gerrit ; Clock, BenjaminM	2005	3
128925	Vyn, Gerrit ; Clock, BenjaminM	2005	3
4549	Stein, Robert C; Gunn,WilliamW. H.	1963	4
134150	Vyn, Gerrit	2007	3
163907	O'Brien, Sean	2010	4
191186	Hershberger,Wilbur L	2012	6
128933	Vyn, Gerrit ; Clock, BenjaminM	2005	3
63218	Gunn,WilliamW. H.	1970	6
120205	Keller, Geoffrey A	2002	5
120253	Keller, Geoffrey A	2002	5
120253	Keller, Geoffrey A	2002	5
44687	Tucker, Nigel	1988	4
9043	Bartholemy, Richard E	1961	4
63220	Gunn,WilliamW. H.	1981	4
9041	Stein, Robert C; Angstadt, Robert B	1961	4
197170	McGuire, Bob	2010	2
105214	Keller, Geoffrey A	1993	5
110950	Sander, Thomas G	1986	4
197171	McGuire, Bob	2010	2

Table S1: List of recordings used, along with recordists and year and month of recording

ML Catalog #	Recordist	Year	Month
163246	Lasley, GregW	1986	8
44685	Tucker, Nigel	1988	4
189253	McGuire, Bob	2012	3
105240	Keller, Geoffrey A	1993	5
37733	Minis, Dolly	1985	3
148381	Clock, BenjaminM	2006	5
120340	Poulter, Thomas C	1964	12
127112	Budney, Gregory F	1990	2
899	Arnell,Walter	1970	2
141363	Barry, Jessie H	2010	1
42338	Parker, III, Theodore A	1988	2
42334	Parker, III, Theodore A	1988	2
141360	Barry, Jessie H	2010	1
42311	Parker, III, Theodore A	1988	2
42331	Parker, III, Theodore A	1988	2
42313	Parker, III, Theodore A	1988	2
120344	Poulter, Thomas C	1964	12
42337	Parker, III, Theodore A	1988	2
148921	Berg, Karl S	2007	10
148929	Berg, Karl S	2008	7
148944	Berg, Karl S	2008	7
148908	Berg, Karl S	2006	6
148926	Berg, Karl S	2006	7
148941	Berg, Karl S	2007	10
148940	Berg, Karl S	2007	10
148949	Berg, Karl S	2006	7
148952	Berg, Karl S	2006	7
148934	Berg, Karl S	2007	6
148930	Berg, Karl S	2008	7
148913	Berg, Karl S	2006	6
148953	Berg, Karl S	2006	7
64383	Schwartz, Paul A	1961	7
175011	King, Ben F	1983	2
114835	Hsu,WayneW	2001	6
42159	Severinghaus, Lucia L	1986	4
64385	Schwartz, Paul A	1973	2
148911	Berg, Karl S	2006	6
68982	Gunn,WilliamW. H.	1956	1
42130	Severinghaus, Lucia L		
42097	Severinghaus, Lucia L	1985	11
42118	Severinghaus, Lucia L	1986	2
42181	Severinghaus, Lucia L	1986	6
42138	Severinghaus, Lucia L	1986	4
42089	Severinghaus, Lucia L	1985	10
42090	Severinghaus, Lucia L	1985	10

Table S1: List of recordings used, along with recordists and year and month of recording

ML Catalog #	Recordist	Year	Month
42086	Severinghaus, Lucia L	1985	10
42149	Severinghaus, Lucia L	1986	4
42112	Severinghaus, Lucia L	1986	2
42170	Severinghaus, Lucia L	1986	5
42164	Severinghaus, Lucia L	1986	4
42190	Severinghaus, Lucia L	1986	7
175861	McNeill, Roger D	2011	11
42087	Severinghaus, Lucia L	1985	10
42167	Severinghaus, Lucia L	1986	5
42150	Severinghaus, Lucia L	1986	4
117873	Winn, Howard E	1974	3
117872	Perkins, Paul J	1974	3
117860	Perkins, Paul J	1974	2
123414	Harrington, Flip ;Whiting, Susan B	1981	1
118127	Steiner,WilliamW; Hays, Herbert E	1978	1
118125	Steiner,WilliamW; Hays, Herbert E	1978	1
118126	Steiner,WilliamW; Hays, Herbert E	1978	1
62943	Budney, Gregory F	1989	7
191166	Hershberger,Wilbur L	2012	6
176147	Keller, Geoffrey A	2007	7
191199	Hershberger,Wilbur L	2012	6
101800	Hennessey, A. Bennett	1998	10
55546	Kerr, Donald J	1993	7
191222	Hershberger,Wilbur L	2012	7
73930	Keller, Geoffrey A	1992	4
116519	Smith, Shelagh A; Budney, Gregory F	2003	6
41163	Minis, Dolly	1978	6
38530	Kibler, Lewis F	1987	6
176204	Keller, Geoffrey A	2009	6
176281	Keller, Geoffrey A	2010	6
116518	Smith, Shelagh A; Budney, Gregory F	2003	6
188886	McGuire, Bob	2013	7
7392	Stein, Robert C; Little, Randolph S	1963	7
68577	Kerr, Donald J	1994	5
515922	Robbins,Mark B	2014	6
110260	Hershberger,Wilbur L	2001	6
128215	Ljungblad, Donald K	1976	12
128218	Ljungblad, Donald K		
123124	Van Parijs, Sofie ; Van Opzeeland, Ilse C	2002	11
191240	Hershberger,Wilbur L	2012	8
138552	Budney, Gregory F	1996	6
182026	Gerhardt, H. Carl	1971	4
182118	Gerhardt, H. Carl	1972	6
71895	Fischer,Martha J	2005	4
136572	O'Brien, Sean	2011	6

Table S1: List of recordings used, along with recordists and year and month of recording

ML Catalog #	Recordist	Year	Month
179100	Gerhardt, H. Carl	1969	3
163334	Medler,Matthew D	2010	8
163335	Medler,Matthew D	2010	8
128926	Vyn, Gerrit ; Clock, BenjaminM	2005	3
128925	Vyn, Gerrit ; Clock, BenjaminM	2005	3
134150	Vyn, Gerrit	2007	3
128930	Vyn, Gerrit ; Clock, BenjaminM	2005	3
105433	Keller, Geoffrey A	1994	6
201156	McGowan, JayW	2015	4
128924	Vyn, Gerrit ; Clock, BenjaminM	2005	3
128931	Vyn, Gerrit ; Clock, BenjaminM	2005	3
49708	Evans,William R	1989	4
120761	Barlow, Jay	2003	9
123105	Van Parijs, Sofie ; Van Opzeeland, Ilse C	2002	11
128214	Ljungblad, Donald K	1976	12
128216	Ljungblad, Donald K	1976	12
128223	Ljungblad, Donald K	1976	12
128222	Ljungblad, Donald K	1976	12
128217	Ljungblad, Donald K	1976	12
128204	Ljungblad, Donald K	1976	
123145	Van Parijs, Sofie ; Van Opzeeland, Ilse C	2002	11
120595	Van Parijs, Sofie ; Van Opzeeland, Ilse C	2002	11
123132	Van Parijs, Sofie ; Van Opzeeland, Ilse C	2002	11
123136	Van Parijs, Sofie ; Van Opzeeland, Ilse C	2002	11
200846	Weissman, David B	2014	10
54805	Little, Randolph S	2009	9
85189	Hershberger,Wilbur L	1997	6
182023	Gerhardt, H. Carl	1971	7
195216	Nelson, Richard	2013	9
79462	Hershberger,Wilbur L	1996	6
36950	van den Berg, Arnoud B	1986	7
72743	Evers, David C	1989	4
134149	Vyn, Gerrit	2007	3
123142	Van Parijs, Sofie ; Van Opzeeland, Ilse C	2002	11
123125	Van Parijs, Sofie ; Van Opzeeland, Ilse C	2002	11
85185	Hershberger,Wilbur L	1997	6
85328	Hershberger,Wilbur L	1997	10
85329	Hershberger,Wilbur L	1997	10
85187	Hershberger,Wilbur L	1997	6
85154	Hershberger,Wilbur L	1997	6
200359	Weissman, David B	2013	7
200710	Weissman, David B	2013	10
200801	Weissman, David B	2014	8
85186	Hershberger,Wilbur L	1997	6
200796	Weissman, David B	2014	8

Table S1: List of recordings used, along with recordists and year and month of recording

ML Catalog #	Recordist	Year	Month
138394	Budney, Gregory F	1996	4
138553	Budney, Gregory F	1996	6
138550	Budney, Gregory F	1996	6
104393	Budney, Gregory F	2000	5
138551	Budney, Gregory F	1996	6
138373	Budney, Gregory F	1996	4
130487	Budney, Gregory F	1997	4

Table S2: For each species the difference in logarithmic evidence (log Z), weighted mean (μ_β), standard deviation thereof (σ_β) and population standard deviation are shown. The logarithmic evidence is summed across all recordings for each species. Note that in all cases the fractal model is strongly favoured over the ARMA one. The population standard deviation is typically larger than that for the mean because the mean of a sample varies less than the individual members. Weights were obtained from the Bayesian posterior distribution for each recording.

Species	$\log Z_{ARMA} - \log Z_{Fractal}$	μ_β	$\sigma_{\beta,\mu}$	$\sigma_{\beta,pop}$
Adelie Penguin	-26657	0.52	0.002	0.008
Altamira Oriole	-6010.66	0.81	0.003	0.056
Bach	-17792.3	0.65	0.001	0
Baltimore Oriole	-8196.58	0.49	0.002	0.012
Barred Owl	-74238	0.88	0	0.014
Brown Thrasher	-32024.4	0.62	0.001	0.071
Canyon Wren	-21461	0.59	0.003	0.02
Common Loon	-40789.5	1.02	0	0.004
Eastern Wood-Pewee	-34020.9	0.6	0	0.001
Field Cricket	-1340.31	0.36	0.003	0.007
Frog	-29441.4	0.3	0.001	0.005
Gray Catbird	-22857.4	0.39	0.001	0.022
Green-Rumped Parrotlet	-11975.1	0.53	0.003	0.075
Humpback Whale	-117581	0.66	0.001	0.028
Killer Whale	-71010.6	0.74	0	0.008
Northern Cardinal	-11253.1	0.5	0.002	0.019
Northern Mockingbird	-15378.5	0.44	0.002	0.035
Rose-breasted Grosbeak	-13888	0.58	0.001	0.013
Ryukyu Scops Owl	-34183	0.48	0.001	0.009
Swainson’s Thrush	-20580.1	0.66	0.001	0.033
Veery	-35097.9	0.71	0.001	0.026
White-throated Sparrow	-28366.8	0.52	0.001	0.012
Wolves	-26604.6	0.5	0	0.001
Wood Thrush	-21355.3	0.49	0.001	0.008

Table S3: Evidence for each model and recording

Common Name	Species	Recording ID	$logZ_{ARMA}$	$logZ_{Fractal}$
Adelie Penguin	Pygoscelis Adeliae	247294	-238966.517	-237050.953
Adelie Penguin	Pygoscelis Adeliae	247293	-281911.596	-278389.491
Adelie Penguin	Pygoscelis Adeliae	247295	-175554.757	-170949.296
Adelie Penguin	Pygoscelis Adeliae	247301	-92960.548	-91671.486
Adelie Penguin	Pygoscelis Adeliae	247300	-71134.679	-68447.321
Adelie Penguin	Pygoscelis Adeliae	247297	-110438.777	-108235.402
Adelie Penguin	Pygoscelis Adeliae	247299	-84526.462	-82807.437
Adelie Penguin	Pygoscelis Adeliae	247298	-149903.950	-148193.431
Adelie Penguin	Pygoscelis Adeliae	247296	-256504.113	-253534.025
Adelie Penguin	Pygoscelis Adeliae	247302	-55172.899	-53733.217
Adelie Penguin	Pygoscelis Adeliae	247303	-48881.131	-47155.463
Adelie Penguin	Pygoscelis Adeliae	247304	-47040.973	-46171.924
Altamira Oriole	Icterus Gularis	12515	-70038.616	-67111.237
Altamira Oriole	Icterus Gularis	105591	-25219.254	-24992.848
Altamira Oriole	Icterus Gularis	20382	-54652.595	-52809.689
Altamira Oriole	Icterus Gularis	140215	-22705.824	-22695.600
Altamira Oriole	Icterus Gularis	20792	-29361.074	-28848.942
Altamira Oriole	Icterus Gularis	12519	-64058.008	-63488.741
Altamira Oriole	Icterus Gularis	131218	-29170.453	-29248.112
Bach - Brandenburg Concerto 1 long			-402785.987	-398104.361
Bach - Brandenburg Concerto 1 long2			-501761.701	-488651.052
Baltimore Oriole	Icterus Galbula	105614	-27788.389	-27000.344
Baltimore Oriole	Icterus Galbula	105640	-33777.207	-33762.946
Baltimore Oriole	Icterus Galbula	12481	-41509.538	-40941.381
Baltimore Oriole	Icterus Galbula	164538	-47900.369	-47819.414
Baltimore Oriole	Icterus Galbula	163341	-44339.520	-44414.133
Baltimore Oriole	Icterus Galbula	94326	-36170.532	-35494.389
Baltimore Oriole	Icterus Galbula	195752	-37502.312	-37477.930
Baltimore Oriole	Icterus Galbula	105630	-28436.585	-28005.187
Baltimore Oriole	Icterus Galbula	105642	-15151.076	-14925.395
Baltimore Oriole	Icterus Galbula	112699	-15865.966	-15522.533
Baltimore Oriole	Icterus Galbula	112697	-24215.906	-24226.322
Baltimore Oriole	Icterus Galbula	105627	-25689.236	-25425.684
Baltimore Oriole	Icterus Galbula	112698	-12063.583	-11607.617
Baltimore Oriole	Icterus Galbula	12482	-503.270	-494.811
Baltimore Oriole	Icterus Galbula	12483	-75277.646	-73696.085
Baltimore Oriole	Icterus Galbula	110227	-42663.041	-41207.718
Baltimore Oriole	Icterus Galbula	192118	-129491.054	-129275.900
Baltimore Oriole	Icterus Galbula	113500	-56436.351	-55516.964
Baltimore Oriole	Icterus Galbula	113501	-11918.545	-11839.857
Baltimore Oriole	Icterus Galbula	192119	-50295.889	-50144.827
Barred Owl	Strix Varia	134150	-291901.225	-289050.144
Barred Owl	Strix Varia	135412	-297140.426	-292270.802
Barred Owl	Strix Varia	187078	-1008047.650	-994264.286
Barred Owl	Strix Varia	128933	-140996.463	-138673.411

Table S3: Evidence for each model and recording

Common Name	Species	Recording ID	$logZ_{ARMA}$	$logZ_{Fractal}$
Barred Owl	Strix Varia	79462	-50390.975	-49905.312
Barred Owl	Strix Varia	125364	-122699.609	-121368.452
Barred Owl	Strix Varia	49708	-52309.125	-50772.755
Barred Owl	Strix Varia	4549	-161584.606	-156969.054
Barred Owl	Strix Varia	128926	-388085.681	-384262.993
Barred Owl	Strix Varia	4548	-139784.448	-137116.438
Barred Owl	Strix Varia	128931	-78631.576	-78046.951
Barred Owl	Strix Varia	191186	-164322.624	-159079.253
Barred Owl	Strix Varia	128930	-281556.579	-275955.062
Barred Owl	Strix Varia	36950	-49731.769	-48253.890
Barred Owl	Strix Varia	110209	-150517.665	-148075.729
Barred Owl	Strix Varia	195216	-141274.968	-140559.884
Barred Owl	Strix Varia	175898	-128215.900	-125210.808
Barred Owl	Strix Varia	163907	-109179.509	-105677.610
Barred Owl	Strix Varia	201156	-101781.670	-100876.707
Barred Owl	Strix Varia	105433	-83923.254	-82913.701
Barred Owl	Strix Varia	72743	-46743.028	-45029.807
Barred Owl	Strix Varia	128924	-95976.277	-94396.305
Barred Owl	Strix Varia	134149	-73864.664	-73157.215
Barred Owl	Strix Varia	52320	-130988.903	-127816.500
Barred Owl	Strix Varia	128925	-307603.873	-303311.353
Brown Thrasher	Toxostoma Rufum	67309	-94451.925	-93757.841
Brown Thrasher	Toxostoma Rufum	148978	-88847.781	-88706.142
Brown Thrasher	Toxostoma Rufum	100774	-101933.397	-99827.075
Brown Thrasher	Toxostoma Rufum	67312	-91956.421	-90764.940
Brown Thrasher	Toxostoma Rufum	190985	-99143.856	-98665.907
Brown Thrasher	Toxostoma Rufum	164590	-93982.215	-93119.863
Brown Thrasher	Toxostoma Rufum	176256	-87550.564	-86137.573
Brown Thrasher	Toxostoma Rufum	11174	-116515.728	-115315.277
Brown Thrasher	Toxostoma Rufum	105379	-89768.220	-88958.327
Brown Thrasher	Toxostoma Rufum	11180	-149779.033	-147346.484
Brown Thrasher	Toxostoma Rufum	84769	-84453.486	-82087.472
Brown Thrasher	Toxostoma Rufum	11175	-118309.880	-117318.832
Brown Thrasher	Toxostoma Rufum	84766	-98160.720	-97203.076
Brown Thrasher	Toxostoma Rufum	84768	-97368.402	-95996.835
Brown Thrasher	Toxostoma Rufum	94279	-91515.097	-90237.772
Brown Thrasher	Toxostoma Rufum	100779	-100820.268	-99869.361
Brown Thrasher	Toxostoma Rufum	60091	-108442.363	-106262.897
Brown Thrasher	Toxostoma Rufum	181512	-255622.533	-254273.209
Brown Thrasher	Toxostoma Rufum	100754	-178792.012	-176269.723
Brown Thrasher	Toxostoma Rufum	176262	-318967.331	-312238.224
Canyon Wren	Catherpes Mexicanus	197170	-72233.668	-72287.763
Canyon Wren	Catherpes Mexicanus	120253	-81654.717	-81030.618
Canyon Wren	Catherpes Mexicanus	197171	-54541.593	-54437.898
Canyon Wren	Catherpes Mexicanus	197943	-217203.670	-215905.912

Table S3: Evidence for each model and recording

Common Name	Species	Recording ID	logZ$_{ARMA}$	logZ$_{Fractal}$
Canyon Wren	CatherpesMexicanus	44687	-102551.231	-102684.108
Canyon Wren	Catherpes Mexicanus	105214	-43505.532	-43228.086
Canyon Wren	Catherpes Mexicanus	9043	-61235.373	-60346.890
Canyon Wren	Catherpes Mexicanus	9044	-22612.017	-22634.494
Canyon Wren	Catherpes Mexicanus	37733	-17382.610	-17252.032
Canyon Wren	Catherpes Mexicanus	105240	-19043.716	-18514.277
Canyon Wren	Catherpes Mexicanus	110950	-42926.637	-41841.728
Canyon Wren	Catherpes Mexicanus	148337	-79862.218	-77741.447
Canyon Wren	Catherpes Mexicanus	189253	-32338.264	-31932.704
Canyon Wren	Catherpes Mexicanus	148381	-70835.225	-70908.253
Canyon Wren	Catherpes Mexicanus	120251	-137668.658	-136223.753
Canyon Wren	Catherpes Mexicanus	45080	-29377.356	-28331.424
Canyon Wren	Catherpes Mexicanus	120205	-133347.838	-129641.568
Canyon Wren	Catherpes Mexicanus	44685	-34837.895	-34872.004
Canyon Wren	Catherpes Mexicanus	163246	-25170.107	-25013.507
Canyon Wren	Catherpes Mexicanus	63220	-55761.774	-53955.752
Canyon Wren	Catherpes Mexicanus	63218	-123235.277	-119992.564
Canyon Wren	Catherpes Mexicanus	9041	-56520.395	-54744.050
Canyon Wren	Catherpes Mexicanus	63219	-41825.089	-40689.029
Common Loon	Gavia Immer	197042	-351890.127	-347279.889
Common Loon	Gavia Immer	72736	-145562.410	-141137.272
Common Loon	Gavia Immer	913	-107942.724	-105103.248
Common Loon	Gavia Immer	61479	-106779.692	-102015.497
Common Loon	Gavia Immer	61477	-80565.983	-77982.617
Common Loon	Gavia Immer	193551	-195284.659	-194738.987
Common Loon	Gavia Immer	72720	-131138.588	-125234.478
Common Loon	Gavia Immer	197036	-553807.580	-548531.684
Common Loon	Gavia Immer	927	-256768.476	-250050.678
Common Loon	Gavia Immer	918	-103985.854	-101966.562
Common Loon	Gavia Immer	916	-75529.605	-74425.286
EasternWood-Pewee	Contopus Virens	73930	-81367.729	-77356.097
EasternWood-Pewee	Contopus Virens	176147	-123125.492	-119877.560
EasternWood-Pewee	Contopus Virens	55546	-93301.956	-90920.715
EasternWood-Pewee	Contopus Virens	41163	-72894.411	-71954.872
EasternWood-Pewee	Contopus Virens	110260	-48573.961	-47234.509
EasternWood-Pewee	Contopus Virens	191222	-151054.054	-150306.096
EasternWood-Pewee	Contopus Virens	188886	-98570.594	-97262.770
EasternWood-Pewee	Contopus Virens	191199	-187110.135	-186722.972
EasternWood-Pewee	Contopus Virens	7392	-60418.072	-59146.486
EasternWood-Pewee	Contopus Virens	68577	-55850.573	-54498.567
EasternWood-Pewee	Contopus Virens	116518	-95353.763	-95132.414
EasternWood-Pewee	Contopus Virens	176204	-68453.574	-67260.991
EasternWood-Pewee	Contopus Virens	116519	-124022.174	-123107.247
EasternWood-Pewee	Contopus Virens	176281	-64748.088	-63993.884
EasternWood-Pewee	Contopus Virens	62943	-242438.657	-239284.334

Table S3: Evidence for each model and recording

Common Name	Species	Recording ID	$logZ_{ARMA}$	$logZ_{Fractal}$
EasternWood-Pewee	Contopus Virens	38530	-67640.180	-66061.896
EasternWood-Pewee	Contopus Virens	101800	-105643.211	-103234.997
EasternWood-Pewee	Contopus Virens	191166	-214870.020	-208059.376
Field Cricket	Gryllus	200359	-28344.877	-28380.740
Field Cricket	Gryllus	85154	-29558.992	-29931.070
Field Cricket	Gryllus	54805	-41586.752	-40614.398
Field Cricket	Gryllus	200846	-51870.499	-51483.721
Field Cricket	Gryllus	191240	-69010.898	-69557.442
Field Cricket	Gryllus	200801	-26335.339	-26180.999
Field Cricket	Gryllus	200710	-26970.366	-26264.830
Field Cricket	Gryllus	85329	-51533.726	-51585.809
Field Cricket	Gryllus	85185	-79556.884	-79088.072
Field Cricket	Gryllus	85328	-61122.574	-61469.767
Field Cricket	Gryllus	200796	-23961.026	-23776.439
Field Cricket	Gryllus	85187	-30362.082	-30391.952
Field Cricket	Gryllus	85189	-40799.997	-40908.136
Field Cricket	Gryllus	85186	-25858.189	-25898.513
Frog	Lithobates	71895	-214781.886	-214894.392
Frog	Lithobates	182118	-163565.828	-157906.823
Frog	Lithobates	163335	-105307.204	-104539.009
Frog	Lithobates	163334	-114026.075	-113186.976
Frog	Lithobates	182023	-71164.606	-69478.732
Frog	Lithobates	179100	-112402.704	-112038.727
Frog	Lithobates	138552	-384856.872	-382214.059
Frog	Lithobates	138394	-2789558.230	-2786210.090
Frog	Lithobates	138553	-1997771.139	-1987418.315
Frog	Lithobates	182026	-166834.275	-164466.175
Frog	Lithobates	136572	-144130.002	-142604.080
Gray Catbird	Dumetella Carolinensis	57999	-139678.522	-138243.696
Gray Catbird	Dumetella Carolinensis	107339	-141421.264	-139831.217
Gray Catbird	Dumetella Carolinensis	36955	-172163.815	-170985.443
Gray Catbird	Dumetella Carolinensis	11434	-91029.911	-89034.896
Gray Catbird	Dumetella Carolinensis	121989	-163993.041	-163256.059
Gray Catbird	Dumetella Carolinensis	22454	-89903.786	-88775.618
Gray Catbird	Dumetella Carolinensis	168306	-1208801.127	-1202622.883
Gray Catbird	Dumetella Carolinensis	121994	-105043.735	-103879.936
Gray Catbird	Dumetella Carolinensis	107340	-207621.121	-206747.980
Gray Catbird	Dumetella Carolinensis	176248	-119749.184	-118159.086
Gray Catbird	Dumetella Carolinensis	50269	-96758.728	-96146.928
Gray Catbird	Dumetella Carolinensis	191237	-451428.095	-449737.947
Gray Catbird	Dumetella Carolinensis	10211	-149091.951	-149107.668
Gray Catbird	Dumetella Carolinensis	10218	-239265.911	-238226.655
Gray Catbird	Dumetella Carolinensis	94321	-85907.353	-85314.240
Gray Catbird	Dumetella Carolinensis	93763	-182340.658	-181270.525
Green-Rumped Parrotlet	Forpus Passerinus	247305	-149968.851	-147991.613

Table S3: Evidence for each model and recording

Common Name	Species	Recording ID	$logZ_{ARMA}$	$logZ_{Fractal}$
Green-Rumped Parrotlet	Forpus Passerinus	247321	-46445.032	-46284.765
Green-Rumped Parrotlet	Forpus Passerinus	247319	-44757.318	-44226.242
Green-Rumped Parrotlet	Forpus Passerinus	247310	-66866.436	-65731.801
Green-Rumped Parrotlet	Forpus Passerinus	247317	-48390.350	-47243.171
Green-Rumped Parrotlet	Forpus Passerinus	247316	-46475.868	-46176.788
Green-Rumped Parrotlet	Forpus Passerinus	247311	-58736.118	-57137.346
Green-Rumped Parrotlet	Forpus Passerinus	247318	-46285.503	-45915.103
Green-Rumped Parrotlet	Forpus Passerinus	247320	-45467.502	-45005.369
Green-Rumped Parrotlet	Forpus Passerinus	247306	-81438.205	-79924.616
Green-Rumped Parrotlet	Forpus Passerinus	247308	-70020.178	-69744.988
Green-Rumped Parrotlet	Forpus Passerinus	247309	-65702.797	-65205.926
Green-Rumped Parrotlet	Forpus Passerinus	247307	-78728.950	-77772.469
Green-Rumped Parrotlet	Forpus Passerinus	247314	-51119.746	-51005.240
Green-Rumped Parrotlet	Forpus Passerinus	247313	-53113.215	-52903.807
Green-Rumped Parrotlet	Forpus Passerinus	247312	-52994.566	-52790.019
Green-Rumped Parrotlet	Forpus Passerinus	247315	-49246.921	-48723.211
HumpbackWhale	Megaptera Novaeangliae	117297	-548022.693	-526873.772
HumpbackWhale	Megaptera Novaeangliae	247348	-521097.280	-502595.152
HumpbackWhale	Megaptera Novaeangliae	247346	-773377.381	-767506.236
HumpbackWhale	Megaptera Novaeangliae	247347	-767723.914	-761601.397
HumpbackWhale	Megaptera Novaeangliae	247349	-478706.382	-471161.905
HumpbackWhale	Megaptera Novaeangliae	110847	-826615.555	-798885.310
HumpbackWhale	Megaptera Novaeangliae	247350	-419882.805	-419775.681
HumpbackWhale	Megaptera Novaeangliae	247351	-391880.834	-387198.604
HumpbackWhale	Megaptera Novaeangliae	247345	-810386.929	-796253.018
HumpbackWhale	Megaptera Novaeangliae	118144	-792182.892	-780444.216
KillerWhale	Orcinus Orca	123105	-432973.923	-424584.019
KillerWhale	Orcinus Orca	123132	-140193.131	-138727.901
KillerWhale	Orcinus Orca	128204	-168196.074	-160463.479
KillerWhale	Orcinus Orca	128216	-198445.784	-195067.613
KillerWhale	Orcinus Orca	128218	-121446.563	-118352.527
KillerWhale	Orcinus Orca	128217	-190949.441	-183133.371
KillerWhale	Orcinus Orca	120761	-573589.679	-570422.171
KillerWhale	Orcinus Orca	123145	-163817.351	-161442.297
KillerWhale	Orcinus Orca	123142	-154040.432	-151901.131
KillerWhale	Orcinus Orca	123136	-131235.683	-131644.198
KillerWhale	Orcinus Orca	128215	-156909.919	-152249.765
KillerWhale	Orcinus Orca	128223	-305318.617	-296809.709
KillerWhale	Orcinus Orca	128222	-289916.131	-286694.794
KillerWhale	Orcinus Orca	128214	-270077.696	-258823.810
KillerWhale	Orcinus Orca	123125	-123156.980	-121483.923
KillerWhale	Orcinus Orca	120595	-151661.873	-150435.281
KillerWhale	Orcinus Orca	123124	-120279.055	-118961.712
Northern Cardinal	Cardinalis Cardinalis	94283	-52805.342	-52002.781
Northern Cardinal	Cardinalis Cardinalis	84683	-57270.641	-56955.254

Table S3: Evidence for each model and recording

**Common Name**	**Species**	**Recording ID**	**$logZ_{ARMA}$**	**$logZ_{Fractal}$**
Northern Cardinal	Cardinalis Cardinalis	98872	-42066.411	-41423.089
Northern Cardinal	Cardinalis Cardinalis	130905	-109891.235	-109272.964
Northern Cardinal	Cardinalis Cardinalis	100794	-39898.600	-39031.502
Northern Cardinal	Cardinalis Cardinalis	107306	-62063.303	-60720.013
Northern Cardinal	Cardinalis Cardinalis	100744	-22895.722	-22269.722
Northern Cardinal	Cardinalis Cardinalis	176244	-21885.985	-21544.407
Northern Cardinal	Cardinalis Cardinalis	57975	-39247.943	-38437.952
Northern Cardinal	Cardinalis Cardinalis	84706	-21127.513	-21146.974
Northern Cardinal	Cardinalis Cardinalis	49063	-26133.129	-25743.409
Northern Cardinal	Cardinalis Cardinalis	100765	-16442.763	-16176.401
Northern Cardinal	Cardinalis Cardinalis	134151	-55531.109	-56620.914
Northern Cardinal	Cardinalis Cardinalis	100764	-34899.342	-34361.578
Northern Cardinal	Cardinalis Cardinalis	94288	-51800.517	-51315.248
Northern Cardinal	Cardinalis Cardinalis	191165	-57790.984	-58136.652
Northern Cardinal	Cardinalis Cardinalis	100747	-19422.578	-18575.069
Northern Cardinal	Cardinalis Cardinalis	107279	-51054.612	-50126.685
Northern Cardinal	Cardinalis Cardinalis	107278	-38613.111	-38061.448
Northern Cardinal	Cardinalis Cardinalis	176241	-134898.854	-132564.579
NorthernMockingbird	Mimus Polyglottos	23403	-16636.578	-16119.087
NorthernMockingbird	Mimus Polyglottos	197002	-79591.503	-79379.679
NorthernMockingbird	Mimus Polyglottos	94375	-50434.260	-49589.780
NorthernMockingbird	Mimus Polyglottos	94372	-17549.327	-17068.028
NorthernMockingbird	Mimus Polyglottos	23402	-214811.792	-213829.236
NorthernMockingbird	Mimus Polyglottos	94373	-54122.600	-53402.524
NorthernMockingbird	Mimus Polyglottos	94374	-81665.854	-80918.618
NorthernMockingbird	Mimus Polyglottos	166628	-98219.411	-97679.120
NorthernMockingbird	Mimus Polyglottos	85196	-53277.152	-52975.466
NorthernMockingbird	Mimus Polyglottos	85198	-46588.189	-46189.431
NorthernMockingbird	Mimus Polyglottos	118628	-63639.453	-63260.049
NorthernMockingbird	Mimus Polyglottos	85197	-101215.363	-100572.679
NorthernMockingbird	Mimus Polyglottos	22932	-129756.114	-127436.135
NorthernMockingbird	Mimus Polyglottos	56847	-96446.737	-95980.903
NorthernMockingbird	Mimus Polyglottos	22933	-39394.233	-38795.946
NorthernMockingbird	Mimus Polyglottos	50223	-43640.090	-43097.882
NorthernMockingbird	Mimus Polyglottos	85192	-106318.123	-104465.324
NorthernMockingbird	Mimus Polyglottos	85193	-105845.245	-104351.636
NorthernMockingbird	Mimus Polyglottos	118613	-43986.502	-43495.291
NorthernMockingbird	Mimus Polyglottos	100752	-46701.769	-45854.954
Rose-breasted Grosbeak	Pheucticus Ludovicanius	84866	-22018.203	-21986.915
Rose-breasted Grosbeak	Pheucticus Ludovicanius	59210	-93814.718	-92032.741
Rose-breasted Grosbeak	Pheucticus Ludovicanius	94313	-25984.359	-25544.751
Rose-breasted Grosbeak	Pheucticus Ludovicanius	192210	-26971.147	-26886.718
Rose-breasted Grosbeak	Pheucticus Ludovicanius	191158	-246557.973	-245739.764
Rose-breasted Grosbeak	Pheucticus Ludovicanius	84864	-30955.573	-29955.025
Rose-breasted Grosbeak	Pheucticus Ludovicanius	113502	-107209.629	-106667.270

Table S3: Evidence for each model and recording

**Common Name**	**Species**	**Recording ID**	**$\log Z_{ARMA}$**	**$\log Z_{Fractal}$**
Rose-breasted Grosbeak	Pheucticus Ludovicanius	84865	-76971.034	-76197.240
Rose-breasted Grosbeak	Pheucticus Ludovicanius	176167	-20689.632	-20570.714
Rose-breasted Grosbeak	Pheucticus Ludovicanius	16980	-19699.527	-19451.291
Rose-breasted Grosbeak	Pheucticus Ludovicanius	59213	-77351.060	-75011.846
Rose-breasted Grosbeak	Pheucticus Ludovicanius	74906	-22211.425	-21836.070
Rose-breasted Grosbeak	Pheucticus Ludovicanius	49615	-119708.028	-117840.344
Rose-breasted Grosbeak	Pheucticus Ludovicanius	94327	-27710.011	-27299.852
Rose-breasted Grosbeak	Pheucticus Ludovicanius	53163	-30847.217	-30360.474
Rose-breasted Grosbeak	Pheucticus Ludovicanius	59206	-39597.930	-38127.160
Rose-breasted Grosbeak	Pheucticus Ludovicanius	191267	-24249.868	-23842.779
Rose-breasted Grosbeak	Pheucticus Ludovicanius	125225	-50961.995	-50788.342
Rose-breasted Grosbeak	Pheucticus Ludovicanius	50268	-75051.185	-74638.181
Rose-breasted Grosbeak	Pheucticus Ludovicanius	107970	-23261.034	-23156.049
Ryukyu Scops Owl	Otus elegans	247327	-80476.546	-79859.060
Ryukyu Scops Owl	Otus elegans	247329	-76792.454	-75507.917
Ryukyu Scops Owl	Otus elegans	247328	-82324.011	-80363.198
Ryukyu Scops Owl	Otus elegans	247326	-85326.910	-83125.243
Ryukyu Scops Owl	Otus elegans	247332	-68866.032	-68131.585
Ryukyu Scops Owl	Otus elegans	247335	-60010.966	-59814.633
Ryukyu Scops Owl	Otus elegans	247334	-65970.054	-64122.803
Ryukyu Scops Owl	Otus elegans	247333	-69219.889	-66073.076
Ryukyu Scops Owl	Otus elegans	247341	-50512.535	-49223.963
Ryukyu Scops Owl	Otus elegans	247324	-157307.250	-152451.928
Ryukyu Scops Owl	Otus elegans	247323	-153062.695	-152265.168
Ryukyu Scops Owl	Otus elegans	247340	-50385.487	-49656.594
Ryukyu Scops Owl	Otus elegans	247322	-473737.162	-468518.330
Ryukyu Scops Owl	Otus elegans	247325	-102897.504	-102478.117
Ryukyu Scops Owl	Otus elegans	247338	-60737.689	-59160.190
Ryukyu Scops Owl	Otus elegans	247336	-59340.431	-59170.105
Ryukyu Scops Owl	Otus elegans	247331	-71325.082	-69471.678
Ryukyu Scops Owl	Otus elegans	247330	-75057.974	-73038.113
Ryukyu Scops Owl	Otus elegans	247337	-60786.682	-58576.354
Ryukyu Scops Owl	Otus elegans	247339	-55964.133	-54910.421
Swainson’s Thrush	Catharus Ustulatus	4240	-43393.792	-42226.174
Swainson’s Thrush	Catharus Ustulatus	93793	-111380.152	-110066.860
Swainson’s Thrush	Catharus Ustulatus	136190	-71842.990	-70127.565
Swainson’s Thrush	Catharus Ustulatus	93794	-58971.602	-58295.814
Swainson’s Thrush	Catharus Ustulatus	130939	-40166.093	-39956.474
Swainson’s Thrush	Catharus Ustulatus	119444	-169239.194	-165821.361
Swainson’s Thrush	Catharus Ustulatus	121953	-97896.700	-95615.643
Swainson’s Thrush	Catharus Ustulatus	118694	-25778.960	-24946.607
Swainson’s Thrush	Catharus Ustulatus	136156	-64219.599	-63351.881
Swainson’s Thrush	Catharus Ustulatus	4221	-55063.301	-54975.482
Swainson’s Thrush	Catharus Ustulatus	133353	-52499.443	-51666.589
Swainson’s Thrush	Catharus Ustulatus	126471	-10955.040	-10944.024

Table S3: Evidence for each model and recording

Common Name	Species	Recording ID	$logZ_{ARMA}$	$logZ_{Fractal}$
Swainson's Thrush	Catharus Ustulatus	171721	-84817.650	-84808.460
Swainson's Thrush	Catharus Ustulatus	131468	-196203.374	-195501.296
Swainson's Thrush	Catharus Ustulatus	179528	-148784.740	-147716.265
Swainson's Thrush	Catharus Ustulatus	188871	-141951.004	-142036.274
Swainson's Thrush	Catharus Ustulatus	119446	-51393.278	-50111.749
Swainson's Thrush	Catharus Ustulatus	119447	-98140.128	-95864.018
Swainson's Thrush	Catharus Ustulatus	130994	-63592.373	-63412.329
Swainson's Thrush	Catharus Ustulatus	4232	-47485.660	-46194.469
Swainson's Thrush	Catharus Ustulatus	100877	-27849.039	-27404.636
Veery	Catharus Fuscescens	27193	-89242.775	-86912.627
Veery	Catharus Fuscescens	3663	-111928.762	-108941.159
Veery	Catharus Fuscescens	3648	-70730.450	-68333.297
Veery	Catharus Fuscescens	3646	-185506.171	-178391.035
Veery	Catharus Fuscescens	188824	-102880.217	-101196.628
Veery	Catharus Fuscescens	146568	-72100.039	-70667.121
Veery	Catharus Fuscescens	26770	-78369.882	-76206.664
Veery	Catharus Fuscescens	67777	-84750.960	-82578.194
Veery	Catharus Fuscescens	135727	-67752.518	-67998.813
Veery	Catharus Fuscescens	135720	-81654.589	-79794.152
Veery	Catharus Fuscescens	67782	-101963.320	-98917.979
Veery	Catharus Fuscescens	136577	-84044.587	-82102.284
Veery	Catharus Fuscescens	188826	-97792.577	-96699.310
Veery	Catharus Fuscescens	135714	-90385.421	-88433.348
Veery	Catharus Fuscescens	164574	-142922.840	-141876.606
Veery	Catharus Fuscescens	121915	-136196.934	-134074.928
White-throated Sparrow	Zonotrichia Albicollis	188878	-120356.252	-119063.370
White-throated Sparrow	Zonotrichia Albicollis	133396	-57466.306	-56589.850
White-throated Sparrow	Zonotrichia Albicollis	15594	-69988.521	-67936.828
White-throated Sparrow	Zonotrichia Albicollis	66745	-91623.401	-88367.446
White-throated Sparrow	Zonotrichia Albicollis	66742	-123277.224	-119308.313
White-throated Sparrow	Zonotrichia Albicollis	169036	-100813.535	-100701.246
White-throated Sparrow	Zonotrichia Albicollis	66744	-40570.543	-39466.643
White-throated Sparrow	Zonotrichia Albicollis	84685	-71621.891	-70452.798
White-throated Sparrow	Zonotrichia Albicollis	66766	-75865.416	-73046.967
White-throated Sparrow	Zonotrichia Albicollis	15603	-59551.788	-56758.640
White-throated Sparrow	Zonotrichia Albicollis	107292	-78928.551	-77677.144
White-throated Sparrow	Zonotrichia Albicollis	137668	-52934.102	-52828.032
White-throated Sparrow	Zonotrichia Albicollis	15605	-51429.876	-50536.613
White-throated Sparrow	Zonotrichia Albicollis	15586	-78378.964	-77748.294
White-throated Sparrow	Zonotrichia Albicollis	15562	-50594.559	-49737.115
White-throated Sparrow	Zonotrichia Albicollis	73979	-61413.919	-61245.633
White-throated Sparrow	Zonotrichia Albicollis	136579	-91786.013	-90053.745
White-throated Sparrow	Zonotrichia Albicollis	15601	-59448.299	-58066.286
White-throated Sparrow	Zonotrichia Albicollis	169021	-155705.563	-153802.930
Wolves	Canis Lupus	56761	-98473.149	-93170.114

Table S3: Evidence for each model and recording

Common Name	Species	Recording ID	$logZ_{ARMA}$	$logZ_{Fractal}$
Wolves	Canis Lupus	116310	-195711.331	-192509.998
Wolves	Canis Lupus	116373	-52788.794	-50083.748
Wolves	Canis Lupus	116387	-106894.387	-105587.378
Wolves	Canis Lupus	116309	-156450.357	-154219.616
Wolves	Canis Lupus	116385	-38174.005	-37324.899
Wolves	Canis Lupus	116382	-413558.530	-406901.127
Wolves	Canis Lupus	116383	-406803.545	-402452.630
Wood Thrush	HylocichlaMustelina	11342	-9495.196	-9303.715
Wood Thrush	HylocichlaMustelina	11316	-117225.966	-115652.200
Wood Thrush	HylocichlaMustelina	176115	-15638.794	-15402.585
Wood Thrush	HylocichlaMustelina	11317	-27305.646	-27001.984
Wood Thrush	HylocichlaMustelina	176200	-114813.107	-112133.652
Wood Thrush	HylocichlaMustelina	40807	-127026.389	-123304.906
Wood Thrush	HylocichlaMustelina	107333	-16467.682	-16144.301
Wood Thrush	HylocichlaMustelina	94312	-37452.498	-36671.545
Wood Thrush	HylocichlaMustelina	94325	-58132.384	-57259.103
Wood Thrush	HylocichlaMustelina	84903	-36446.665	-36108.800
Wood Thrush	HylocichlaMustelina	11346	-164461.301	-162049.756
Wood Thrush	HylocichlaMustelina	191174	-171875.067	-171811.936
Wood Thrush	HylocichlaMustelina	176144	-86456.107	-85112.034
Wood Thrush	HylocichlaMustelina	107324	-50333.568	-49197.998
Wood Thrush	HylocichlaMustelina	94417	-31122.136	-30211.705
Wood Thrush	HylocichlaMustelina	176259	-29311.295	-28727.300
Wood Thrush	HylocichlaMustelina	94416	-55000.725	-53855.185
Wood Thrush	HylocichlaMustelina	168331	-37710.987	-37503.962
Wood Thrush	HylocichlaMustelina	100890	-39348.458	-38433.984
Wood Thrush	HylocichlaMustelina	11308	-142602.321	-140984.386

Table S4: Parameters for the ARMA model for each recording

Common Name	Species	Recording ID	*A*	σ_A	τ	σ_τ	*f*	σ_f	φ	σ_φ	κ	σ_κ
Adelie Penguin	Pygoscelis Adeliae	247294	5.840	0.002	0.071	0.001	0.033	0.003	0.139	0.003	4.730	0.005
Adelie Penguin	Pygoscelis Adeliae	247293	5.871	0.002	0.073	0.001	0.061	0.003	0.163	0.003	4.826	0.005
Adelie Penguin	Pygoscelis Adeliae	247295	6.080	0.003	0.074	0.000	0.138	0.005	0.359	0.004	5.238	0.005
Adelie Penguin	Pygoscelis Adeliae	247301	8.921	0.002	0.066	0.000	0.222	0.001	0.301	0.001	8.290	0.001
Adelie Penguin	Pygoscelis Adeliae	247300	5.847	0.001	0.080	0.000	0.216	0.003	0.339	0.002	5.253	0.005
Adelie Penguin	Pygoscelis Adeliae	247297	5.888	0.003	0.067	0.001	0.113	0.002	0.221	0.000	5.022	0.002
Adelie Penguin	Pygoscelis Adeliae	247299	5.804	0.001	0.072	0.000	0.104	0.001	0.171	0.000	4.831	0.002
Adelie Penguin	Pygoscelis Adeliae	247298	9.031	0.000	0.072	0.000	0.111	0.000	0.185	0.000	8.101	0.000
Adelie Penguin	Pygoscelis Adeliae	247296	9.656	0.002	0.072	0.000	0.091	0.002	0.180	0.001	8.669	0.003
Adelie Penguin	Pygoscelis Adeliae	247302	5.823	0.002	0.062	0.000	0.237	0.000	0.221	0.001	5.186	0.002
Adelie Penguin	Pygoscelis Adeliae	247303	5.780	0.005	0.072	0.001	0.171	0.007	0.424	0.007	5.025	0.008
Adelie Penguin	Pygoscelis Adeliae	247304	5.826	0.002	0.062	0.000	0.117	0.005	0.445	0.003	4.968	0.007
Altamira Oriole	Icterus Gularis	12515	5.473	0.001	0.068	0.000	0.280	0.001	0.359	0.002	5.220	0.001
Altamira Oriole	Icterus Gularis	105591	5.682	0.006	0.060	0.000	0.123	0.015	0.234	0.001	4.756	0.011
Altamira Oriole	Icterus Gularis	20382	5.680	0.006	0.067	0.001	0.215	0.008	0.397	0.007	4.966	0.012
Altamira Oriole	Icterus Gularis	140215	9.056	0.009	56.546	0.002	0.052	0.008	0.201	0.006	7.989	0.010
Altamira Oriole	Icterus Gularis	20792	5.605	0.002	0.069	0.000	0.042	0.002	0.213	0.003	4.716	0.005
Altamira Oriole	Icterus Gularis	12519	5.555	0.004	0.067	0.000	0.095	0.002	0.162	0.002	4.548	0.003
Altamira Oriole	Icterus Gularis	131218	9.026	0.009	72.243	0.001	0.004	0.006	0.251	0.002	8.148	0.011
Bach - Brandenburg Concerto 1 long			7.579	0.000	0.061	0.000	0.125	0.000	0.632	0.000	7.222	0.000
Bach - Brandenburg Concerto 1 long2			8.544	0.000	0.062	0.000	0.328	0.000	0.351	0.000	8.211	0.000
Baltimore Oriole	Icterus Galbula	105614	5.654	0.003	0.064	0.000	0.197	0.003	0.335	0.000	5.016	0.008
Baltimore Oriole	Icterus Galbula	105640	5.686	0.004	0.059	0.000	0.138	0.005	0.241	0.001	4.952	0.004
Baltimore Oriole	Icterus Galbula	12481	5.963	0.002	0.071	0.001	0.140	0.006	0.189	0.001	5.102	0.005
Baltimore Oriole	Icterus Galbula	164538	8.925	0.000	0.057	0.002	0.004	0.004	0.112	0.003	7.890	0.004
Baltimore Oriole	Icterus Galbula	163341	9.435	0.013	0.050	0.004	0.143	0.009	0.146	0.007	8.405	0.014
Baltimore Oriole	Icterus Galbula	94326	5.868	0.002	0.061	0.000	0.082	0.002	0.166	0.001	5.091	0.003
Baltimore Oriole	Icterus Galbula	195752	9.520	0.002	89.176	0.002	0.015	0.002	0.117	0.002	8.397	0.010
Baltimore Oriole	Icterus Galbula	105630	5.780	0.001	0.065	0.000	0.028	0.004	0.388	0.002	5.070	0.002
Baltimore Oriole	Icterus Galbula	105642	5.714	0.010	60.575	0.001	0.069	0.013	0.432	0.011	4.805	0.021
Baltimore Oriole	Icterus Galbula	112699	5.598	0.007	64.493	0.001	0.176	0.011	0.448	0.010	4.737	0.014
Baltimore Oriole	Icterus Galbula	112697	5.747	0.001	96.288	0.000	0.023	0.001	0.268	0.001	4.899	0.001
Baltimore Oriole	Icterus Galbula	105627	5.802	0.003	0.062	0.000	0.033	0.003	0.302	0.002	4.843	0.009
Baltimore Oriole	Icterus Galbula	112698	5.527	0.012	0.073	0.001	0.198	0.016	0.503	0.013	4.804	0.023
Baltimore Oriole	Icterus Galbula	12482	4.907	0.044	85.500	16.637	0.021	0.013	0.352	0.042	3.883	0.056
Baltimore Oriole	Icterus Galbula	12483	5.927	0.001	0.062	0.000	0.121	0.003	0.360	0.001	5.184	0.002
Baltimore Oriole	Icterus Galbula	110227	5.807	0.003	0.066	0.000	0.038	0.003	0.191	0.001	5.194	0.001
Baltimore Oriole	Icterus Galbula	192118	9.417	0.002	0.150	0.000	0.019	0.001	0.145	0.000	8.559	0.001

Table S4: Parameters for the ARMA model for each recording

Common Name	Species	Recording ID	*A*	σ_A	τ	σ_τ	*f*	σ_f	φ	σ_φ	κ	σ_κ
Baltimore Oriole	Icterus Galbula	113500	5.864	0.001	0.068	0.000	0.036	0.002	0.277	0.001	5.055	0.001
Baltimore Oriole	Icterus Galbula	113501	5.608	0.010	98.076	0.001	0.056	0.012	0.364	0.011	4.564	0.022
Baltimore Oriole	Icterus Galbula	192119	9.190	0.000	0.068	0.000	0.005	0.000	0.150	0.000	8.208	0.004
Barred Owl	Strix Varia	134150	9.601	0.000	0.068	0.000	0.083	0.000	0.241	0.000	8.727	0.000
Barred Owl	Strix Varia	135412	9.362	0.001	0.062	0.000	0.028	0.000	0.737	0.002	10.063	0.001
Barred Owl	Strix Varia	187078	9.722	0.000	0.064	0.000	0.075	0.000	0.493	0.000	8.930	0.000
Barred Owl	Strix Varia	128933	9.589	0.001	0.056	0.000	0.039	0.000	0.119	0.000	8.759	0.002
Barred Owl	Strix Varia	79462	5.845	0.002	0.072	0.001	0.009	0.002	0.177	0.004	4.798	0.007
Barred Owl	Strix Varia	125364	6.049	0.002	0.067	0.000	0.042	0.003	0.263	0.003	5.042	0.006
Barred Owl	Strix Varia	49708	5.725	0.007	0.065	0.000	0.278	0.010	0.516	0.006	5.204	0.005
Barred Owl	Strix Varia	4549	5.748	0.001	0.075	0.000	0.251	0.000	0.453	0.003	5.439	0.001
Barred Owl	Strix Varia	128926	9.579	0.000	0.061	0.000	0.117	0.001	0.293	0.000	8.864	0.000
Barred Owl	Strix Varia	4548	5.692	0.002	0.071	0.000	0.109	0.002	0.209	0.002	4.742	0.002
Barred Owl	Strix Varia	128931	8.669	0.001	0.063	0.000	0.066	0.000	0.167	0.000	7.656	0.002
Barred Owl	Strix Varia	191186	8.999	0.001	0.071	0.001	0.004	0.000	0.123	0.000	8.424	0.002
Barred Owl	Strix Varia	128930	9.691	0.001	0.063	0.000	0.064	0.001	0.148	0.000	8.920	0.001
Barred Owl	Strix Varia	36950	5.658	0.007	0.070	0.001	0.215	0.004	0.392	0.003	4.971	0.018
Barred Owl	Strix Varia	110209	6.054	0.000	0.068	0.000	0.041	0.001	0.299	0.001	5.091	0.003
Barred Owl	Strix Varia	195216	5.907	0.005	0.068	0.001	0.024	0.005	0.125	0.003	4.819	0.002
Barred Owl	Strix Varia	175898	6.063	0.001	0.067	0.000	0.165	0.001	0.399	0.002	5.394	0.002
Barred Owl	Strix Varia	163907	5.857	0.008	0.070	0.000	0.167	0.003	0.440	0.002	5.209	0.003
Barred Owl	Strix Varia	201156	9.620	0.002	0.063	0.002	0.041	0.012	0.071	0.001	8.581	0.008
Barred Owl	Strix Varia	105433	6.300	0.001	0.058	0.001	0.022	0.001	0.030	0.000	5.169	0.003
Barred Owl	Strix Varia	72743	5.511	0.006	0.071	0.001	0.202	0.008	0.316	0.007	4.743	0.012
Barred Owl	Strix Varia	128924	9.707	0.005	0.064	0.001	0.133	0.007	0.278	0.007	8.858	0.009
Barred Owl	Strix Varia	134149	9.317	0.013	0.060	0.001	0.026	0.003	0.163	0.002	8.540	0.011
Barred Owl	Strix Varia	52320	5.912	0.001	0.069	0.000	0.259	0.002	0.279	0.001	5.335	0.000
Barred Owl	Strix Varia	128925	9.433	0.003	0.157	0.001	0.139	0.004	0.145	0.003	8.550	0.006
Brown Thrasher	Toxostoma Rufum	67309	5.953	0.001	0.056	0.001	0.054	0.007	0.284	0.004	4.985	0.011
Brown Thrasher	Toxostoma Rufum	148978	5.718	0.002	0.060	0.002	0.006	0.003	0.053	0.001	4.586	0.004
Brown Thrasher	Toxostoma Rufum	100774	5.802	0.001	0.068	0.000	0.032	0.000	0.194	0.000	4.924	0.000
Brown Thrasher	Toxostoma Rufum	67312	5.876	0.002	0.064	0.000	0.057	0.001	0.078	0.000	4.910	0.003
Brown Thrasher	Toxostoma Rufum	190985	5.900	0.002	0.064	0.000	0.042	0.001	0.278	0.005	5.005	0.001
Brown Thrasher	Toxostoma Rufum	164590	5.919	0.001	0.069	0.000	0.044	0.003	0.113	0.001	4.863	0.002
Brown Thrasher	Toxostoma Rufum	176256	6.037	0.000	0.074	0.000	0.022	0.000	0.159	0.000	5.290	0.001
Brown Thrasher	Toxostoma Rufum	11174	5.918	0.000	0.066	0.000	0.012	0.000	0.106	0.000	4.993	0.000
Brown Thrasher	Toxostoma Rufum	105379	6.006	0.002	52.918	0.007	0.036	0.001	0.018	0.001	4.977	0.006
Brown Thrasher	Toxostoma Rufum	11180	5.854	0.004	0.069	0.001	0.094	0.005	0.222	0.006	4.876	0.006

Table S4: Parameters for the ARMA model for each recording

Common Name	Species	Recording ID	A	σ_A	τ	σ_τ	f	σ_f	φ	σ_φ	κ	σ_κ
Brown Thrasher	Toxostoma Rufum	84769	5.797	0.004	0.065	0.001	0.147	0.006	0.303	0.004	4.938	0.008
Brown Thrasher	Toxostoma Rufum	11175	6.021	0.001	0.065	0.001	0.066	0.001	0.123	0.001	5.011	0.009
Brown Thrasher	Toxostoma Rufum	84766	6.074	0.000	0.059	0.000	0.045	0.001	0.217	0.001	5.113	0.001
Brown Thrasher	Toxostoma Rufum	84768	5.759	0.002	0.065	0.000	0.079	0.003	0.214	0.002	4.730	0.005
Brown Thrasher	Toxostoma Rufum	94279	6.155	0.002	0.068	0.000	0.080	0.001	0.137	0.000	5.186	0.005
Brown Thrasher	Toxostoma Rufum	100779	5.982	0.001	0.067	0.000	0.017	0.003	0.298	0.000	4.968	0.002
Brown Thrasher	Toxostoma Rufum	60091	5.798	0.003	0.059	0.001	0.134	0.002	0.182	0.001	5.117	0.001
Brown Thrasher	Toxostoma Rufum	181512	9.607	0.001	0.063	0.000	0.010	0.000	0.085	0.000	8.559	0.000
Brown Thrasher	Toxostoma Rufum	100754	5.952	0.000	0.074	0.000	0.038	0.000	0.117	0.000	5.042	0.001
Brown Thrasher	Toxostoma Rufum	176262	6.340	0.001	0.066	0.000	0.019	0.000	0.139	0.000	5.456	0.001
CanyonWren	CatherpesMexicanus	197170	9.061	0.003	0.269	0.001	0.056	0.004	0.333	0.011	8.100	0.011
CanyonWren	CatherpesMexicanus	120253	5.880	0.003	0.054	0.001	0.063	0.005	0.198	0.001	5.033	0.001
CanyonWren	CatherpesMexicanus	197171	9.276	0.002	0.193	0.001	0.079	0.001	0.188	0.001	8.375	0.001
CanyonWren	CatherpesMexicanus	197943	9.634	0.000	0.169	0.000	0.042	0.002	0.083	0.000	8.723	0.001
CanyonWren	CatherpesMexicanus	44687	9.589	0.001	0.297	0.000	0.206	0.001	0.372	0.000	9.260	0.000
CanyonWren	CatherpesMexicanus	105214	5.789	0.001	0.181	0.000	0.031	0.001	0.142	0.001	4.870	0.003
CanyonWren	CatherpesMexicanus	9043	5.967	0.005	0.165	0.001	0.052	0.009	0.261	0.008	5.054	0.008
CanyonWren	CatherpesMexicanus	9044	5.721	0.007	90.209	0.002	0.040	0.010	0.283	0.010	4.760	0.017
CanyonWren	CatherpesMexicanus	37733	5.314	0.007	75.789	0.066	0.051	0.008	0.145	0.008	4.215	0.014
CanyonWren	CatherpesMexicanus	105240	5.731	0.009	75.239	0.072	0.135	0.012	0.284	0.011	4.886	0.019
CanyonWren	CatherpesMexicanus	110950	5.686	0.001	0.066	0.000	0.171	0.003	0.278	0.002	5.027	0.013
CanyonWren	CatherpesMexicanus	148337	9.249	0.001	0.069	0.000	0.148	0.003	0.399	0.001	8.625	0.002
CanyonWren	CatherpesMexicanus	189253	9.252	0.007	78.002	0.183	0.117	0.011	0.281	0.010	8.298	0.017
CanyonWren	CatherpesMexicanus	148381	9.436	0.002	0.060	0.000	0.194	0.004	0.473	0.002	8.850	0.004
CanyonWren	CatherpesMexicanus	120251	6.149	0.001	0.058	0.000	0.048	0.002	0.146	0.001	5.162	0.004
CanyonWren	CatherpesMexicanus	45080	5.540	0.009	0.069	0.001	0.263	0.011	0.444	0.009	4.934	0.016
CanyonWren	CatherpesMexicanus	120205	5.945	0.000	0.060	0.000	0.233	0.001	0.232	0.000	5.355	0.001
CanyonWren	CatherpesMexicanus	44685	9.157	0.003	0.272	0.001	0.079	0.001	0.285	0.001	8.627	0.002
CanyonWren	CatherpesMexicanus	163246	5.745	0.001	0.066	0.000	0.127	0.001	0.188	0.001	4.802	0.003
CanyonWren	CatherpesMexicanus	63220	5.704	0.001	0.068	0.000	0.157	0.002	0.347	0.006	5.030	0.002
CanyonWren	CatherpesMexicanus	63218	5.814	0.003	0.071	0.001	0.150	0.005	0.223	0.004	4.953	0.007
CanyonWren	CatherpesMexicanus	9041	5.859	0.006	0.070	0.001	0.224	0.008	0.315	0.007	5.169	0.011
CanyonWren	CatherpesMexicanus	63219	5.706	0.006	0.066	0.001	0.139	0.008	0.255	0.007	4.810	0.012
Common Loon	Gavia Immer	197042	9.685	0.002	0.068	0.000	0.012	0.000	0.465	0.000	9.141	0.000
Common Loon	Gavia Immer	72736	5.905	0.003	0.071	0.000	0.103	0.004	0.422	0.003	5.184	0.003
Common Loon	Gavia Immer	913	5.932	0.001	0.076	0.000	0.031	0.000	0.185	0.000	5.354	0.000
Common Loon	Gavia Immer	61479	5.709	0.000	0.072	0.000	0.264	0.000	0.511	0.001	5.542	0.000
Common Loon	Gavia Immer	61477	5.844	0.000	0.070	0.000	0.053	0.000	0.281	0.000	5.217	0.001

Table S4: Parameters for the ARMA model for each recording

Common Name	Species	Recording ID	A	σ_A	τ	σ_τ	f	σ_f	φ	σ_φ	κ	σ_κ
Common Loon	Gavia Immer	193551	6.403	0.001	0.068	0.001	0.005	0.002	0.092	0.003	5.251	0.006
Common Loon	Gavia Immer	72720	5.947	0.002	0.075	0.000	0.293	0.000	0.289	0.001	5.383	0.001
Common Loon	Gavia Immer	197036	9.521	0.000	0.077	0.000	0.129	0.001	0.501	0.000	9.044	0.000
Common Loon	Gavia Immer	927	6.060	0.001	0.068	0.000	0.099	0.000	0.304	0.000	5.471	0.001
Common Loon	Gavia Immer	918	5.742	0.001	0.072	0.000	0.018	0.001	0.319	0.001	5.033	0.000
Common Loon	Gavia Immer	916	5.807	0.002	0.071	0.000	0.023	0.001	0.462	0.004	5.167	0.001
EasternWood-Pewee	Contopus Virens	73930	5.885	0.000	0.067	0.000	0.089	0.000	0.202	0.000	5.305	0.001
EasternWood-Pewee	Contopus Virens	176147	5.929	0.001	0.070	0.000	0.021	0.001	0.345	0.000	5.344	0.001
EasternWood-Pewee	Contopus Virens	55546	5.865	0.001	0.070	0.000	0.058	0.001	0.280	0.001	5.091	0.001
EasternWood-Pewee	Contopus Virens	41163	5.602	0.004	0.073	0.001	0.049	0.001	0.221	0.001	4.609	0.002
EasternWood-Pewee	Contopus Virens	110260	5.727	0.003	0.069	0.000	0.089	0.006	0.397	0.004	4.938	0.012
EasternWood-Pewee	Contopus Virens	191222	9.564	0.003	0.058	0.000	0.006	0.000	0.398	0.003	8.783	0.003
EasternWood-Pewee	Contopus Virens	188886	9.441	0.001	0.063	0.000	0.006	0.000	0.089	0.000	8.574	0.006
EasternWood-Pewee	Contopus Virens	191199	9.506	0.001	0.071	0.000	0.004	0.000	0.429	0.001	8.787	0.001
EasternWood-Pewee	Contopus Virens	7392	5.819	0.002	0.064	0.000	0.046	0.002	0.600	0.000	5.291	0.001
EasternWood-Pewee	Contopus Virens	68577	5.678	0.000	0.068	0.000	0.086	0.001	0.213	0.000	4.769	0.002
EasternWood-Pewee	Contopus Virens	116518	8.769	0.000	0.062	0.000	0.117	0.000	0.250	0.001	7.883	0.000
EasternWood-Pewee	Contopus Virens	176204	5.905	0.001	0.064	0.000	0.065	0.000	0.275	0.000	5.308	0.001
EasternWood-Pewee	Contopus Virens	116519	9.100	0.000	0.067	0.000	0.055	0.003	0.217	0.000	8.439	0.001
EasternWood-Pewee	Contopus Virens	176281	5.853	0.000	0.069	0.000	0.016	0.000	0.309	0.000	5.149	0.000
EasternWood-Pewee	Contopus Virens	62943	6.268	0.000	0.068	0.000	0.023	0.000	0.368	0.001	5.297	0.000
EasternWood-Pewee	Contopus Virens	38530	5.784	0.000	0.071	0.000	0.093	0.000	0.249	0.000	4.888	0.000
EasternWood-Pewee	Contopus Virens	101800	5.950	0.000	0.063	0.000	0.186	0.000	0.347	0.001	5.232	0.000
EasternWood-Pewee	Contopus Virens	191166	8.858	0.001	0.070	0.000	0.100	0.002	0.573	0.000	8.322	0.001
Field Cricket	Gryllus	200359	5.760	0.003	0.074	0.001	0.009	0.002	0.171	0.004	4.692	0.014
Field Cricket	Gryllus	85154	5.681	0.002	0.274	0.000	0.001	0.000	0.262	0.001	4.943	0.004
Field Cricket	Gryllus	54805	5.489	0.006	0.068	0.002	0.130	0.009	0.194	0.006	4.549	0.017
Field Cricket	Gryllus	200846	5.760	0.001	0.075	0.000	0.031	0.000	0.096	0.000	4.816	0.000
Field Cricket	Gryllus	191240	9.631	0.001	0.068	0.000	0.313	0.001	0.243	0.001	9.247	0.001
Field Cricket	Gryllus	200801	5.711	0.004	0.071	0.001	0.015	0.006	0.112	0.001	4.667	0.004
Field Cricket	Gryllus	200710	5.655	0.003	55.718	0.076	0.088	0.002	0.036	0.006	4.815	0.007
Field Cricket	Gryllus	85329	5.711	0.002	0.069	0.000	0.106	0.001	0.308	0.002	4.882	0.001
Field Cricket	Gryllus	85185	5.839	0.003	0.068	0.001	0.039	0.005	0.155	0.003	4.797	0.005
Field Cricket	Gryllus	85328	5.676	0.000	0.066	0.000	0.124	0.002	0.263	0.001	5.016	0.002
Field Cricket	Gryllus	200796	5.652	0.004	98.122	0.001	0.048	0.010	0.232	0.003	4.570	0.019
Field Cricket	Gryllus	85187	5.853	0.006	0.334	0.003	0.061	0.008	0.074	0.003	4.788	0.014
Field Cricket	Gryllus	85189	6.012	0.003	0.063	0.002	0.093	0.002	0.133	0.002	4.984	0.011
Field Cricket	Gryllus	85186	5.921	0.004	99.982	0.002	0.006	0.005	0.130	0.004	4.849	0.011

Table S4: Parameters for the ARMA model for each recording

Common Name	Species	Recording ID	*A*	σ_A	τ	σ_τ	*f*	σ_f	φ	σ_φ	κ	σ_κ
Frog	Lithobates	71895	8.530	0.002	6.070	0.002	0.024	0.003	0.053	0.002	7.340	0.005
Frog	Lithobates	182118	5.724	0.000	0.063	0.000	0.165	0.000	0.228	0.000	5.325	0.000
Frog	Lithobates	163335	8.722	0.000	0.059	0.001	0.011	0.000	0.102	0.000	7.930	0.002
Frog	Lithobates	163334	8.716	0.002	0.063	0.001	0.021	0.001	0.062	0.000	7.729	0.002
Frog	Lithobates	182023	5.849	0.001	0.067	0.000	0.123	0.002	0.273	0.001	5.010	0.003
Frog	Lithobates	179100	5.720	0.001	0.066	0.001	0.043	0.001	0.117	0.001	4.623	0.002
Frog	Lithobates	138552	8.071	0.000	0.060	0.000	0.017	0.000	0.063	0.000	7.113	0.001
Frog	Lithobates	138394	9.402	0.000	0.055	0.000	0.021	0.000	0.121	0.000	8.487	0.000
Frog	Lithobates	138553	8.704	0.000	51.090	0.000	0.028	0.000	0.050	0.000	7.647	0.000
Frog	Lithobates	182026	5.835	0.001	0.068	0.000	0.022	0.002	0.241	0.001	4.797	0.003
Frog	Lithobates	136572	6.170	0.000	0.065	0.001	0.024	0.000	0.050	0.000	5.297	0.000
Gray Catbird	Dumetella Carolinensis	57999	5.865	0.001	0.067	0.000	0.074	0.000	0.236	0.002	4.833	0.006
Gray Catbird	Dumetella Carolinensis	107339	6.201	0.001	0.064	0.000	0.061	0.000	0.086	0.000	5.332	0.000
Gray Catbird	Dumetella Carolinensis	36955	6.157	0.000	0.056	0.000	0.051	0.000	0.093	0.000	5.150	0.001
Gray Catbird	Dumetella Carolinensis	11434	6.047	0.000	0.064	0.000	0.124	0.001	0.146	0.000	5.209	0.001
Gray Catbird	Dumetella Carolinensis	121989	6.050	0.002	0.062	0.000	0.049	0.001	0.147	0.001	4.983	0.003
Gray Catbird	Dumetella Carolinensis	22454	6.102	0.004	0.072	0.000	0.051	0.003	0.208	0.002	5.203	0.004
Gray Catbird	Dumetella Carolinensis	168306	9.099	0.000	0.061	0.000	0.009	0.001	0.036	0.000	8.079	0.000
Gray Catbird	Dumetella Carolinensis	121994	6.177	0.000	0.064	0.000	0.070	0.001	0.133	0.000	5.308	0.002
Gray Catbird	Dumetella Carolinensis	107340	6.271	0.001	0.063	0.000	0.003	0.000	0.112	0.000	5.166	0.004
Gray Catbird	Dumetella Carolinensis	176248	6.099	0.001	0.063	0.001	0.038	0.001	0.054	0.000	5.124	0.006
Gray Catbird	Dumetella Carolinensis	50269	6.025	0.002	0.065	0.000	0.073	0.002	0.238	0.001	5.012	0.002
Gray Catbird	Dumetella Carolinensis	191237	9.655	0.000	0.056	0.001	0.002	0.000	0.036	0.000	8.643	0.002
Gray Catbird	Dumetella Carolinensis	10211	6.197	0.005	0.063	0.000	0.051	0.005	0.254	0.003	5.149	0.009
Gray Catbird	Dumetella Carolinensis	10218	6.313	0.000	0.058	0.000	0.024	0.000	0.102	0.000	5.314	0.002
Gray Catbird	Dumetella Carolinensis	94321	5.916	0.002	0.059	0.001	0.043	0.004	0.114	0.001	4.825	0.006
Gray Catbird	Dumetella Carolinensis	93763	6.272	0.000	0.060	0.000	0.081	0.001	0.147	0.000	5.322	0.001
Green-Rumped Parrotlet	Forpus Passerinus	247305	6.205	0.003	0.066	0.001	0.072	0.004	0.226	0.004	5.236	0.004
Green-Rumped Parrotlet	Forpus Passerinus	247321	5.908	0.001	0.062	0.001	0.031	0.000	0.172	0.001	4.905	0.002
Green-Rumped Parrotlet	Forpus Passerinus	247319	5.534	0.000	0.075	0.000	0.042	0.002	0.101	0.000	4.546	0.001
Green-Rumped Parrotlet	Forpus Passerinus	247310	5.643	0.003	0.073	0.000	0.069	0.001	0.210	0.000	4.689	0.004
Green-Rumped Parrotlet	Forpus Passerinus	247317	5.598	0.005	0.074	0.001	0.128	0.007	0.195	0.007	4.692	0.010
Green-Rumped Parrotlet	Forpus Passerinus	247316	5.435	0.004	0.072	0.002	0.047	0.006	0.083	0.006	4.328	0.010
Green-Rumped Parrotlet	Forpus Passerinus	247311	5.605	0.001	0.066	0.000	0.186	0.001	0.236	0.002	4.885	0.002
Green-Rumped Parrotlet	Forpus Passerinus	247318	5.478	0.003	0.071	0.001	0.043	0.002	0.127	0.006	4.379	0.007
Green-Rumped Parrotlet	Forpus Passerinus	247320	5.710	0.002	0.066	0.001	0.066	0.000	0.200	0.001	4.708	0.003
Green-Rumped Parrotlet	Forpus Passerinus	247306	5.556	0.002	0.075	0.001	0.091	0.002	0.085	0.001	4.659	0.003
Green-Rumped Parrotlet	Forpus Passerinus	247308	5.481	0.003	0.245	0.007	0.022	0.005	0.040	0.005	4.377	0.008

Table S4: Parameters for the ARMA model for each recording

**Common Name**	**Species**	**Recording ID**	A	σ_A	τ	σ_τ	f	σ_f	φ	σ_φ	κ	σ_κ
Green-Rumped Parrotlet	Forpus Passerinus	247309	5.547	0.002	0.072	0.001	0.015	0.004	0.125	0.004	4.455	0.008
Green-Rumped Parrotlet	Forpus Passerinus	247307	5.828	0.008	0.059	0.000	0.021	0.016	0.211	0.017	4.891	0.014
Green-Rumped Parrotlet	Forpus Passerinus	247314	5.441	0.003	0.168	0.004	0.018	0.005	0.044	0.004	4.285	0.009
Green-Rumped Parrotlet	Forpus Passerinus	247313	5.483	0.002	0.073	0.001	0.022	0.003	0.095	0.002	4.362	0.002
Green-Rumped Parrotlet	Forpus Passerinus	247312	5.485	0.003	0.074	0.002	0.019	0.005	0.083	0.005	4.343	0.009
Green-Rumped Parrotlet	Forpus Passerinus	247315	5.620	0.002	0.063	0.000	0.105	0.003	0.244	0.001	4.655	0.004
HumpbackWhale	Megaptera Novaeangliae	117297	6.171	0.000	0.071	0.000	0.015	0.000	0.093	0.000	5.528	0.001
HumpbackWhale	Megaptera Novaeangliae	247348	6.121	0.000	0.071	0.000	0.030	0.000	0.088	0.000	5.496	0.000
HumpbackWhale	Megaptera Novaeangliae	247346	6.043	0.001	0.072	0.000	0.016	0.001	0.149	0.000	4.960	0.001
HumpbackWhale	Megaptera Novaeangliae	247347	6.028	0.000	0.069	0.000	0.027	0.000	0.100	0.000	4.921	0.001
HumpbackWhale	Megaptera Novaeangliae	247349	5.909	0.000	0.077	0.000	0.045	0.000	0.149	0.000	5.056	0.000
HumpbackWhale	Megaptera Novaeangliae	110847	6.303	0.000	0.069	0.000	0.127	0.000	0.503	0.000	6.053	0.000
HumpbackWhale	Megaptera Novaeangliae	247350	5.858	0.001	0.078	0.002	0.000	0.000	0.027	0.002	4.695	0.003
HumpbackWhale	Megaptera Novaeangliae	247351	5.951	0.001	0.071	0.000	0.048	0.001	0.220	0.001	4.940	0.001
HumpbackWhale	Megaptera Novaeangliae	247345	6.061	0.000	0.073	0.000	0.044	0.000	0.212	0.000	5.323	0.000
HumpbackWhale	Megaptera Novaeangliae	118144	6.097	0.001	0.072	0.000	0.038	0.001	0.287	0.001	5.199	0.001
KillerWhale	Orcinus Orca	123105	5.979	0.000	0.075	0.000	0.074	0.001	0.273	0.001	5.111	0.000
KillerWhale	Orcinus Orca	123132	5.948	0.001	0.066	0.000	0.053	0.001	0.243	0.001	5.000	0.001
KillerWhale	Orcinus Orca	128204	5.865	0.000	0.068	0.000	0.396	0.000	0.508	0.000	5.596	0.000
KillerWhale	Orcinus Orca	128216	6.052	0.000	0.064	0.000	0.017	0.000	0.285	0.000	5.234	0.000
KillerWhale	Orcinus Orca	128218	5.777	0.001	0.070	0.000	0.088	0.001	0.374	0.001	5.071	0.001
KillerWhale	Orcinus Orca	128217	6.063	0.000	0.070	0.000	0.024	0.000	0.319	0.000	5.460	0.001
KillerWhale	Orcinus Orca	120761	9.215	0.000	0.058	0.000	0.041	0.000	0.086	0.000	8.376	0.000
KillerWhale	Orcinus Orca	123145	6.009	0.000	0.069	0.000	0.080	0.001	0.163	0.000	5.164	0.000
KillerWhale	Orcinus Orca	123142	6.021	0.001	0.060	0.000	0.022	0.001	0.195	0.000	5.163	0.001
KillerWhale	Orcinus Orca	123136	6.355	0.001	0.054	0.000	0.032	0.001	0.193	0.002	5.177	0.002
KillerWhale	Orcinus Orca	128215	5.977	0.000	0.073	0.000	0.080	0.000	0.352	0.000	5.429	0.000
KillerWhale	Orcinus Orca	128223	8.988	0.000	0.072	0.000	0.093	0.000	0.266	0.000	8.676	0.000
KillerWhale	Orcinus Orca	128222	8.783	0.000	0.064	0.000	0.060	0.000	0.321	0.000	7.845	0.000
KillerWhale	Orcinus Orca	128214	5.997	0.002	0.068	0.000	0.195	0.000	0.548	0.000	5.873	0.000
KillerWhale	Orcinus Orca	123125	6.043	0.002	18.295	0.006	0.042	0.003	-0.020	0.003	5.018	0.005
KillerWhale	Orcinus Orca	120595	6.039	0.001	0.069	0.001	0.015	0.001	0.096	0.001	4.982	0.007
KillerWhale	Orcinus Orca	123124	5.896	0.001	0.074	0.000	0.050	0.001	0.229	0.001	4.866	0.000
Northern Cardinal	Cardinalis Cardinalis	94283	5.884	0.004	0.065	0.000	0.131	0.006	0.333	0.001	5.166	0.002
Northern Cardinal	Cardinalis Cardinalis	84683	5.826	0.001	0.063	0.000	0.043	0.002	0.287	0.002	4.769	0.005
Northern Cardinal	Cardinalis Cardinalis	98872	5.780	0.001	0.060	0.001	0.002	0.001	0.096	0.001	4.910	0.009
Northern Cardinal	Cardinalis Cardinalis	130905	6.077	0.001	0.061	0.000	0.067	0.001	0.267	0.002	5.288	0.001
Northern Cardinal	Cardinalis Cardinalis	100794	5.820	0.007	0.078	0.003	0.149	0.009	0.218	0.006	5.041	0.032

Table S4: Parameters for the ARMA model for each recording

Common Name	Species	Recording ID	*A*	σ_A	τ	σ_τ	*f*	σ_f	φ	σ_φ	κ	σ_κ
Northern Cardinal	Cardinalis Cardinalis	107306	5.818	0.002	0.062	0.000	0.165	0.002	0.453	0.004	5.467	0.002
Northern Cardinal	Cardinalis Cardinalis	100744	5.698	0.009	90.446	0.062	0.150	0.011	0.246	0.010	4.881	0.016
Northern Cardinal	Cardinalis Cardinalis	176244	5.661	0.007	86.397	0.001	0.140	0.014	0.516	0.014	5.003	0.011
Northern Cardinal	Cardinalis Cardinalis	57975	5.885	0.002	0.068	0.001	0.077	0.003	0.150	0.001	5.103	0.002
Northern Cardinal	Cardinalis Cardinalis	84706	5.770	0.002	84.393	0.001	0.018	0.002	0.174	0.003	4.711	0.004
Northern Cardinal	Cardinalis Cardinalis	49063	5.743	0.006	0.064	0.001	0.054	0.011	0.400	0.007	4.853	0.016
Northern Cardinal	Cardinalis Cardinalis	100765	5.632	0.003	0.066	0.001	0.135	0.012	0.240	0.003	4.699	0.012
Northern Cardinal	Cardinalis Cardinalis	134151	8.713	0.004	0.265	0.000	0.002	0.002	0.532	0.004	8.331	0.004
Northern Cardinal	Cardinalis Cardinalis	100764	5.762	0.006	0.064	0.001	0.101	0.008	0.285	0.007	4.829	0.013
Northern Cardinal	Cardinalis Cardinalis	94288	5.902	0.002	0.064	0.001	0.044	0.010	0.191	0.001	4.973	0.014
Northern Cardinal	Cardinalis Cardinalis	191165	9.034	0.000	0.206	0.000	0.311	0.001	0.393	0.000	8.897	0.002
Northern Cardinal	Cardinalis Cardinalis	100747	5.547	0.012	0.070	0.001	0.251	0.015	0.404	0.012	4.938	0.019
Northern Cardinal	Cardinalis Cardinalis	107279	5.845	0.007	0.071	0.002	0.162	0.011	0.160	0.002	5.055	0.003
Northern Cardinal	Cardinalis Cardinalis	107278	5.784	0.006	0.067	0.001	0.132	0.011	0.238	0.008	4.936	0.012
Northern Cardinal	Cardinalis Cardinalis	176241	6.078	0.002	0.058	0.000	0.088	0.003	0.412	0.003	5.309	0.006
NorthernMockingbird	Mimus Polyglottos	23403	5.512	0.010	0.065	0.001	0.157	0.013	0.434	0.012	4.760	0.020
NorthernMockingbird	Mimus Polyglottos	197002	9.206	0.003	0.378	0.000	0.021	0.002	0.145	0.001	8.162	0.006
NorthernMockingbird	Mimus Polyglottos	94375	5.756	0.003	0.073	0.001	0.086	0.003	0.220	0.004	4.757	0.010
NorthernMockingbird	Mimus Polyglottos	94372	5.609	0.005	70.747	0.000	0.138	0.007	0.366	0.003	4.795	0.007
NorthernMockingbird	Mimus Polyglottos	23402	5.817	0.000	0.068	0.000	0.001	0.000	0.090	0.000	4.763	0.001
NorthernMockingbird	Mimus Polyglottos	94373	5.820	0.008	0.064	0.000	0.039	0.006	0.306	0.002	4.861	0.007
NorthernMockingbird	Mimus Polyglottos	94374	5.908	0.001	0.065	0.001	0.011	0.004	0.262	0.003	4.984	0.001
NorthernMockingbird	Mimus Polyglottos	166628	6.072	0.001	0.059	0.001	0.038	0.003	0.048	0.000	5.042	0.001
NorthernMockingbird	Mimus Polyglottos	85196	5.991	0.000	0.062	0.000	0.020	0.001	0.256	0.003	5.220	0.001
NorthernMockingbird	Mimus Polyglottos	85198	5.849	0.005	0.063	0.001	0.050	0.007	0.267	0.005	4.860	0.005
NorthernMockingbird	Mimus Polyglottos	118628	5.849	0.003	0.170	0.002	0.012	0.003	0.116	0.004	4.811	0.008
NorthernMockingbird	Mimus Polyglottos	85197	5.931	0.002	0.063	0.000	0.056	0.004	0.132	0.001	4.852	0.005
NorthernMockingbird	Mimus Polyglottos	22932	5.867	0.004	0.057	0.001	0.104	0.004	0.189	0.004	4.929	0.007
NorthernMockingbird	Mimus Polyglottos	56847	5.979	0.001	0.059	0.000	0.021	0.002	0.109	0.000	4.902	0.003
NorthernMockingbird	Mimus Polyglottos	22933	5.615	0.006	0.069	0.001	0.113	0.009	0.212	0.007	4.602	0.010
NorthernMockingbird	Mimus Polyglottos	50223	5.788	0.003	0.059	0.001	0.065	0.006	0.127	0.001	4.805	0.003
NorthernMockingbird	Mimus Polyglottos	85192	5.905	0.003	0.445	0.002	0.090	0.005	0.132	0.004	4.951	0.007
NorthernMockingbird	Mimus Polyglottos	85193	5.906	0.003	0.266	0.002	0.076	0.005	0.131	0.004	4.918	0.007
NorthernMockingbird	Mimus Polyglottos	118613	5.876	0.004	0.057	0.000	0.089	0.002	0.222	0.003	4.999	0.004
NorthernMockingbird	Mimus Polyglottos	100752	5.799	0.003	0.070	0.000	0.050	0.002	0.289	0.005	4.872	0.004
Rose-breasted Grosbeak	Pheucticus Ludovicanius	84866	5.751	0.001	87.082	0.000	0.171	0.004	0.383	0.004	4.848	0.002
Rose-breasted Grosbeak	Pheucticus Ludovicanius	59210	6.035	0.001	0.066	0.000	0.170	0.005	0.518	0.002	5.331	0.001
Rose-breasted Grosbeak	Pheucticus Ludovicanius	94313	5.764	0.001	0.064	0.001	0.084	0.003	0.184	0.000	4.846	0.008

Table S4: Parameters for the ARMA model for each recording

Common Name	Species	Recording ID	*A*	σ_A	τ	σ_τ	*f*	σ_f	φ	σ_φ	κ	σ_κ
Rose-breasted Grosbeak	Pheucticus Ludovicanius	192210	8.920	0.005	68.823	0.003	0.034	0.004	0.199	0.006	7.718	0.017
Rose-breasted Grosbeak	Pheucticus Ludovicanius	191158	9.672	0.001	0.064	0.000	0.078	0.002	0.263	0.002	8.720	0.003
Rose-breasted Grosbeak	Pheucticus Ludovicanius	84864	5.739	0.001	0.069	0.000	0.269	0.001	0.362	0.000	5.158	0.002
Rose-breasted Grosbeak	Pheucticus Ludovicanius	113502	6.047	0.001	0.064	0.000	0.143	0.001	0.558	0.001	5.519	0.001
Rose-breasted Grosbeak	Pheucticus Ludovicanius	84865	5.998	0.000	0.064	0.001	0.019	0.001	0.122	0.000	5.154	0.002
Rose-breasted Grosbeak	Pheucticus Ludovicanius	176167	5.699	0.006	0.062	0.001	0.067	0.009	0.379	0.002	4.810	0.004
Rose-breasted Grosbeak	Pheucticus Ludovicanius	16980	5.651	0.006	0.066	0.001	0.184	0.015	0.438	0.007	4.857	0.013
Rose-breasted Grosbeak	Pheucticus Ludovicanius	59213	5.867	0.006	0.065	0.000	0.211	0.008	0.515	0.007	5.333	0.011
Rose-breasted Grosbeak	Pheucticus Ludovicanius	74906	5.680	0.008	88.930	0.065	0.075	0.011	0.388	0.010	4.795	0.017
Rose-breasted Grosbeak	Pheucticus Ludovicanius	49615	6.141	0.003	0.067	0.000	0.164	0.001	0.355	0.002	5.310	0.003
Rose-breasted Grosbeak	Pheucticus Ludovicanius	94327	5.737	0.001	0.062	0.001	0.056	0.002	0.147	0.001	5.009	0.002
Rose-breasted Grosbeak	Pheucticus Ludovicanius	53163	5.575	0.000	0.057	0.000	0.089	0.003	0.487	0.001	5.195	0.002
Rose-breasted Grosbeak	Pheucticus Ludovicanius	59206	5.777	0.004	0.068	0.000	0.296	0.002	0.209	0.001	5.111	0.004
Rose-breasted Grosbeak	Pheucticus Ludovicanius	191267	5.630	0.005	0.067	0.001	0.133	0.010	0.181	0.002	4.819	0.008
Rose-breasted Grosbeak	Pheucticus Ludovicanius	125225	8.854	0.004	0.062	0.000	0.057	0.003	0.168	0.002	7.903	0.002
Rose-breasted Grosbeak	Pheucticus Ludovicanius	50268	5.914	0.001	0.061	0.000	0.052	0.001	0.215	0.000	5.027	0.001
Rose-breasted Grosbeak	Pheucticus Ludovicanius	107970	5.769	0.006	93.094	0.001	0.044	0.006	0.380	0.008	4.717	0.011
Ryukyu Scops Owl	Otus elegans	247327	5.602	0.001	0.061	0.001	0.025	0.001	0.090	0.000	4.569	0.002
Ryukyu Scops Owl	Otus elegans	247329	5.592	0.004	0.073	0.001	0.095	0.005	0.204	0.005	4.605	0.009
Ryukyu Scops Owl	Otus elegans	247328	5.906	0.000	0.063	0.000	0.053	0.001	0.199	0.000	4.988	0.001
Ryukyu Scops Owl	Otus elegans	247326	5.668	0.004	0.071	0.001	0.143	0.006	0.288	0.006	4.753	0.009
Ryukyu Scops Owl	Otus elegans	247332	5.603	0.001	0.065	0.000	0.015	0.002	0.305	0.002	4.808	0.002
Ryukyu Scops Owl	Otus elegans	247335	5.529	0.002	0.071	0.001	0.002	0.001	0.149	0.002	4.452	0.007
Ryukyu Scops Owl	Otus elegans	247334	5.757	0.004	0.068	0.001	0.079	0.005	0.405	0.006	4.947	0.008
Ryukyu Scops Owl	Otus elegans	247333	5.804	0.004	22.727	0.015	0.036	0.003	-0.045	0.003	5.132	0.005
Ryukyu Scops Owl	Otus elegans	247341	5.513	0.001	0.070	0.000	0.068	0.000	0.294	0.001	4.958	0.001
Ryukyu Scops Owl	Otus elegans	247324	5.970	0.001	0.065	0.000	0.084	0.001	0.190	0.000	5.287	0.001
Ryukyu Scops Owl	Otus elegans	247323	5.794	0.001	0.067	0.000	0.028	0.001	0.177	0.003	4.677	0.006
Ryukyu Scops Owl	Otus elegans	247340	5.538	0.006	0.067	0.001	0.037	0.016	0.124	0.003	4.504	0.017
Ryukyu Scops Owl	Otus elegans	247322	6.145	0.000	0.070	0.000	0.014	0.000	0.308	0.000	5.124	0.001
Ryukyu Scops Owl	Otus elegans	247325	5.579	0.002	0.076	0.002	0.023	0.001	0.049	0.003	4.459	0.005
Ryukyu Scops Owl	Otus elegans	247338	5.732	0.002	0.067	0.000	0.078	0.004	0.423	0.004	4.978	0.007
Ryukyu Scops Owl	Otus elegans	247336	5.496	0.003	2.297	0.004	0.016	0.003	0.098	0.004	4.488	0.005
Ryukyu Scops Owl	Otus elegans	247331	5.748	0.004	0.069	0.001	0.118	0.006	0.348	0.005	4.866	0.010
Ryukyu Scops Owl	Otus elegans	247330	5.678	0.016	0.070	0.002	0.137	0.011	0.283	0.003	4.816	0.018
Ryukyu Scops Owl	Otus elegans	247337	5.606	0.006	0.069	0.001	0.207	0.007	0.321	0.007	4.894	0.011
Ryukyu Scops Owl	Otus elegans	247339	5.557	0.004	0.069	0.001	0.072	0.006	0.228	0.005	4.541	0.010
Swainson’s Thrush	Catharus Ustulatus	4240	5.824	0.003	0.066	0.000	0.102	0.001	0.170	0.001	5.064	0.002

Table S4: Parameters for the ARMA model for each recording

**Common Name**	**Species**	**Recording ID**	A	σ_A	τ	σ_τ	f	σ_f	φ	σ_φ	κ	σ_κ
Swainson’s Thrush	Catharus Ustulatus	93793	6.273	0.001	0.066	0.000	0.019	0.003	0.204	0.002	5.355	0.003
Swainson’s Thrush	Catharus Ustulatus	136190	5.924	0.000	0.068	0.000	0.086	0.009	0.378	0.006	5.395	0.004
Swainson’s Thrush	Catharus Ustulatus	93794	6.197	0.004	0.074	0.001	0.011	0.006	0.219	0.006	5.227	0.009
Swainson’s Thrush	Catharus Ustulatus	130939	9.045	0.005	99.875	0.001	0.005	0.003	0.196	0.005	8.010	0.015
Swainson’s Thrush	Catharus Ustulatus	119444	6.109	0.000	0.064	0.000	0.028	0.000	0.356	0.000	5.468	0.000
Swainson’s Thrush	Catharus Ustulatus	121953	6.043	0.004	0.066	0.000	0.084	0.006	0.435	0.005	5.278	0.008
Swainson’s Thrush	Catharus Ustulatus	118694	5.657	0.003	0.066	0.001	0.063	0.008	0.345	0.001	5.102	0.004
Swainson’s Thrush	Catharus Ustulatus	136156	5.911	0.001	0.072	0.000	0.052	0.001	0.211	0.001	4.899	0.003
Swainson’s Thrush	Catharus Ustulatus	4221	5.651	0.003	0.050	0.000	0.057	0.004	0.274	0.003	4.701	0.006
Swainson’s Thrush	Catharus Ustulatus	133353	5.895	0.004	0.066	0.001	0.028	0.005	0.243	0.002	4.954	0.005
Swainson’s Thrush	Catharus Ustulatus	126471	5.460	0.011	91.536	0.001	0.005	0.003	0.426	0.014	4.692	0.004
Swainson’s Thrush	Catharus Ustulatus	171721	9.189	0.004	0.053	0.003	0.002	0.002	0.032	0.001	8.015	0.008
Swainson’s Thrush	Catharus Ustulatus	131468	9.735	0.004	0.157	0.001	0.007	0.005	0.274	0.004	8.877	0.008
Swainson’s Thrush	Catharus Ustulatus	179528	9.356	0.004	0.052	0.001	0.001	0.001	0.326	0.005	8.476	0.006
Swainson’s Thrush	Catharus Ustulatus	188871	9.311	0.002	1.188	0.004	0.010	0.003	0.050	0.001	8.192	0.004
Swainson’s Thrush	Catharus Ustulatus	119446	5.767	0.001	0.063	0.000	0.024	0.000	0.282	0.000	5.273	0.001
Swainson’s Thrush	Catharus Ustulatus	119447	5.861	0.000	0.077	0.000	0.061	0.002	0.152	0.000	5.173	0.001
Swainson’s Thrush	Catharus Ustulatus	130994	9.276	0.003	0.165	0.001	0.020	0.000	0.182	0.001	8.487	0.004
Swainson’s Thrush	Catharus Ustulatus	4232	5.887	0.002	0.072	0.000	0.081	0.001	0.396	0.000	5.326	0.008
Swainson’s Thrush	Catharus Ustulatus	100877	5.637	0.004	0.067	0.001	0.034	0.003	0.169	0.001	4.812	0.003
Veery	Catharus Fuscescens	27193	5.953	0.005	0.067	0.000	0.066	0.006	0.533	0.005	5.255	0.009
Veery	Catharus Fuscescens	3663	5.984	0.000	0.074	0.000	0.106	0.001	0.471	0.000	5.396	0.001
Veery	Catharus Fuscescens	3648	5.863	0.005	0.070	0.001	0.114	0.007	0.416	0.006	5.173	0.011
Veery	Catharus Fuscescens	3646	5.854	0.001	0.065	0.000	0.095	0.001	0.508	0.000	5.941	0.000
Veery	Catharus Fuscescens	188824	9.153	0.001	0.072	0.000	0.074	0.001	0.217	0.000	8.721	0.001
Veery	Catharus Fuscescens	146568	5.721	0.001	0.067	0.000	0.022	0.000	0.254	0.000	4.898	0.001
Veery	Catharus Fuscescens	26770	5.815	0.001	0.069	0.000	0.143	0.003	0.531	0.001	5.318	0.001
Veery	Catharus Fuscescens	67777	5.845	0.003	0.068	0.000	0.072	0.004	0.433	0.006	5.062	0.008
Veery	Catharus Fuscescens	135727	5.565	0.000	0.072	0.000	0.031	0.000	0.212	0.001	4.685	0.001
Veery	Catharus Fuscescens	135720	5.896	0.001	0.070	0.000	0.085	0.004	0.465	0.001	5.116	0.002
Veery	Catharus Fuscescens	67782	5.774	0.002	0.068	0.000	0.152	0.001	0.400	0.001	5.442	0.001
Veery	Catharus Fuscescens	136577	5.849	0.003	0.068	0.000	0.071	0.005	0.442	0.004	5.283	0.001
Veery	Catharus Fuscescens	188826	9.390	0.000	0.069	0.000	0.094	0.001	0.328	0.001	8.596	0.000
Veery	Catharus Fuscescens	135714	5.821	0.000	0.069	0.000	0.103	0.000	0.204	0.000	4.910	0.001
Veery	Catharus Fuscescens	164574	8.804	0.003	0.061	0.000	0.061	0.001	0.456	0.001	8.011	0.001
Veery	Catharus Fuscescens	121915	5.918	0.001	0.060	0.000	0.130	0.000	0.424	0.003	5.459	0.005
White-throated Sparrow	Zonotrichia Albicollis	188878	8.881	0.000	0.061	0.000	0.075	0.001	0.641	0.000	9.394	0.001
White-throated Sparrow	Zonotrichia Albicollis	133396	5.850	0.001	0.062	0.001	0.032	0.001	0.150	0.000	4.813	0.010

Table S4: Parameters for the ARMA model for each recording

Common Name	Species	Recording ID	A	σ_A	τ	σ_τ	f	σ_f	φ	σ_φ	κ	σ_κ
White-throated Sparrow	Zonotrichia Albicollis	15594	5.880	0.004	0.074	0.001	0.171	0.009	0.431	0.008	5.378	0.003
White-throated Sparrow	Zonotrichia Albicollis	66745	5.859	0.001	0.065	0.000	0.241	0.001	0.405	0.000	5.228	0.001
White-throated Sparrow	Zonotrichia Albicollis	66742	5.800	0.001	0.066	0.000	0.070	0.001	0.454	0.003	5.317	0.000
White-throated Sparrow	Zonotrichia Albicollis	169036	8.873	0.001	0.054	0.001	0.019	0.003	0.207	0.002	7.746	0.004
White-throated Sparrow	Zonotrichia Albicollis	66744	5.695	0.003	0.066	0.001	0.084	0.002	0.217	0.002	4.921	0.005
White-throated Sparrow	Zonotrichia Albicollis	84685	5.920	0.003	0.065	0.001	0.063	0.002	0.234	0.003	4.916	0.005
White-throated Sparrow	Zonotrichia Albicollis	66766	5.667	0.000	0.071	0.000	0.204	0.000	0.449	0.002	5.364	0.001
White-throated Sparrow	Zonotrichia Albicollis	15603	5.751	0.002	0.066	0.001	0.105	0.001	0.290	0.000	5.460	0.002
White-throated Sparrow	Zonotrichia Albicollis	107292	5.898	0.003	0.062	0.000	0.096	0.003	0.279	0.002	5.112	0.001
White-throated Sparrow	Zonotrichia Albicollis	137668	5.840	0.003	48.540	24.242	0.005	0.008	0.030	0.007	4.688	0.006
White-throated Sparrow	Zonotrichia Albicollis	15605	5.771	0.001	0.064	0.000	0.049	0.000	0.266	0.000	5.150	0.001
White-throated Sparrow	Zonotrichia Albicollis	15586	5.777	0.004	0.063	0.001	0.024	0.003	0.039	0.000	4.741	0.010
White-throated Sparrow	Zonotrichia Albicollis	15562	5.673	0.003	0.069	0.000	0.066	0.008	0.316	0.007	4.688	0.009
White-throated Sparrow	Zonotrichia Albicollis	73979	5.867	0.001	0.058	0.000	0.076	0.000	0.455	0.001	5.290	0.000
White-throated Sparrow	Zonotrichia Albicollis	136579	6.068	0.003	0.061	0.000	0.123	0.004	0.357	0.002	5.224	0.016
White-throated Sparrow	Zonotrichia Albicollis	15601	5.970	0.006	0.068	0.000	0.169	0.004	0.462	0.003	5.408	0.003
White-throated Sparrow	Zonotrichia Albicollis	169021	9.791	0.006	0.073	0.000	0.185	0.012	0.579	0.006	9.174	0.008
Wolves	Canis Lupus	56761	5.813	0.003	0.069	0.000	0.302	0.001	0.547	0.002	5.423	0.003
Wolves	Canis Lupus	116310	5.837	0.001	0.071	0.000	0.083	0.002	0.224	0.000	4.902	0.001
Wolves	Canis Lupus	116373	5.942	0.003	0.069	0.001	0.141	0.007	0.067	0.000	5.356	0.003
Wolves	Canis Lupus	116387	5.702	0.000	0.066	0.000	0.042	0.001	0.143	0.000	4.664	0.000
Wolves	Canis Lupus	116309	5.825	0.002	0.069	0.000	0.047	0.004	0.297	0.003	4.816	0.006
Wolves	Canis Lupus	116385	5.594	0.001	0.070	0.000	0.047	0.001	0.180	0.000	4.811	0.000
Wolves	Canis Lupus	116382	6.375	0.000	0.067	0.000	0.060	0.000	0.127	0.000	5.569	0.000
Wolves	Canis Lupus	116383	6.404	0.000	0.062	0.000	0.046	0.001	0.132	0.000	5.359	0.001
Wood Thrush	HylocichlaMustelina	11342	5.650	0.012	75.985	0.001	0.014	0.007	0.358	0.014	4.817	0.034
Wood Thrush	HylocichlaMustelina	11316	6.123	0.001	0.070	0.001	0.002	0.001	0.276	0.002	5.354	0.002
Wood Thrush	HylocichlaMustelina	176115	5.564	0.009	63.111	0.001	0.015	0.004	0.512	0.016	4.926	0.006
Wood Thrush	HylocichlaMustelina	11317	5.633	0.012	0.095	0.001	0.001	0.001	0.512	0.012	5.180	0.018
Wood Thrush	HylocichlaMustelina	176200	6.002	0.001	0.066	0.000	0.017	0.000	0.489	0.001	5.651	0.000
Wood Thrush	HylocichlaMustelina	40807	5.985	0.001	0.065	0.000	0.106	0.002	0.388	0.001	5.174	0.002
Wood Thrush	HylocichlaMustelina	107333	5.640	0.010	0.066	0.001	0.004	0.004	0.360	0.011	4.796	0.020
Wood Thrush	HylocichlaMustelina	94312	5.851	0.004	0.069	0.001	0.014	0.004	0.342	0.005	4.992	0.006
Wood Thrush	HylocichlaMustelina	94325	5.960	0.004	0.064	0.000	0.002	0.001	0.413	0.008	5.248	0.011
Wood Thrush	HylocichlaMustelina	84903	5.879	0.008	0.063	0.000	0.008	0.002	0.289	0.004	4.960	0.005
Wood Thrush	HylocichlaMustelina	11346	6.069	0.002	0.060	0.001	0.044	0.001	0.155	0.001	5.255	0.006
Wood Thrush	HylocichlaMustelina	191174	9.577	0.001	0.053	0.000	0.018	0.000	0.313	0.000	8.591	0.004
Wood Thrush	HylocichlaMustelina	176144	5.943	0.002	0.063	0.000	0.025	0.001	0.500	0.001	5.368	0.002

Table S4: Parameters for the ARMA model for each recording

Common Name	Species	Recording ID	A	σ_A	τ	σ_τ	f	σ_f	φ	σ_φ	κ	σ_κ
Wood Thrush	HylocichlaMustelina	107324	5.912	0.001	0.065	0.000	0.099	0.001	0.434	0.002	5.467	0.001
Wood Thrush	HylocichlaMustelina	94417	5.689	0.006	0.062	0.000	0.055	0.001	0.383	0.005	5.155	0.004
Wood Thrush	HylocichlaMustelina	176259	5.701	0.008	0.064	0.001	0.003	0.002	0.472	0.007	4.940	0.009
Wood Thrush	HylocichlaMustelina	94416	5.854	0.005	0.068	0.000	0.051	0.004	0.250	0.000	5.175	0.001
Wood Thrush	HylocichlaMustelina	168331	9.242	0.001	90.976	0.000	0.077	0.001	0.393	0.001	8.510	0.001
Wood Thrush	HylocichlaMustelina	100890	5.816	0.003	0.058	0.000	0.015	0.000	0.171	0.000	5.106	0.002
Wood Thrush	HylocichlaMustelina	11308	6.033	0.000	0.081	0.000	0.027	0.000	0.148	0.000	5.139	0.001

Table S5: Parameters for the Fractal model for each recording

Common Name	Species	Recording ID	A	σ_A	β	σ_β	λ	σ_λ	κ	σ_κ
Adelie Penguin	Pygoscelis Adeliae	247294	5.500	0.017	0.579	0.014	1.225	0.034	4.652	0.005
Adelie Penguin	Pygoscelis Adeliae	247293	5.464	0.014	0.708	0.012	1.367	0.029	4.692	0.004
Adelie Penguin	Pygoscelis Adeliae	247295	6.201	0.008	0.569	0.008	0.390	0.019	4.934	0.006
Adelie Penguin	Pygoscelis Adeliae	247301	9.201	0.004	0.586	0.006	0.011	0.010	7.936	0.010
Adelie Penguin	Pygoscelis Adeliae	247300	5.978	0.009	0.693	0.009	0.485	0.016	4.790	0.009
Adelie Penguin	Pygoscelis Adeliae	247297	6.098	0.003	0.456	0.004	0.026	0.022	4.789	0.007
Adelie Penguin	Pygoscelis Adeliae	247299	5.862	0.015	0.489	0.015	0.453	0.038	4.636	0.008
Adelie Penguin	Pygoscelis Adeliae	247298	9.214	0.006	0.414	0.006	0.073	0.058	7.852	0.008
Adelie Penguin	Pygoscelis Adeliae	247296	9.195	0.018	0.792	0.014	1.463	0.039	8.466	0.006
Adelie Penguin	Pygoscelis Adeliae	247302	6.082	0.004	0.500	0.006	0.025	0.022	4.775	0.010
Adelie Penguin	Pygoscelis Adeliae	247303	5.910	0.021	0.606	0.013	0.408	0.053	4.651	0.007
Adelie Penguin	Pygoscelis Adeliae	247304	6.075	0.004	0.529	0.006	0.018	0.016	4.755	0.011
Altamira Oriole	Icterus Gularis	12515	5.530	0.015	0.840	0.013	0.824	0.023	4.550	0.009
Altamira Oriole	Icterus Gularis	105591	5.900	0.007	0.453	0.009	0.029	0.028	4.660	0.015
Altamira Oriole	Icterus Gularis	20382	5.439	0.004	0.948	0.004	1.064	0.008	4.570	0.004
Altamira Oriole	Icterus Gularis	140215	9.195	0.011	0.322	0.014	0.067	0.059	7.947	0.019
Altamira Oriole	Icterus Gularis	20792	5.782	0.012	0.424	0.010	0.202	0.044	4.506	0.014
Altamira Oriole	Icterus Gularis	12519	4.643	0.010	1.017	0.019	2.651	0.033	4.481	0.009
Altamira Oriole	Icterus Gularis	131218	9.193	0.011	0.349	0.016	0.049	0.047	8.174	0.020
Bach - Brandenburg Concerto 1 long			7.974	0.002	0.749	0.003	0.004	0.003	6.825	0.005
Bach - Brandenburg Concerto 1 long2			8.904	0.000	0.650	0.001	0.014	0.001	7.543	0.001
Baltimore Oriole	Icterus Galbula	105614	5.938	0.005	0.538	0.008	0.029	0.026	4.573	0.014
Baltimore Oriole	Icterus Galbula	105640	5.898	0.007	0.404	0.010	0.040	0.034	4.872	0.014
Baltimore Oriole	Icterus Galbula	12481	5.957	0.025	0.547	0.023	0.617	0.048	4.953	0.005
Baltimore Oriole	Icterus Galbula	164538	9.060	0.006	0.281	0.009	0.040	0.037	7.761	0.013

Table S5: Parameters for the Fractal model for each recording

**Common Name**	**Species**	**Recording ID**	***A***	σ_A	***ß***	σ_β	***λ***	σ_λ	***κ***	σ_κ
Baltimore Oriole	Icterus Galbula	163341	9.508	0.031	0.267	0.021	0.401	0.089	8.425	0.014
Baltimore Oriole	Icterus Galbula	94326	6.087	0.005	0.446	0.008	0.031	0.028	4.828	0.013
Baltimore Oriole	Icterus Galbula	195752	9.606	0.010	0.210	0.011	0.079	0.074	8.310	0.015
Baltimore Oriole	Icterus Galbula	105630	6.033	0.006	0.515	0.009	0.028	0.025	4.733	0.014
Baltimore Oriole	Icterus Galbula	105642	5.936	0.008	0.503	0.011	0.041	0.035	4.611	0.018
Baltimore Oriole	Icterus Galbula	112699	5.715	0.027	0.577	0.025	0.461	0.056	4.458	0.019
Baltimore Oriole	Icterus Galbula	112697	5.809	0.009	0.689	0.005	0.495	0.016	4.987	0.004
Baltimore Oriole	Icterus Galbula	105627	6.004	0.007	0.458	0.010	0.040	0.036	4.744	0.015
Baltimore Oriole	Icterus Galbula	112698	5.783	0.011	0.598	0.013	0.092	0.070	4.380	0.020
Baltimore Oriole	Icterus Galbula	12482	5.182	0.090	0.606	0.153	0.285	0.244	3.724	0.099
Baltimore Oriole	Icterus Galbula	12483	6.185	0.004	0.504	0.006	0.040	0.035	4.911	0.009
Baltimore Oriole	Icterus Galbula	110227	6.039	0.005	0.507	0.006	0.031	0.026	4.712	0.012
Baltimore Oriole	Icterus Galbula	192118	9.380	0.003	0.463	0.007	0.655	0.010	8.490	0.006
Baltimore Oriole	Icterus Galbula	113500	6.016	0.002	0.460	0.004	0.288	0.007	4.742	0.007
Baltimore Oriole	Icterus Galbula	113501	5.804	0.009	0.448	0.014	0.040	0.038	4.487	0.020
Baltimore Oriole	Icterus Galbula	192119	9.349	0.007	0.327	0.009	0.044	0.038	8.097	0.013
Barred Owl	Strix Varia	134150	9.766	0.002	0.440	0.003	0.012	0.011	8.454	0.006
Barred Owl	Strix Varia	135412	10.102	0.004	0.886	0.006	0.004	0.004	9.210	0.008
Barred Owl	Strix Varia	187078	9.979	0.001	0.611	0.002	0.004	0.003	8.659	0.003
Barred Owl	Strix Varia	128933	9.794	0.003	0.504	0.005	0.012	0.011	8.490	0.008
Barred Owl	Strix Varia	79462	5.789	0.028	0.449	0.026	0.675	0.058	4.725	0.010
Barred Owl	Strix Varia	125364	6.203	0.008	0.370	0.008	0.104	0.075	4.910	0.007
Barred Owl	Strix Varia	49708	6.032	0.005	0.624	0.006	0.027	0.024	4.799	0.011
Barred Owl	Strix Varia	4549	5.826	0.000	0.913	0.000	0.695	0.000	5.166	0.000
Barred Owl	Strix Varia	128926	9.831	0.002	0.455	0.003	0.014	0.012	8.555	0.005
Barred Owl	Strix Varia	4548	5.263	0.021	0.776	0.017	1.412	0.044	4.534	0.006
Barred Owl	Strix Varia	128931	8.547	0.006	0.562	0.019	0.812	0.023	7.561	0.008
Barred Owl	Strix Varia	191186	9.272	0.003	0.576	0.004	0.009	0.009	7.852	0.007
Barred Owl	Strix Varia	128930	9.916	0.002	0.547	0.003	0.008	0.007	8.600	0.006
Barred Owl	Strix Varia	36950	5.155	0.004	1.027	0.002	1.502	0.004	4.698	0.005
Barred Owl	Strix Varia	110209	6.213	0.012	0.437	0.011	0.197	0.062	4.900	0.006
Barred Owl	Strix Varia	195216	5.705	0.019	0.445	0.010	0.967	0.037	4.750	0.003
Barred Owl	Strix Varia	175898	6.115	0.004	0.704	0.009	0.603	0.009	5.136	0.006
Barred Owl	Strix Varia	163907	6.034	0.011	0.613	0.010	0.339	0.027	4.774	0.008
Barred Owl	Strix Varia	201156	9.773	0.005	0.324	0.006	0.047	0.042	8.440	0.009
Barred Owl	Strix Varia	105433	6.438	0.003	0.276	0.005	0.027	0.024	5.032	0.008
Barred Owl	Strix Varia	72743	5.312	0.021	0.768	0.017	0.994	0.036	4.334	0.010
Barred Owl	Strix Varia	128924	9.572	0.020	0.710	0.019	0.876	0.035	8.543	0.010

Table S5: Parameters for the Fractal model for each recording

**Common Name**	**Species**	**Recording ID**	***A***	σ_A	***ß***	$\sigma_ß$	λ	σ_λ	κ	σ_κ
Barred Owl	Strix Varia	134149	9.527	0.005	0.429	0.007	0.022	0.020	8.257	0.011
Barred Owl	Strix Varia	52320	5.718	0.001	0.905	0.002	1.016	0.001	5.151	0.000
Barred Owl	Strix Varia	128925	8.388	0.031	1.199	0.022	3.238	0.121	8.289	0.005
Brown Thrasher	Toxostoma Rufum	67309	6.173	0.004	0.365	0.005	0.025	0.024	4.876	0.008
Brown Thrasher	Toxostoma Rufum	148978	5.386	0.079	0.313	0.050	1.197	0.175	4.564	0.008
Brown Thrasher	Toxostoma Rufum	100774	5.993	0.003	0.429	0.004	0.032	0.028	4.654	0.007
Brown Thrasher	Toxostoma Rufum	67312	5.842	0.001	0.494	0.003	0.674	0.003	4.772	0.005
Brown Thrasher	Toxostoma Rufum	190985	5.394	0.007	1.021	0.002	1.590	0.012	4.888	0.003
Brown Thrasher	Toxostoma Rufum	164590	5.675	0.023	0.526	0.020	1.033	0.045	4.750	0.008
Brown Thrasher	Toxostoma Rufum	176256	6.296	0.004	0.495	0.006	0.013	0.011	5.086	0.009
Brown Thrasher	Toxostoma Rufum	11174	6.084	0.003	0.331	0.005	0.025	0.024	4.815	0.007
Brown Thrasher	Toxostoma Rufum	105379	6.124	0.004	0.234	0.006	0.042	0.038	4.890	0.007
Brown Thrasher	Toxostoma Rufum	11180	5.738	0.014	0.567	0.013	0.810	0.026	4.683	0.006
Brown Thrasher	Toxostoma Rufum	84769	5.610	0.002	0.729	0.004	0.940	0.002	4.698	0.001
Brown Thrasher	Toxostoma Rufum	11175	5.785	0.003	0.555	0.007	1.030	0.003	4.877	0.003
Brown Thrasher	Toxostoma Rufum	84766	5.906	0.001	0.601	0.002	0.925	0.002	5.018	0.002
Brown Thrasher	Toxostoma Rufum	84768	5.697	0.022	0.490	0.026	0.686	0.047	4.644	0.002
Brown Thrasher	Toxostoma Rufum	94279	6.328	0.004	0.420	0.005	0.018	0.017	5.051	0.009
Brown Thrasher	Toxostoma Rufum	100779	6.173	0.003	0.448	0.004	0.015	0.013	4.831	0.007
Brown Thrasher	Toxostoma Rufum	60091	6.066	0.003	0.428	0.005	0.023	0.020	4.794	0.007
Brown Thrasher	Toxostoma Rufum	181512	9.750	0.003	0.296	0.004	0.028	0.025	8.456	0.006
Brown Thrasher	Toxostoma Rufum	100754	6.128	0.002	0.347	0.003	0.025	0.021	4.826	0.006
Brown Thrasher	Toxostoma Rufum	176262	6.534	0.002	0.489	0.003	0.007	0.007	5.252	0.005
CanyonWren	CatherpesMexicanus	197170	8.736	0.026	0.807	0.025	1.284	0.048	8.108	0.011
CanyonWren	CatherpesMexicanus	120253	5.926	0.039	0.419	0.033	0.533	0.095	4.884	0.008
CanyonWren	CatherpesMexicanus	197171	9.396	0.049	0.371	0.041	0.296	0.190	8.346	0.013
CanyonWren	CatherpesMexicanus	197943	9.510	0.021	0.531	0.020	0.852	0.040	8.589	0.007
CanyonWren	CatherpesMexicanus	44687	9.991	0.007	0.445	0.010	0.034	0.030	9.186	0.012
CanyonWren	CatherpesMexicanus	105214	5.634	0.051	0.461	0.044	0.931	0.103	4.725	0.012
CanyonWren	CatherpesMexicanus	9043	5.814	0.022	0.640	0.020	0.914	0.040	4.912	0.010
CanyonWren	CatherpesMexicanus	9044	5.581	0.022	0.532	0.026	0.925	0.044	4.760	0.016
CanyonWren	CatherpesMexicanus	37733	5.125	0.061	0.457	0.050	0.926	0.121	4.155	0.018
CanyonWren	CatherpesMexicanus	105240	5.544	0.033	0.739	0.030	0.951	0.057	4.582	0.017
CanyonWren	CatherpesMexicanus	110950	5.839	0.024	0.566	0.023	0.327	0.067	4.732	0.012
CanyonWren	CatherpesMexicanus	148337	9.486	0.006	0.614	0.008	0.068	0.052	8.140	0.011
CanyonWren	CatherpesMexicanus	189253	8.948	0.035	0.816	0.032	1.226	0.063	8.071	0.016
CanyonWren	CatherpesMexicanus	148381	9.696	0.008	0.564	0.011	0.022	0.020	8.836	0.014
CanyonWren	CatherpesMexicanus	120251	6.284	0.019	0.335	0.015	0.181	0.115	5.034	0.007

Table S5: Parameters for the Fractal model for each recording

Common Name	Species	Recording ID	*A*	σ_A	*ß*	$\sigma_ß$	λ	σ_λ	κ	σ_κ
CanyonWren	CatherpesMexicanus	45080	5.602	0.022	0.805	0.023	0.592	0.035	4.527	0.015
CanyonWren	CatherpesMexicanus	120205	6.100	0.012	0.580	0.011	0.459	0.026	4.907	0.007
CanyonWren	CatherpesMexicanus	44685	9.383	0.012	0.488	0.015	0.045	0.040	8.604	0.019
CanyonWren	CatherpesMexicanus	163246	5.926	0.006	0.484	0.016	0.194	0.028	4.778	0.020
CanyonWren	CatherpesMexicanus	63220	5.765	0.015	0.650	0.015	0.587	0.028	4.551	0.010
CanyonWren	CatherpesMexicanus	63218	5.365	0.018	0.898	0.015	1.453	0.037	4.658	0.007
CanyonWren	CatherpesMexicanus	9041	5.568	0.022	0.934	0.019	1.159	0.039	4.795	0.010
CanyonWren	CatherpesMexicanus	63219	5.478	0.026	0.724	0.021	1.032	0.046	4.510	0.012
Common Loon	Gavia Immer	197042	9.984	0.002	0.617	0.003	0.008	0.007	8.727	0.005
Common Loon	Gavia Immer	72736	5.979	0.001	0.699	0.003	0.509	0.001	4.858	0.001
Common Loon	Gavia Immer	913	6.179	0.003	0.557	0.005	0.014	0.013	4.986	0.008
Common Loon	Gavia Immer	61479	6.056	0.003	0.634	0.004	0.029	0.025	4.765	0.008
Common Loon	Gavia Immer	61477	6.118	0.003	0.564	0.004	0.020	0.019	4.797	0.009
Common Loon	Gavia Immer	193551	6.082	0.032	0.404	0.025	1.167	0.068	5.221	0.006
Common Loon	Gavia Immer	72720	6.186	0.003	0.646	0.004	0.014	0.012	4.892	0.007
Common Loon	Gavia Immer	197036	9.427	0.000	1.030	0.000	0.678	0.000	8.839	0.000
Common Loon	Gavia Immer	927	6.357	0.002	0.535	0.003	0.010	0.009	5.031	0.005
Common Loon	Gavia Immer	918	5.976	0.004	0.451	0.005	0.054	0.043	4.667	0.008
Common Loon	Gavia Immer	916	6.098	0.004	0.598	0.005	0.014	0.012	4.911	0.009
EasternWood-Pewee	Contopus Virens	73930	6.128	0.003	0.610	0.004	0.020	0.019	4.802	0.008
EasternWood-Pewee	Contopus Virens	176147	6.201	0.003	0.638	0.004	0.008	0.007	4.961	0.007
EasternWood-Pewee	Contopus Virens	55546	6.066	0.008	0.487	0.008	0.094	0.063	4.764	0.008
EasternWood-Pewee	Contopus Virens	41163	5.642	0.020	0.448	0.019	0.524	0.045	4.465	0.009
EasternWood-Pewee	Contopus Virens	110260	5.973	0.004	0.506	0.006	0.028	0.024	4.614	0.010
EasternWood-Pewee	Contopus Virens	191222	9.801	0.004	0.516	0.006	0.015	0.013	8.662	0.009
EasternWood-Pewee	Contopus Virens	188886	9.635	0.004	0.467	0.006	0.017	0.015	8.375	0.010
EasternWood-Pewee	Contopus Virens	191199	9.725	0.001	0.712	0.001	0.162	0.005	8.812	0.002
EasternWood-Pewee	Contopus Virens	7392	6.142	0.004	0.634	0.006	0.012	0.011	4.894	0.011
EasternWood-Pewee	Contopus Virens	68577	5.868	0.005	0.451	0.008	0.108	0.032	4.547	0.007
EasternWood-Pewee	Contopus Virens	116518	8.986	0.004	0.413	0.006	0.018	0.015	7.687	0.010
EasternWood-Pewee	Contopus Virens	176204	6.159	0.004	0.554	0.007	0.013	0.011	4.957	0.010
EasternWood-Pewee	Contopus Virens	116519	9.381	0.004	0.417	0.006	0.018	0.015	8.103	0.009
EasternWood-Pewee	Contopus Virens	176281	6.112	0.004	0.579	0.007	0.013	0.011	4.962	0.010
EasternWood-Pewee	Contopus Virens	62943	6.330	0.000	0.593	0.000	0.072	0.000	5.488	0.000
EasternWood-Pewee	Contopus Virens	38530	5.966	0.003	0.482	0.005	0.016	0.016	4.590	0.009
EasternWood-Pewee	Contopus Virens	101800	6.183	0.003	0.485	0.004	0.018	0.016	4.875	0.007
EasternWood-Pewee	Contopus Virens	191166	9.110	0.002	0.751	0.003	0.007	0.006	7.783	0.007
Field Cricket	Gryllus	200359	5.745	0.019	0.369	0.020	0.563	0.035	4.716	0.010

Table S5: Parameters for the Fractal model for each recording

**Common Name**	**Species**	**Recording ID**	***A***	***σ_A***	***ß***	***σ_ß***	***λ***	***σ λ***	***κ***	***σ_κ***
Field Cricket	Gryllus	85154	5.800	0.016	0.211	0.016	0.104	0.097	5.025	0.015
Field Cricket	Gryllus	54805	5.300	0.025	0.666	0.022	0.958	0.045	4.299	0.011
Field Cricket	Gryllus	200846	5.839	0.006	0.346	0.008	0.395	0.012	4.732	0.002
Field Cricket	Gryllus	191240	9.927	0.010	0.517	0.015	0.020	0.017	9.252	0.016
Field Cricket	Gryllus	200801	5.865	0.007	0.299	0.010	0.053	0.047	4.575	0.014
Field Cricket	Gryllus	200710	5.847	0.007	0.369	0.009	0.049	0.042	4.547	0.014
Field Cricket	Gryllus	85329	5.905	0.005	0.411	0.008	0.026	0.023	4.780	0.011
Field Cricket	Gryllus	85185	5.635	0.039	0.461	0.030	0.983	0.078	4.699	0.008
Field Cricket	Gryllus	85328	5.914	0.008	0.420	0.011	0.042	0.016	5.034	0.010
Field Cricket	Gryllus	200796	5.790	0.007	0.346	0.009	0.056	0.049	4.495	0.015
Field Cricket	Gryllus	85187	5.022	0.335	0.680	0.238	2.502	1.190	4.806	0.014
Field Cricket	Gryllus	85189	6.096	0.023	0.213	0.014	0.148	0.133	5.023	0.013
Field Cricket	Gryllus	85186	5.644	0.039	0.314	0.028	1.076	0.085	4.875	0.016
Frog	Lithobates	71895	6.551	0.028	1.066	0.040	9.734	0.317	7.350	0.006
Frog	Lithobates	182118	6.063	0.003	0.471	0.004	0.045	0.040	4.696	0.006
Frog	Lithobates	163335	8.941	0.005	0.438	0.007	0.015	0.014	7.784	0.009
Frog	Lithobates	163334	8.872	0.004	0.302	0.006	0.031	0.029	7.586	0.009
Frog	Lithobates	182023	6.055	0.010	0.466	0.009	0.114	0.075	4.697	0.008
Frog	Lithobates	179100	5.376	0.005	0.498	0.015	1.236	0.008	4.555	0.005
Frog	Lithobates	138552	8.231	0.002	0.340	0.003	0.011	0.011	7.003	0.004
Frog	Lithobates	138394	9.568	0.001	0.327	0.001	0.005	0.005	8.448	0.002
Frog	Lithobates	138553	8.834	0.001	0.239	0.001	0.009	0.008	7.562	0.002
Frog	Lithobates	182026	5.787	0.013	0.504	0.012	0.651	0.025	4.664	0.006
Frog	Lithobates	136572	6.322	0.003	0.339	0.005	0.017	0.015	5.124	0.007
Gray Catbird	Dumetella Carolinensis	57999	5.945	0.016	0.363	0.012	0.365	0.049	4.694	0.006
Gray Catbird	Dumetella Carolinensis	107339	6.386	0.003	0.329	0.004	0.020	0.018	5.099	0.007
Gray Catbird	Dumetella Carolinensis	36955	6.304	0.003	0.274	0.004	0.031	0.026	5.027	0.006
Gray Catbird	Dumetella Carolinensis	11434	6.220	0.003	0.437	0.005	0.021	0.019	4.911	0.008
Gray Catbird	Dumetella Carolinensis	121989	5.774	0.001	0.526	0.002	1.103	0.002	4.948	0.001
Gray Catbird	Dumetella Carolinensis	22454	6.271	0.003	0.400	0.005	0.025	0.022	4.983	0.008
Gray Catbird	Dumetella Carolinensis	168306	9.229	0.001	0.254	0.002	0.012	0.011	7.955	0.003
Gray Catbird	Dumetella Carolinensis	121994	6.348	0.003	0.414	0.005	0.014	0.013	5.095	0.008
Gray Catbird	Dumetella Carolinensis	107340	6.401	0.003	0.285	0.004	0.022	0.019	5.130	0.005
Gray Catbird	Dumetella Carolinensis	176248	6.262	0.003	0.316	0.005	0.029	0.026	4.960	0.007
Gray Catbird	Dumetella Carolinensis	50269	6.162	0.010	0.409	0.002	0.182	0.035	4.949	0.004
Gray Catbird	Dumetella Carolinensis	191237	9.786	0.002	0.250	0.003	0.018	0.017	8.537	0.005
Gray Catbird	Dumetella Carolinensis	10211	5.165	0.001	1.180	0.006	3.015	0.013	5.179	0.001
Gray Catbird	Dumetella Carolinensis	10218	6.475	0.002	0.329	0.004	0.013	0.011	5.239	0.005

Table S5: Parameters for the Fractal model for each recording

Common Name	Species	Recording ID	*A*	σ_A	*ß*	$\sigma_ß$	*λ*	σ_λ	*κ*	σ_κ
Gray Catbird	Dumetella Carolinensis	94321	5.704	0.035	0.458	0.028	0.992	0.071	4.740	0.008
Gray Catbird	Dumetella Carolinensis	93763	6.457	0.003	0.320	0.004	0.021	0.017	5.176	0.006
Green-Rumped Parrotlet	Forpus Passerinus	247305	6.114	0.015	0.560	0.014	0.776	0.029	5.076	0.007
Green-Rumped Parrotlet	Forpus Passerinus	247321	5.661	0.004	0.538	0.014	1.068	0.008	4.779	0.009
Green-Rumped Parrotlet	Forpus Passerinus	247319	5.670	0.007	0.311	0.008	0.068	0.062	4.372	0.011
Green-Rumped Parrotlet	Forpus Passerinus	247310	5.645	0.020	0.502	0.018	0.600	0.041	4.496	0.009
Green-Rumped Parrotlet	Forpus Passerinus	247317	4.975	0.037	0.917	0.027	1.846	0.090	4.437	0.010
Green-Rumped Parrotlet	Forpus Passerinus	247316	4.614	0.046	0.812	0.035	2.412	0.144	4.266	0.010
Green-Rumped Parrotlet	Forpus Passerinus	247311	5.415	0.021	0.772	0.019	1.057	0.037	4.488	0.009
Green-Rumped Parrotlet	Forpus Passerinus	247318	5.211	0.017	0.510	0.014	1.089	0.035	4.322	0.008
Green-Rumped Parrotlet	Forpus Passerinus	247320	5.848	0.005	0.366	0.007	0.040	0.036	4.581	0.011
Green-Rumped Parrotlet	Forpus Passerinus	247306	4.687	0.041	1.033	0.028	2.614	0.130	4.406	0.008
Green-Rumped Parrotlet	Forpus Passerinus	247308	3.614	0.112	1.338	0.070	8.531	1.089	4.340	0.008
Green-Rumped Parrotlet	Forpus Passerinus	247309	5.133	0.035	0.586	0.028	1.385	0.075	4.375	0.009
Green-Rumped Parrotlet	Forpus Passerinus	247307	5.990	0.008	0.349	0.008	0.086	0.076	4.673	0.008
Green-Rumped Parrotlet	Forpus Passerinus	247314	4.611	0.086	0.655	0.064	2.484	0.270	4.261	0.010
Green-Rumped Parrotlet	Forpus Passerinus	247313	5.152	0.047	0.437	0.038	1.204	0.102	4.300	0.009
Green-Rumped Parrotlet	Forpus Passerinus	247312	4.861	0.054	0.634	0.044	1.871	0.138	4.307	0.011
Green-Rumped Parrotlet	Forpus Passerinus	247315	4.869	0.003	1.113	0.009	2.125	0.009	4.599	0.003
HumpbackWhale	Megaptera Novaeangliae	117297	6.392	0.001	0.561	0.002	0.007	0.006	5.142	0.003
HumpbackWhale	Megaptera Novaeangliae	247348	6.344	0.001	0.520	0.002	0.009	0.008	5.076	0.004
HumpbackWhale	Megaptera Novaeangliae	247346	5.612	0.000	0.673	0.003	1.405	0.001	4.895	0.001
HumpbackWhale	Megaptera Novaeangliae	247347	5.431	0.012	0.717	0.009	1.793	0.028	4.837	0.003
HumpbackWhale	Megaptera Novaeangliae	247349	5.205	0.002	0.969	0.002	2.181	0.008	4.770	0.000
HumpbackWhale	Megaptera Novaeangliae	110847	6.568	0.001	0.750	0.002	0.005	0.004	5.522	0.003
HumpbackWhale	Megaptera Novaeangliae	247350	5.219	0.083	0.241	0.043	1.887	0.226	4.692	0.004
HumpbackWhale	Megaptera Novaeangliae	247351	5.892	0.009	0.487	0.009	0.701	0.019	4.768	0.004
HumpbackWhale	Megaptera Novaeangliae	247345	6.267	0.006	0.421	0.005	0.209	0.026	4.980	0.003
HumpbackWhale	Megaptera Novaeangliae	118144	6.244	0.005	0.441	0.005	0.302	0.018	4.992	0.003
KillerWhale	Orcinus Orca	123105	6.027	0.006	0.551	0.006	0.567	0.012	4.851	0.003
KillerWhale	Orcinus Orca	123132	6.110	0.007	0.351	0.006	0.088	0.072	4.821	0.006
KillerWhale	Orcinus Orca	128204	6.179	0.000	0.704	0.001	0.058	0.000	5.015	0.000
KillerWhale	Orcinus Orca	128216	6.302	0.002	0.475	0.003	0.011	0.010	4.955	0.006
KillerWhale	Orcinus Orca	128218	5.904	0.001	0.648	0.002	0.389	0.001	4.923	0.000
KillerWhale	Orcinus Orca	128217	6.305	0.002	0.655	0.003	0.008	0.007	4.982	0.006
KillerWhale	Orcinus Orca	120761	9.408	0.002	0.354	0.003	0.008	0.007	8.144	0.004
KillerWhale	Orcinus Orca	123145	5.773	0.000	0.684	0.001	0.942	0.002	5.036	0.000
KillerWhale	Orcinus Orca	123142	6.140	0.016	0.403	0.014	0.338	0.051	4.934	0.006

Table S5: Parameters for the Fractal model for each recording

**Common Name**	**Species**	**Recording ID**	***A***	σ_A	***ß***	σ_{β}	λ	σ_{λ}	κ	σ_{κ}
KillerWhale	Orcinus Orca	123136	6.466	0.003	0.227	0.005	0.031	0.028	5.239	0.007
KillerWhale	Orcinus Orca	128215	6.120	0.002	0.786	0.000	0.324	0.003	5.198	0.001
KillerWhale	Orcinus Orca	128223	9.307	0.002	0.685	0.003	0.005	0.005	8.059	0.006
KillerWhale	Orcinus Orca	128222	9.000	0.002	0.469	0.003	0.012	0.011	7.642	0.005
KillerWhale	Orcinus Orca	128214	6.420	0.002	0.673	0.002	0.006	0.005	5.090	0.005
KillerWhale	Orcinus Orca	123125	5.956	0.025	0.393	0.020	0.747	0.054	4.879	0.007
KillerWhale	Orcinus Orca	120595	6.055	0.024	0.338	0.017	0.506	0.062	4.888	0.006
KillerWhale	Orcinus Orca	123124	5.917	0.017	0.424	0.015	0.499	0.040	4.731	0.007
Northern Cardinal	Cardinalis Cardinalis	94283	6.148	0.004	0.521	0.006	0.019	0.017	4.867	0.011
Northern Cardinal	Cardinalis Cardinalis	84683	5.992	0.004	0.414	0.006	0.025	0.022	4.723	0.010
Northern Cardinal	Cardinalis Cardinalis	98872	5.944	0.011	0.360	0.011	0.097	0.083	4.747	0.012
Northern Cardinal	Cardinalis Cardinalis	130905	6.270	0.004	0.348	0.005	0.036	0.030	5.098	0.007
Northern Cardinal	Cardinalis Cardinalis	100794	5.631	0.024	0.774	0.022	1.044	0.042	4.804	0.012
Northern Cardinal	Cardinalis Cardinalis	107306	6.169	0.005	0.556	0.006	0.029	0.025	5.020	0.010
Northern Cardinal	Cardinalis Cardinalis	100744	5.320	0.044	0.813	0.033	1.329	0.085	4.573	0.015
Northern Cardinal	Cardinalis Cardinalis	176244	5.712	0.009	0.834	0.009	0.521	0.026	4.997	0.010
Northern Cardinal	Cardinalis Cardinalis	57975	6.068	0.008	0.429	0.009	0.082	0.059	4.822	0.011
Northern Cardinal	Cardinalis Cardinalis	84706	5.890	0.009	0.266	0.012	0.070	0.058	4.692	0.015
Northern Cardinal	Cardinalis Cardinalis	49063	5.961	0.006	0.508	0.009	0.039	0.032	4.685	0.015
Northern Cardinal	Cardinalis Cardinalis	100765	5.807	0.018	0.418	0.017	0.131	0.107	4.531	0.019
Northern Cardinal	Cardinalis Cardinalis	134151	7.914	0.099	1.086	0.088	2.830	0.334	8.445	0.016
Northern Cardinal	Cardinalis Cardinalis	100764	5.595	0.003	0.659	0.009	0.865	0.006	4.808	0.002
Northern Cardinal	Cardinalis Cardinalis	94288	6.058	0.021	0.341	0.015	0.170	0.121	4.840	0.010
Northern Cardinal	Cardinalis Cardinalis	191165	8.775	0.050	1.021	0.051	1.525	0.098	8.784	0.016
Northern Cardinal	Cardinalis Cardinalis	100747	5.543	0.025	0.726	0.021	0.689	0.042	4.433	0.017
Northern Cardinal	Cardinalis Cardinalis	107279	5.594	0.009	0.772	0.009	1.077	0.016	4.839	0.003
Northern Cardinal	Cardinalis Cardinalis	107278	5.540	0.037	0.717	0.032	1.063	0.067	4.761	0.012
Northern Cardinal	Cardinalis Cardinalis	176241	6.311	0.003	0.498	0.004	0.034	0.031	5.100	0.007
NorthernMockingbird	Mimus Polyglottos	23403	5.737	0.010	0.519	0.011	0.076	0.061	4.411	0.018
NorthernMockingbird	Mimus Polyglottos	197002	8.857	0.048	0.578	0.040	1.300	0.099	8.109	0.011
NorthernMockingbird	Mimus Polyglottos	94375	5.617	0.023	0.596	0.021	0.854	0.042	4.577	0.010
NorthernMockingbird	Mimus Polyglottos	94372	5.771	0.010	0.558	0.011	0.314	0.015	4.467	0.015
NorthernMockingbird	Mimus Polyglottos	23402	5.649	0.025	0.369	0.018	0.892	0.052	4.685	0.005
NorthernMockingbird	Mimus Polyglottos	94373	6.012	0.004	0.423	0.006	0.031	0.029	4.671	0.011
NorthernMockingbird	Mimus Polyglottos	94374	6.086	0.004	0.357	0.005	0.040	0.036	4.782	0.008
NorthernMockingbird	Mimus Polyglottos	166628	5.859	0.003	0.437	0.007	0.990	0.007	4.949	0.006
NorthernMockingbird	Mimus Polyglottos	85196	6.223	0.005	0.363	0.007	0.031	0.026	4.961	0.011
NorthernMockingbird	Mimus Polyglottos	85198	6.012	0.005	0.361	0.007	0.044	0.040	4.712	0.011

Table S5: Parameters for the Fractal model for each recording

**Common Name**	**Species**	**Recording ID**	***A***	**σ_A**	**ß**	**$\sigma_ß$**	**λ**	**σ_λ**	**κ**	**σ_κ**
NorthernMockingbird	Mimus Polyglottos	118628	5.703	0.035	0.407	0.029	0.870	0.072	4.729	0.009
NorthernMockingbird	Mimus Polyglottos	85197	6.057	0.008	0.284	0.006	0.084	0.073	4.780	0.007
NorthernMockingbird	Mimus Polyglottos	22932	5.277	0.005	0.931	0.006	1.804	0.012	4.720	0.003
NorthernMockingbird	Mimus Polyglottos	56847	5.908	0.045	0.332	0.031	0.690	0.101	4.822	0.008
NorthernMockingbird	Mimus Polyglottos	22933	5.330	0.037	0.657	0.030	1.134	0.071	4.430	0.011
NorthernMockingbird	Mimus Polyglottos	50223	5.965	0.005	0.352	0.007	0.061	0.042	4.651	0.012
NorthernMockingbird	Mimus Polyglottos	85192	4.886	0.041	1.164	0.029	3.145	0.153	4.756	0.007
NorthernMockingbird	Mimus Polyglottos	85193	5.146	0.032	0.962	0.025	2.269	0.091	4.762	0.007
NorthernMockingbird	Mimus Polyglottos	118613	6.064	0.006	0.339	0.007	0.059	0.053	4.749	0.011
NorthernMockingbird	Mimus Polyglottos	100752	5.878	0.023	0.446	0.019	0.434	0.059	4.633	0.011
Rose-breasted Grosbeak	Pheucticus Ludovicanius	84866	5.963	0.008	0.495	0.011	0.028	0.025	4.833	0.016
Rose-breasted Grosbeak	Pheucticus Ludovicanius	59210	6.272	0.001	0.645	0.002	0.168	0.001	5.115	0.001
Rose-breasted Grosbeak	Pheucticus Ludovicanius	94313	5.968	0.007	0.423	0.010	0.049	0.045	4.669	0.016
Rose-breasted Grosbeak	Pheucticus Ludovicanius	192210	9.055	0.009	0.287	0.011	0.073	0.068	7.650	0.018
Rose-breasted Grosbeak	Pheucticus Ludovicanius	191158	9.840	0.003	0.357	0.004	0.040	0.036	8.645	0.006
Rose-breasted Grosbeak	Pheucticus Ludovicanius	84864	6.051	0.005	0.604	0.007	0.025	0.024	4.735	0.014
Rose-breasted Grosbeak	Pheucticus Ludovicanius	113502	6.340	0.004	0.531	0.005	0.012	0.010	5.220	0.008
Rose-breasted Grosbeak	Pheucticus Ludovicanius	84865	6.206	0.004	0.471	0.007	0.013	0.012	5.082	0.009
Rose-breasted Grosbeak	Pheucticus Ludovicanius	176167	5.940	0.007	0.484	0.010	0.027	0.024	4.663	0.016
Rose-breasted Grosbeak	Pheucticus Ludovicanius	16980	5.897	0.008	0.510	0.010	0.032	0.029	4.679	0.017
Rose-breasted Grosbeak	Pheucticus Ludovicanius	59213	6.007	0.001	0.761	0.005	0.423	0.002	5.008	0.002
Rose-breasted Grosbeak	Pheucticus Ludovicanius	74906	5.883	0.007	0.477	0.009	0.042	0.039	4.606	0.016
Rose-breasted Grosbeak	Pheucticus Ludovicanius	49615	6.349	0.004	0.486	0.005	0.045	0.037	5.122	0.007
Rose-breasted Grosbeak	Pheucticus Ludovicanius	94327	5.977	0.007	0.459	0.011	0.027	0.024	4.786	0.015
Rose-breasted Grosbeak	Pheucticus Ludovicanius	53163	6.007	0.006	0.523	0.008	0.021	0.018	4.752	0.014
Rose-breasted Grosbeak	Pheucticus Ludovicanius	59206	5.941	0.018	0.620	0.017	0.377	0.042	4.725	0.012
Rose-breasted Grosbeak	Pheucticus Ludovicanius	191267	5.716	0.056	0.513	0.033	0.492	0.153	4.639	0.020
Rose-breasted Grosbeak	Pheucticus Ludovicanius	125225	8.892	0.013	0.414	0.010	0.542	0.025	7.724	0.014
Rose-breasted Grosbeak	Pheucticus Ludovicanius	50268	6.100	0.004	0.388	0.006	0.024	0.022	4.906	0.009
Rose-breasted Grosbeak	Pheucticus Ludovicanius	107970	5.964	0.006	0.456	0.009	0.028	0.024	4.650	0.015
Ryukyu Scops Owl	Otus elegans	247327	5.733	0.007	0.272	0.007	0.081	0.070	4.485	0.008
Ryukyu Scops Owl	Otus elegans	247329	5.182	0.029	0.765	0.023	1.375	0.060	4.427	0.008
Ryukyu Scops Owl	Otus elegans	247328	6.064	0.003	0.470	0.005	0.018	0.017	4.737	0.009
Ryukyu Scops Owl	Otus elegans	247326	5.521	0.016	0.682	0.014	0.885	0.028	4.472	0.008
Ryukyu Scops Owl	Otus elegans	247332	5.817	0.004	0.395	0.005	0.026	0.024	4.532	0.009
Ryukyu Scops Owl	Otus elegans	247335	5.632	0.009	0.257	0.009	0.093	0.079	4.430	0.010
Ryukyu Scops Owl	Otus elegans	247334	5.973	0.004	0.507	0.005	0.031	0.028	4.632	0.009
Ryukyu Scops Owl	Otus elegans	247333	6.022	0.004	0.479	0.005	0.056	0.016	4.659	0.007

Table S5: Parameters for the Fractal model for each recording

Common Name	Species	Recording ID	A	σ_A	$ß$	$\sigma_ß$	λ	σ_λ	κ	σ_κ
Ryukyu Scops Owl	Otus elegans	247341	5.820	0.004	0.484	0.006	0.031	0.028	4.489	0.010
Ryukyu Scops Owl	Otus elegans	247324	6.210	0.002	0.508	0.003	0.012	0.010	4.874	0.006
Ryukyu Scops Owl	Otus elegans	247323	5.481	0.004	0.588	0.002	1.161	0.004	4.649	0.002
Ryukyu Scops Owl	Otus elegans	247340	5.685	0.007	0.349	0.009	0.080	0.069	4.382	0.010
Ryukyu Scops Owl	Otus elegans	247322	6.316	0.001	0.423	0.002	0.012	0.011	5.000	0.003
Ryukyu Scops Owl	Otus elegans	247325	4.537	0.066	0.886	0.046	3.193	0.255	4.408	0.007
Ryukyu Scops Owl	Otus elegans	247338	5.971	0.004	0.519	0.005	0.029	0.025	4.653	0.010
Ryukyu Scops Owl	Otus elegans	247336	5.591	0.009	0.202	0.009	0.081	0.077	4.458	0.009
Ryukyu Scops Owl	Otus elegans	247331	5.899	0.014	0.507	0.013	0.293	0.044	4.579	0.009
Ryukyu Scops Owl	Otus elegans	247330	5.750	0.013	0.572	0.013	0.462	0.028	4.525	0.008
Ryukyu Scops Owl	Otus elegans	247337	5.310	0.024	0.894	0.019	1.171	0.043	4.478	0.010
Ryukyu Scops Owl	Otus elegans	247339	5.273	0.005	0.698	0.008	1.121	0.007	4.341	0.008
Swainson’s Thrush	Catharus Ustulatus	4240	6.044	0.005	0.451	0.007	0.042	0.037	4.717	0.012
Swainson’s Thrush	Catharus Ustulatus	93793	6.263	0.017	0.493	0.017	0.664	0.037	5.174	0.007
Swainson’s Thrush	Catharus Ustulatus	136190	6.242	0.012	0.477	0.012	0.126	0.083	4.966	0.009
Swainson’s Thrush	Catharus Ustulatus	93794	5.993	0.008	0.670	0.006	0.968	0.012	5.112	0.003
Swainson’s Thrush	Catharus Ustulatus	130939	8.639	0.003	0.719	0.016	1.370	0.009	7.933	0.015
Swainson’s Thrush	Catharus Ustulatus	119444	6.337	0.002	0.483	0.004	0.017	0.015	5.050	0.006
Swainson’s Thrush	Catharus Ustulatus	121953	6.273	0.003	0.554	0.005	0.027	0.024	5.005	0.008
Swainson’s Thrush	Catharus Ustulatus	118694	5.919	0.007	0.602	0.009	0.036	0.031	4.641	0.016
Swainson’s Thrush	Catharus Ustulatus	136156	5.950	0.022	0.442	0.020	0.497	0.051	4.769	0.009
Swainson’s Thrush	Catharus Ustulatus	4221	5.841	0.006	0.324	0.007	0.048	0.041	4.685	0.010
Swainson’s Thrush	Catharus Ustulatus	133353	5.976	0.023	0.484	0.023	0.430	0.059	4.793	0.010
Swainson’s Thrush	Catharus Ustulatus	126471	5.743	0.009	0.500	0.014	0.032	0.027	4.484	0.022
Swainson’s Thrush	Catharus Ustulatus	171721	9.113	0.073	0.097	0.018	0.552	0.186	8.013	0.010
Swainson’s Thrush	Catharus Ustulatus	131468	9.460	0.005	0.806	0.008	1.166	0.009	8.830	0.003
Swainson’s Thrush	Catharus Ustulatus	179528	9.576	0.004	0.416	0.005	0.032	0.030	8.349	0.008
Swainson’s Thrush	Catharus Ustulatus	188871	7.564	0.698	0.590	0.415	7.434	3.976	8.212	0.007
Swainson’s Thrush	Catharus Ustulatus	119446	6.078	0.004	0.535	0.006	0.018	0.017	4.810	0.010
Swainson’s Thrush	Catharus Ustulatus	119447	5.654	0.001	0.841	0.002	1.072	0.001	4.860	0.005
Swainson’s Thrush	Catharus Ustulatus	130994	9.359	0.043	0.428	0.040	0.498	0.106	8.376	0.012
Swainson’s Thrush	Catharus Ustulatus	4232	6.130	0.006	0.617	0.008	0.044	0.039	5.023	0.012
Swainson’s Thrush	Catharus Ustulatus	100877	5.820	0.011	0.406	0.012	0.086	0.079	4.576	0.013
Veery	Catharus Fuscescens	27193	6.215	0.003	0.648	0.005	0.011	0.010	4.953	0.009
Veery	Catharus Fuscescens	3663	6.258	0.003	0.657	0.004	0.010	0.009	5.040	0.008
Veery	Catharus Fuscescens	3648	5.989	0.012	0.637	0.012	0.407	0.025	4.788	0.009
Veery	Catharus Fuscescens	3646	6.325	0.002	0.670	0.006	0.107	0.021	5.159	0.013
Veery	Catharus Fuscescens	188824	9.450	0.005	0.549	0.007	0.026	0.022	8.317	0.010

Table S5: Parameters for the Fractal model for each recording

**Common Name**	**Species**	**Recording ID**	***A***	***σ_A***	***ß***	***σ_ß***	***λ***	***σ_λ***	***κ***	***σ_κ***
Veery	Catharus Fuscescens	146568	5.929	0.004	0.486	0.005	0.022	0.020	4.631	0.009
Veery	Catharus Fuscescens	26770	5.923	0.001	0.803	0.001	0.508	0.001	5.002	0.001
Veery	Catharus Fuscescens	67777	6.071	0.004	0.557	0.005	0.025	0.023	4.778	0.008
Veery	Catharus Fuscescens	135727	4.951	0.001	1.010	0.003	1.787	0.006	4.727	0.001
Veery	Catharus Fuscescens	135720	6.144	0.003	0.553	0.004	0.016	0.012	4.779	0.009
Veery	Catharus Fuscescens	67782	6.018	0.011	0.618	0.013	0.326	0.029	4.906	0.008
Veery	Catharus Fuscescens	136577	6.141	0.003	0.530	0.005	0.020	0.018	4.823	0.008
Veery	Catharus Fuscescens	188826	9.611	0.004	0.538	0.006	0.016	0.015	8.326	0.010
Veery	Catharus Fuscescens	135714	5.988	0.004	0.427	0.005	0.047	0.041	4.643	0.008
Veery	Catharus Fuscescens	164574	9.086	0.003	0.532	0.004	0.011	0.010	7.734	0.008
Veery	Catharus Fuscescens	121915	6.316	0.003	0.545	0.004	0.009	0.008	5.040	0.007
White-throated Sparrow	Zonotrichia Albicollis	188878	9.663	0.004	0.645	0.007	0.009	0.008	8.589	0.010
White-throated Sparrow	Zonotrichia Albicollis	133396	6.027	0.006	0.419	0.007	0.098	0.018	4.703	0.013
White-throated Sparrow	Zonotrichia Albicollis	15594	6.180	0.004	0.590	0.005	0.031	0.026	4.934	0.009
White-throated Sparrow	Zonotrichia Albicollis	66745	6.121	0.003	0.606	0.004	0.021	0.019	4.791	0.008
White-throated Sparrow	Zonotrichia Albicollis	66742	6.051	0.005	0.555	0.006	0.064	0.051	4.756	0.007
White-throated Sparrow	Zonotrichia Albicollis	169036	9.036	0.004	0.303	0.006	0.034	0.031	7.720	0.009
White-throated Sparrow	Zonotrichia Albicollis	66744	5.847	0.002	0.534	0.007	0.264	0.018	4.576	0.011
White-throated Sparrow	Zonotrichia Albicollis	84685	5.931	0.021	0.475	0.018	0.563	0.044	4.733	0.009
White-throated Sparrow	Zonotrichia Albicollis	66766	5.989	0.011	0.626	0.012	0.279	0.030	4.729	0.008
White-throated Sparrow	Zonotrichia Albicollis	15603	6.070	0.004	0.650	0.005	0.015	0.014	4.797	0.010
White-throated Sparrow	Zonotrichia Albicollis	107292	6.126	0.004	0.409	0.005	0.043	0.038	4.786	0.009
White-throated Sparrow	Zonotrichia Albicollis	137668	5.423	0.103	0.318	0.068	1.379	0.235	4.677	0.010
White-throated Sparrow	Zonotrichia Albicollis	15605	6.020	0.005	0.537	0.007	0.015	0.013	4.819	0.011
White-throated Sparrow	Zonotrichia Albicollis	15586	5.913	0.004	0.281	0.006	0.027	0.024	4.676	0.008
White-throated Sparrow	Zonotrichia Albicollis	15562	5.848	0.008	0.439	0.009	0.089	0.071	4.512	0.011
White-throated Sparrow	Zonotrichia Albicollis	73979	6.208	0.004	0.499	0.007	0.015	0.013	5.023	0.010
White-throated Sparrow	Zonotrichia Albicollis	136579	6.290	0.003	0.499	0.005	0.023	0.021	5.028	0.008
White-throated Sparrow	Zonotrichia Albicollis	15601	6.262	0.004	0.571	0.006	0.024	0.022	5.007	0.010
White-throated Sparrow	Zonotrichia Albicollis	169021	10.077	0.004	0.643	0.005	0.012	0.011	8.897	0.008
Wolves Canis	Lupus	56761	6.084	0.007	0.736	0.007	0.240	0.019	4.765	0.008
Wolves Canis	Lupus	116310	5.866	0.011	0.487	0.010	0.540	0.025	4.678	0.005
Wolves Canis	Lupus	116373	6.165	0.004	0.557	0.006	0.015	0.014	4.814	0.010
Wolves Canis	Lupus	116387	5.763	0.019	0.383	0.016	0.420	0.054	4.524	0.007
Wolves Canis	Lupus	116309	5.994	0.003	0.410	0.004	0.035	0.030	4.664	0.006
Wolves Canis	Lupus	116385	5.788	0.006	0.422	0.007	0.050	0.041	4.466	0.012
Wolves Canis	Lupus	116382	6.607	0.002	0.406	0.002	0.009	0.008	5.291	0.004
Wolves Canis	Lupus	116383	6.348	0.000	0.504	0.000	0.677	0.001	5.281	0.001

Table S5: Parameters for the Fractal model for each recording

**Common Name**	**Species**	**Recording ID**	A	σ_A	β	σ_β	λ	σ_λ	κ	σ_κ
Wood Thrush	HylocichlaMustelina	11342	5.846	0.017	0.521	0.022	0.114	0.097	4.610	0.025
Wood Thrush	HylocichlaMustelina	11316	6.269	0.014	0.463	0.015	0.317	0.045	5.106	0.008
Wood Thrush	HylocichlaMustelina	176115	5.862	0.008	0.574	0.011	0.031	0.023	4.615	0.019
Wood Thrush	HylocichlaMustelina	11317	5.934	0.017	0.616	0.024	0.143	0.086	5.065	0.017
Wood Thrush	HylocichlaMustelina	176200	6.296	0.004	0.683	0.005	0.015	0.015	5.303	0.008
Wood Thrush	HylocichlaMustelina	40807	6.207	0.003	0.541	0.004	0.016	0.015	4.857	0.007
Wood Thrush	HylocichlaMustelina	107333	5.839	0.008	0.494	0.012	0.053	0.045	4.595	0.018
Wood Thrush	HylocichlaMustelina	94312	6.047	0.007	0.499	0.009	0.064	0.056	4.774	0.012
Wood Thrush	HylocichlaMustelina	94325	6.202	0.005	0.573	0.007	0.022	0.020	5.081	0.012
Wood Thrush	HylocichlaMustelina	84903	6.065	0.006	0.483	0.009	0.026	0.023	4.873	0.013
Wood Thrush	HylocichlaMustelina	11346	6.247	0.004	0.428	0.002	0.083	0.035	5.076	0.007
Wood Thrush	HylocichlaMustelina	191174	9.805	0.003	0.404	0.005	0.017	0.014	8.568	0.007
Wood Thrush	HylocichlaMustelina	176144	6.223	0.004	0.649	0.006	0.011	0.010	5.134	0.009
Wood Thrush	HylocichlaMustelina	107324	6.118	0.006	0.625	0.008	0.023	0.021	5.132	0.012
Wood Thrush	HylocichlaMustelina	94417	5.980	0.006	0.530	0.008	0.049	0.044	4.683	0.014
Wood Thrush	HylocichlaMustelina	176259	5.948	0.006	0.606	0.009	0.024	0.021	4.737	0.015
Wood Thrush	HylocichlaMustelina	94416	6.076	0.005	0.490	0.007	0.028	0.023	4.831	0.010
Wood Thrush	HylocichlaMustelina	168331	9.457	0.007	0.545	0.011	0.023	0.021	8.304	0.017
Wood Thrush	HylocichlaMustelina	100890	6.057	0.005	0.471	0.007	0.026	0.023	4.764	0.012
Wood Thrush	HylocichlaMustelina	11308	6.194	0.003	0.391	0.005	0.029	0.026	5.033	0.006

Table S6: The left-hand side of equation 12 is shown for each species. Values near unity suggest that there is no intrinsic variation in the properties of recordings for a given species, whereas larger values indicate that such variation is present.

Species	Equation 12
Adelie Penguin	137.896
Altamira Oriole	928.313
Bach	935.399
Baltimore Oriole	172.962
Barred Owl	5688.23
Brown Thrasher	4408.72
CanyonWren	75.687
Common Loon	13042.1
EasternWood-Pewee	2055.56
Field Cricket	45.8298
Frog	521.839
Gray Catbird	2222.87
Green-Rumped Parrotlet	380.584
HumpbackWhale	2984.97
KillerWhale	4223.74
Northern Cardinal	247.983
NorthernMockingbird	428.604
Rose-breasted Grosbeak	461.733
Ryukyu Scops Owl	391.498
Swainson’s Thrush	835.52
Veery	1741.49
White-throated Sparrow	309.642
Wolves	508.682
Wood Thrush	210.109

Table S7: The $\log_{10}$ of the two-sided *p*-value for the Kolmogorov–Smirnov test versus a normal distribution is shown for each species. In the case of the Northern Mockingbird and Swainson’s Thrush the test produced a value below floating point precision and so *p* is effectively zero.

Species	$\log_{10}p$
Adelie Penguin	-3.68
Altamira Oriole	-1.96
Bach	-0.3
Baltimore Oriole	-4.11
Barred Owl	-16.99
Brown Thrasher	-8.48
CanyonWren	-3.72
Common Loon	-11.15
EasternWood-Pewee	-9.33
Field Cricket	-2.12
Frog	-7.82
Gray Catbird	-3.45

Table S7: The $\log_{10}$ of the two-sided *p*-value for the Kolmogorov–Smirnov test versus a normal distribution is shown for each species. In the case of the Northern Mockingbird and Swainson's Thrush the test produced a value below floating point precision and so *p* is effectively zero.

Species	$\log_{10}p$
Green-Rumped Parrotlet	-2.14
HumpbackWhale	-3.24
KillerWhale	-20.62
Northern Cardinal	-4.4
NorthernMockingbird	-2.46
Rose-breasted Grosbeak	-13.54
Ryukyu Scops Owl	-5.41
Swainson's Thrush	-11.27
Veery	-10.91
White-throated Sparrow	-3.43
Wolves	-2.82
Wood Thrush	-5.48

Table S8: The logarithm of the Bayesian evidence for the model given by equation 13 is shown for each species for $N \in \{1 \ldots 5\}$. The number of modes which maximises the evidence is shown in the penultimate column. The final column shows the difference between the preferred number of modes and the next-best number.

Species, No. Modes	1	2	3	4	5	Best	Difference
Adelie Penguin	-8.19	-9.58	-11.8	-12.42	-16.79	1	-1.39
Altamira Oriole	-7.99	-9.27	-11.15	-13.05	-15.65	1	-1.28
Bach	-2.82	-3.97	-5.71	-7.34	-7.3	1	-1.15
Baltimore Oriole	-11.65	-13.22	-15.31	-17.88	-20.45	1	-1.56
Barred Owl	-19.85	-21.39	-23.69	-26.12	-28.62	1	-1.54
Brown Thrasher	-14.48	-15.91	-17.96	-20.17	-22.83	1	-1.43
CanyonWren	-15.83	-17.42	-19.16	-22.0	-24.65	1	-1.58
Common Loon	-8.91	-10.51	-12.47	-14.52	-17.37	1	-1.61
EasternWood-Pewee	-9.04	-11.06	-13.19	-15.65	-18.33	1	-2.02
Field Cricket	-9.69	-11.3	-13.21	-15.73	-18.06	1	-1.61
Frog	-10.21	-11.45	-13.43	-15.57	-18.11	1	-1.25
Gray Catbird	-13.48	-13.77	-15.0	-17.91	-20.29	1	-0.29
Green-Rumped Parrotlet	-15.94	-17.54	-19.45	-21.89	-24.04	1	-1.6
HumpbackWhale	-9.44	-10.62	-12.53	-14.94	-17.43	1	-1.18
KillerWhale	-12.37	-14.05	-16.21	-17.67	-21.02	1	-1.67
Northern Cardinal	-16.25	-18.13	-20.21	-22.73	-25.35	1	-1.87
NorthernMockingbird	-17.01	-18.45	-20.46	-22.74	-25.44	1	-1.44
Rose-breasted Grosbeak	-10.55	-12.25	-14.03	-16.87	-19.19	1	-1.7
Ryukyu Scops Owl	-15.11	-16.72	-18.75	-21.06	-23.87	1	-1.61
Swainson's Thrush	-14.22	-16.28	-18.39	-20.78	-22.97	1	-2.06
Veery	-10.74	-12.29	-14.33	-16.77	-19.31	1	-1.55
White-throated Sparrow	-10.19	-12.48	-14.57	-16.61	-19.7	1	-2.29
Wolves	-6.82	-8.06	-10.01	-12.35	-14.89	1	-1.24
Wood Thrush	-8.38	-9.4	-11.56	-14.71	-17.29	1	-1.01

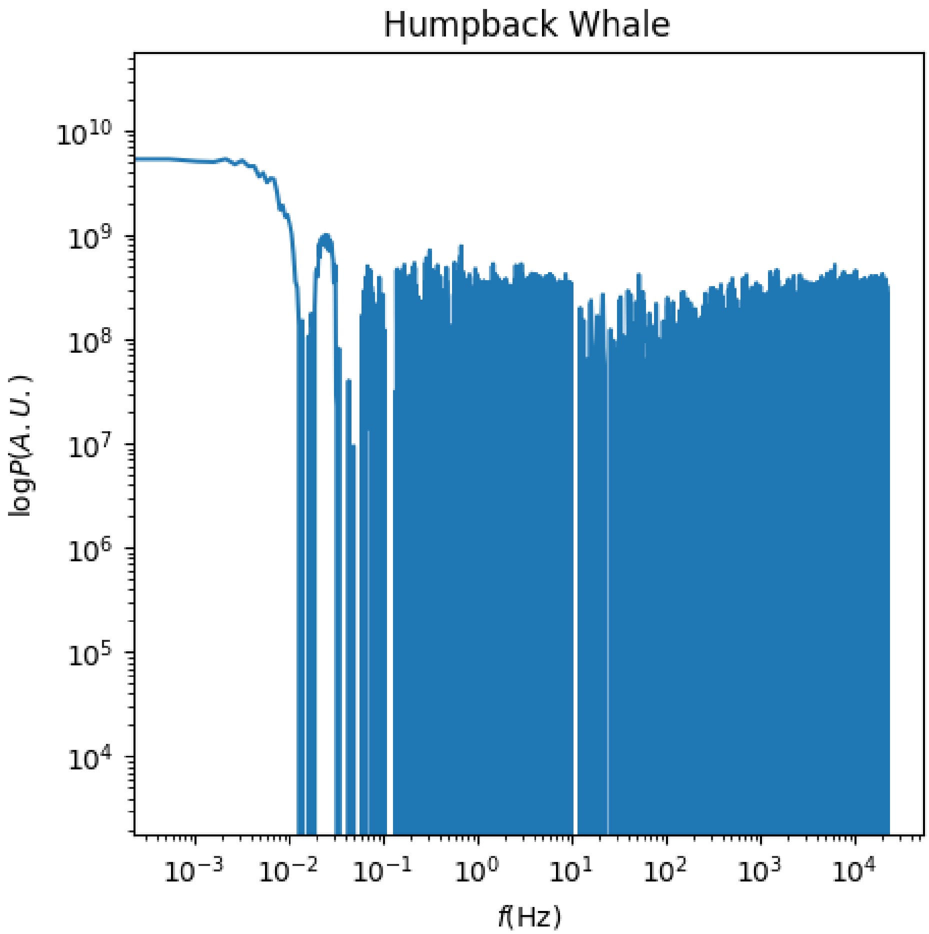

Figure S1: The power spectral density for Recording 247349 of the Humpback Whale is shown. Power is in arbitrary units to emphasise that the absolute volume is not meaningful in this analysis.

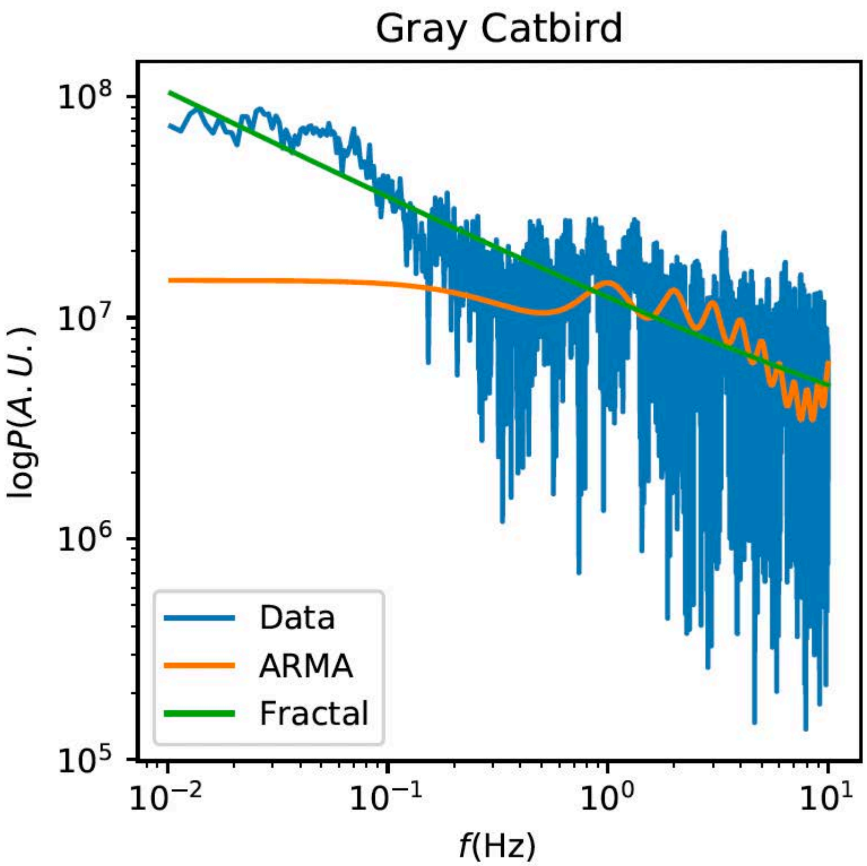

Figure S2. The spectrum of Recording 10218 of the Gray Catbird is shown along with the best-fit ARMA and fractal spectral models.

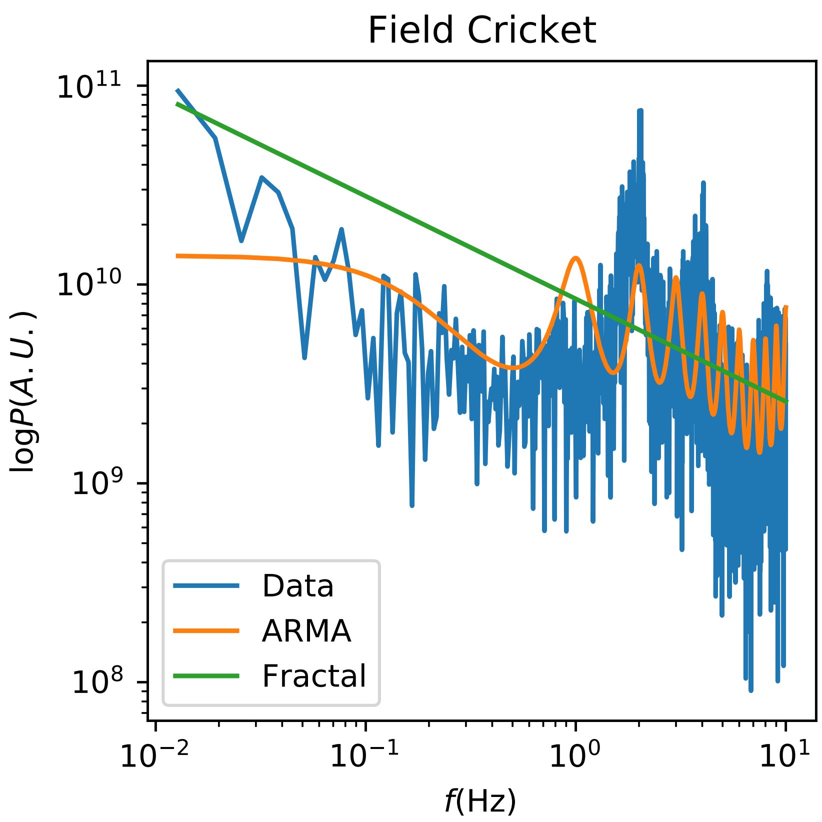

Figure S3: The spectrum of Recording 191240 of the Field Cricket is shown along with the best-fit A and fractal spectral models.

References

[1] Rossum G. Python Reference Manual. Amsterdam, The Netherlands, The Netherlands; 1995.

[2] van der Walt S, Colbert SC, Varoquaux G. The NumPy Array: A Structure for Efficient Numerical Computation. Comput Sci Eng. 2011 March;13(2):22–30.

[3] Jones E, Oliphant T, Peterson P, et al.. SciPy: Open source scientific tools for Python; 2001–. [Online; accessed August 12, 2018]. Available from: `http://www.scipy.org/`.

[4] Hunter JD. Matplotlib: A 2D Graphics Environment. Comput Sci Eng. 2007 May;9(3):90–95.
